# Novel 3,4-Dihydroxyphenyl-Thiazole-Coumarin Hybrid Compounds: Synthesis, In Silico and In Vitro Evaluation of Their Antioxidant Activity

**DOI:** 10.3390/antiox14060636

**Published:** 2025-05-26

**Authors:** Daniel Ungureanu, Gabriel Marc, Brîndușa Tiperciuc, Cristina Moldovan, Ioana Ionuț, Anca Stana, Ilioara Oniga, Laurian Vlase, Adrian Pîrnău, Ovidiu Oniga

**Affiliations:** 1 Department of Pharmaceutical Chemistry, “Iuliu Hațieganu” University of Medicine and Pharmacy, 41 Victor Babeș Street, 400012 Cluj-Napoca, Romania; daniel.ungureanu@elearn.umfcluj.ro (D.U.); btiperciuc@umfcluj.ro (B.T.); cmoldovan@umfcluj.ro (C.M.); ionut.ioana@umfcluj.ro (I.I.); stana.anca@umfcluj.ro (A.S.); ooniga@umfcluj.ro (O.O.); 2 Department of Organic Chemistry, “Iuliu Hațieganu” University of Medicine and Pharmacy, 41 Victor Babeș Street, 400012 Cluj-Napoca, Romania; 3 Department of Pharmacognosy, “Iuliu Hațieganu” University of Medicine and Pharmacy, 12 Ion Creangă Street, 400010 Cluj-Napoca, Romania; ioniga@umfcluj.ro; 4 Department of Pharmaceutical Technology and Biopharmacy, “Iuliu Hațieganu” University of Medicine and Pharmacy, 41 Victor Babeș Street, 400012 Cluj-Napoca, Romania; laurian.vlase@umfcluj.ro; 5 National Institute for Research and Development of Isotopic and Molecular Technologies, 67-103 Donath Street, 400293 Cluj-Napoca, Romania; apirnau@itim-cj.ro

**Keywords:** thiazole, coumarin, catechol, hydrazone, hybrid compounds, antioxidant

## Abstract

Oxidative stress represents a key process in many physiopathological mechanisms involved in several diseases. Therefore, antioxidants represent an important strategy in reversing this pathologic process. In the present study, we proposed the design, synthesis, computational, and in vitro evaluation of seven novel hydroxyphenyl–thiazole–coumarin hybrid compounds (**4a**–**g**) as antioxidant molecules. The conducted theoretical quantum and thermodynamical calculations revealed compound **4f** as the most promising antioxidant, having the highest HOMO–LUMO gaps (3.13 eV in vacuum, 3.22 eV in nonpolar environment, and 3.33 in water) and some of the lowest BDE values (68.23 kcal/mol and 69.63 kcal/mol for O-H and N-H bonds in nonpolar environment). This was transposed in the results obtained following the in vitro antiradical (DPPH^•^ and ABTS^•+^) and electron transfer capacity assays (TAC, RP, FRAP, and CUPRAC), although all compounds showed important antioxidant activity, superior in almost all instances to ascorbic acid and Trolox, which were used as references. Compounds **4f** and **4g** can serve as molecules for further research involving in vivo antioxidant activity and possible synergistic mechanisms.

## 1. Introduction

Oxidative stress plays a negative role in the pathology of several conditions, such as diabetes and its microvascular complications, obesity, asthma, cancer, neurodegenerative disorders, and rheumatoid arthritis. The cause of oxidative stress is an imbalance between pro- and antioxidant species, thus resulting in an excessive accumulation of reactive oxygen species (ROS) and reactive nitrogen species (RNS), which cause cellular damage by affecting lipids, proteins, and nucleic acids. The latter two are converted into advanced glycation end products (AGE), which are present in the aforementioned pathologies. However, it is important to mention that a limited amount of ROS and RNS are produced during physiological processes; this is considered an oxidative eustress, and only if the produced amounts exceed the physiological limits, can it be considered an oxidative distress [1,2,3,4,5,6,7].

Antioxidants are compounds that reduce or stop the oxidation of a substrate and their use can aid the treatment of various conditions. Therefore, antioxidant therapies, which are based on the use of antioxidants from supplements or diet, combined with aerobic exercise, have been proposed for reversing oxidative stress [6,8,9].

**Figure 1:**
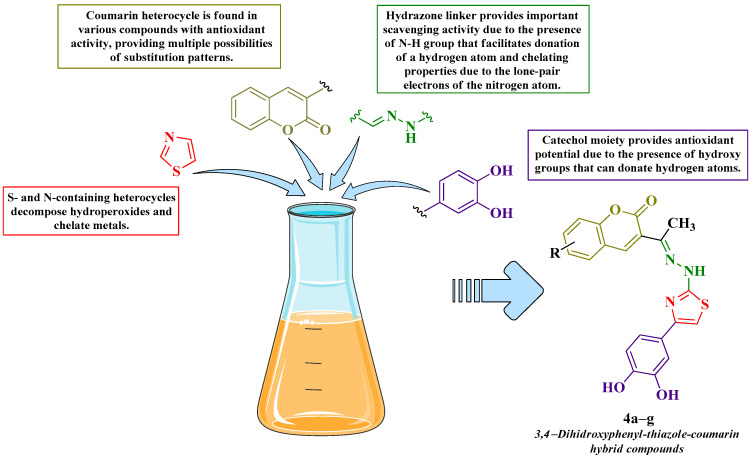
Chemical design of 3,4-dihydroxyphenyl-thiazole-coumarin hybrid compounds (**4a**–**g**) as antioxidants.

Taking into consideration the benefits of the antioxidant compounds in various medical conditions, we have proposed the design, synthesis, and characterization of seven hydroxyphenyl–thiazolyl–coumarin compounds (**4a**–**g**) with antioxidant potential. Our new compounds contain a thiazole heterocycle substituted in the fourth position with a catechol moiety and in the second position with a variously substituted coumarin heterocycle, indirectly attached to the thiazole through a hydrazonoethyl linker (Figure 1).

The thiazole heterocycle is present in various reported antioxidant compounds, and it has been shown that the inclusion of S- and N-containing heterocycles in potential antioxidants helps decompose hydroperoxides and chelating metals [10,11,12]. The inclusion of a coumarin heterocycle was motivated by its presence in many reported compounds with antioxidant activity due to the great number of possible substitution patterns [13,14,15,16,17]. The hydrazonoethyl linker provides important scavenging activity due to the presence of the N-H group that facilitates donation of a hydrogen atom and chelating properties due to the lone-pair electrons of the nitrogen atom [18,19,20,21]. Finally, any potent antioxidant compound contains phenolic groups, as observed in natural antioxidants, thus motivating the inclusion of a catechol moiety in the proposed compounds [22].

The biological evaluation was preceded by in silico studies consisting of druggability predictions and theoretical quantum and thermodynamical calculations to predict the release of electrons and hydrogen atom transfer (HAT). The in vitro studies consisted of antiradical, electron transfer capacity, and ferrous ions chelation assays for evaluating the antioxidant potential of the newly synthesized compounds. Based on the results, several points are discussed and conclusions are drawn.

## 2. Materials and Methods

### 2.1. Chemistry

All the necessary laboratory glassware, porcelainware, reagents, and solvents used for the chemical synthesis, purification, and spectral analysis were purchased from local suppliers.

The progress of the reactions and the purity of all compounds were verified by thin layer chromatography (TLC), using silica gel 60 F_254_ as stationary phase (Merck KGaA Darmstadt, Germany) and ethyl acetate/heptane in a 7:3 mixture as mobile phase.

The melting point (mp) was determined via thermal optical analysis using an MPM-H1 (Schropp Gerätetechnik, Überlingen, Germany) melting point device using the glass capillary method.

The structures of all intermediate compounds were confirmed using infrared (IR) and mass spectral analysis, while the final compounds were supplementarily confirmed by proton nuclear magnetic resonance (^1^H NMR) and carbon magnetic resonance (^13^C NMR) spectral analyses. The IR spectral analysis was performed on an FT/IR 61600 spectrometer (Jasco, Cremella, Italy) and the samples were prepared in KBr tablets under vacuum. The mass spectra (MS) were recorded using positive and negative ionization modes on an Agilent 1100 series device, connected to an Agilent Ion Trap SL mass spectrometer (Agilent Technologies, Santa Clara, CA, USA), while the samples were dissolved in a mixture of acetonitrile and dimethyl sulfoxide (DMSO). The NMR spectra were recorded using an Avance NMR spectrometer (Bruker, Karlsruhe, Germany). The samples were dissolved in DMSO-*d_6_*, while tetramethylsilane (TMS) was used for calibration, with its peak used as reference for the chemical shifts (ppm) calculation, which were reported in δ units. The identified signal multiplicity was presented using abbreviations for the peak patterns: s–singlet, d–doublet, dd–double doublet, t–triplet, q–quartet, and m–multiplet. To identify an atom in a specific region of the molecule, the following abbreviations were used: Cou–coumarin, Ar–phenyl, and Th–thiazole.

#### 2.1.1. Synthesis of Compounds **2a**–**g**

In a beaker, 5 mmol of variously substituted salicylaldehydes (**1a**–**g**) were dissolved in 5 mmol (0.650 g) of ethyl acetoacetate, with 2–3 drops of piperidine as catalyst. The solution was left at room temperature for 24–48 h, until large crystals were visible. The crystals were filtered, washed with absolute methanol, and dried under vacuum. This protocol represents a slight adaptation of a previously reported method [23]. All intermediate compounds **2a**–**g** were previously reported by other research groups [24,25,26,27,28].

*3-acetyl-2*H-*chromen-2-one* (**2a**): white crystals; mp = 123 °C (lit. 120-122 °C [24]); yield = 59.00%; FTIR (KBr) ν_max_ (cm^−1^): 1741.89 (C=O ester), 1676.80 (C=O ketone), 1614.61 (C=C), 1210.60 (C-O); ESI^+^-MS: *m/z* = 189.0 ([M+H]^+^), 211.0 ([M+Na]^+^).

*3-acetyl-8-methoxy-2*H*-chromen-2-one* (**2b**): white-green crystals; mp = 174 °C (lit. 171–172 °C [25]); yield = 91.56%; FTIR (KBr) ν_max_ (cm^−1^): 1735.14 (C=O ester), 1685.00 (C=O ketone), 1601.11 (C=C), 1281.95 (C-O ether), 1200.47 (C-O ester); ESI^+^-MS: *m*/*z* = 219.0 ([M+H]^+^), 241.0 ([M+Na]^+^).

*3-acetyl-8-ethoxy-2*H*-chromen-2-one* (**2c**): white-green crystals; mp = 137 °C (lit. 122–124 °C [26]); yield = 76.32%; FTIR (KBr) ν_max_ (cm^−1^): 1707.18 (C=O ester), 1630.52 (C=O ketone), 1608.82 (C=C), 1277.73 (C-O ether), 1216.38 (C-O ester); ESI^+^-MS: *m*/*z* = 233.1 ([M+H]^+^).

*3-acetyl-6-bromo-2*H*-chromen-2-one* (**2d**): white-green crystals; mp = 226 °C (lit. 204–205 °C [27]); yield = 33.55%; FTIR (KBr) ν_max_ (cm^−1^): 1735.14 (C=O ester), 1675.36 (C=O ketone), 1608.34 (C=C), 1205.29 (C-O), 559.26 (C-Br); ESI^+^-MS: *m*/*z* = 268.9 ([M+H]^+^).

*2-acetyl-benzo*[f]*chromen-3-one* (**2e**): fluorescent yellow crystals; mp = 181–182 °C [27]; yield = 36.35%; FTIR (KBr) ν_max_ (cm^−1^): 1733.21 (C=O ester), 1675.46 (C=O ketone), 1557.24 (C=C), 1219.27 (C-O); ESI^+^-MS: *m/z* = 239.3 ([M+H]^+^), 261.1 ([M+Na]^+^).

*3-acetyl-6-hydroxy-2*H*-chromen-2-one* (**2f**): yellow crystals; mp = 247–248 °C (lit. 234–236 °C [28]); yield = 75.52%; FTIR (KBr) ν_max_ (cm^−1^): 3173.29 (O-H), 1740.92 (C=O ester), 1644.02 (C=O ketone), 1567.36 (C=C), 1243.38 (C-O); ESI^+^-MS: *m*/*z* = 203.3 ([M-H]^+^).

*3-acetyl-7-hydroxy-2*H-*chromen-2-one* (**2g**): yellow crystals; mp = 237 °C (lit. 240–241 °C [27]); yield = 36.92%; FTIR (KBr) ν_max_ (cm^−1^): 3485.7 (O-H), 1717.78 (C=O ester), 1603.52 (C=O ketone), 1566.88 (C=C), 1215.90 (C-O); ESI^+^-MS: *m*/*z* = 203.7 ([M-H]^+^).

#### 2.1.2. Synthesis of Compounds **3a**–**g**

In a flask, 20 mL of absolute methanol and one drop of concentrated sulfuric acid as catalyst were added over 3.5 mmol of **2a**–**g** and 3.5 mmol (0.318 g) of thiosemicarbazide. The mixture was refluxed under a condenser, using a heating mantle for over 4–5 h. The obtained yellow precipitate was filtered and vacuum dried, followed by recrystallization in hot absolute methanol. This protocol represents a slight adaptation of a previously reported method [23]. All intermediate compounds **3a**–**g** were previously reported [29,30,31,32,33,34].

(E)-*2-(1-(2-oxo-2*H-*chromen-3-yl)ethylidene)hydrazinecarbothioamide* (**3a**): yellow-green crystals; mp = 213–214 °C (lit. 220 °C [29]); yield = 90.41%; FTIR (KBr) ν_max_ (cm^−1^): 3387.35 (N-H), 1718.15 (C=O), 1604.00 (C=C), 1496.97 (C=N), 1428.51 (C-N), 1239.04 (C-O); ESI^+^-MS: *m*/*z* = 262.0 ([M+H]^+^).

(E)-*2-(1-(8-methoxy-2-oxo-2*H-*chromen-3-yl)ethylidene)hydrazinecarbothioamide* (**3b**): yellow-green crystals; mp = 214 °C (lit. 201 °C [30]); yield = 97.70%; FTIR (KBr) ν_max_ (cm^−1^): 3458.71 (N-H); 1714.41 (C=O); 1590.99 (C=C), 1503.72 (C=N), 1428.99 (C-N), 1267.48 (C-O ether), 1210.6 (C-O ester); ESI^+^-MS: *m*/*z* = 292.0 ([M+H]^+^).

(E)-*2-(1-(8-ethoxy-2-oxo-2*H-*chromen-3-yl)ethylidene)hydrazinecarbothioamide* (**3c**): yellow crystals; mp = 221 °C (lit. 216–222 °C [31]); yield = 26.01%; FTIR (KBr) ν_max_ (cm^−1^): 3411.94 (N-H), 1720.19 (C=O), 1613.16 (C=C), 1513.36 (C=N), 1471.90 (C-N), 1278.09 (C-O ether), 1239.04 (C-O ester); ESI^+^-MS: *m*/*z* = 306.1 ([M+H]^+^).

(E)-*2-(1-(6-bromo-2-oxo-2*H-*chromen-3-yl)ethylidene)hydrazinecarbothioamide* (**3d**): white-green solid; carbonization over 240 °C (lit. mp = 234 °C [32]); yield = 43.47%; FTIR (KBr) ν_max_ (cm^−1^): 3420.14 (N-H), 1733.21 (C=O), 1597.25 (C=C), 1494.56 (C-N), 1236.63 (C-O), 538.52 (C-Br); ESI^+^-MS: *m*/*z* = 341.9 ([M+H]^+^).

(E)-*2-(1-(3-oxo-3*H-*benzo*[f]*chromen-2-yl)ethylidene)hydrazinecarbothioamide* (**3e**): fluorescent yellow-green solid; mp = 235 °C (no reported mp value in [33]); yield = 78.53%; FTIR (KBr) ν_max_ (cm^−1^): 3405.19 (N-H), 1716.34 (C=O), 1600.16 (C=C), 1563.50 (C=N), 1491.19 (C-N), 1213.01 (C-O); ESI^+^-MS: *m*/*z* = 312.1 ([M+H]^+^).

(E)-*2-(1-(6-hydroxy-2-oxo-2*H-*chromen-3-yl)ethylidene)hydrazinecarbothioamide* (**3f**): yellow solid; carbonization over 230 °C (lit. mp = 222–224 °C with decomposition [34]); yield = 73.10%; FTIR (KBr) ν_max_ (cm^−1^): 3374.82 (N-H), 3194.02 (O-H), 1725.98 (C=O), 1615.09 (C=C), 1566.40 (C=N), 1505.17 (C-N), 1227.95 (C-O); ESI^+^-MS: *m*/*z* = 276.1 ([M-H]^+^).

(E)-*2-(1-(7-hydroxy-2-oxo-2*H-*chromen-3-yl)ethylidene)hydrazinecarbothioamide* (**3g**): yellow solid; carbonization over 213–214 °C (lit. mp = 222–224 °C with decomposition [34]); yield = 55.85%; FTIR (KBr) ν_max_ (cm^−1^): 3375.30 (N-H), 3176.67 (O-H), 1678.25 (C=O), 1617.50 (C=C), 1586.65 (C=N), 1482.51 (C-N), 1236.15 (C-O); ESI^+^-MS: *m*/*z* = 276.3 ([M-H]^+^).

#### 2.1.3. Synthesis of Compounds **4a**–**g**

In a flask, 50 mL of acetone was added to 2 mmol of **3a**–**g** and 3 mmol (0.559 g) of 2-chloro-1-(3,4-dihydroxyphenyl)ethanone. The mixture was refluxed under a condenser using a heating mantle for 5–6 h. The obtained precipitate was filtered and vacuum dried, followed by recrystallization in hot absolute methanol. This protocol represents a slight adaptation of a previously reported method [35,36,37,38].

(E)-*1-(4-(3,4-dihydroxyphenyl)thiazol-2-yl)-2-(1-(2-oxo-2*H*-chromen-3-yl)ethylidene)hydrazin-1-ium chloride* (**4a**): yellow solid; carbonization over 230 °C; yield = 34.06%; FTIR (KBr) ν_max_ (cm^−1^): 3482.33 (N-H), 3126.04 (O-H), 1720.67 (C=O), 1606.90 (C=C), 1571.70 (C=N), 1521.56 (C-N), 1237.11 (C-O), 778.14 (C-S); ESI^+^-MS: *m*/*z* 392.7 ([M-H]^+^); ^1^H-NMR (DMSO-*d_6_*, 500 MHz) δ (ppm): 8.190 (s, 1H, Cou), 7.872–7.859 (d, 1H, Cou, *J* = 6.5 Hz), 7.664–7.637 (t, 1H, Cou, *J* = 6.5 and 7 Hz), 7.454–7.437 (d, 1H, Cou, *J* = 8.5 Hz), 7.408–7.378 (t, 1H, Cou, *J* = 7.5 Hz), 7.248 (s, 1H, Th), 7.149–6.77 (m, 3H, Ar), 2.301 (s, 3H, -CH_3_); ^13^C-NMR (DMSO-*d_6_*, 125 MHz) δ (ppm): 168.983 (Th), 159.107 (C=O), 153.304 (Th), 145.591 (Ar), 145.269 (Ar), 140.902 (Cou), 132.278 (Cou), 129.136 (Cou), 126.371 (Ar), 124.754 (Ar), 118.882 (Cou), 117.125 (Cou), 115.970 (Cou), 115.711 (Cou), 113.380 (Th), 101.370 (Ar), 16.342 (-CH_3_).

(E)-*1-(4-(3,4-dihydroxyphenyl)thiazol-2-yl)-2-(1-(8-methoxy-2-oxo-2*H*-chromen-3-yl)ethylidene)hydrazin-1-ium chloride* (**4b**): yellow solid; carbonization over 223 °C; yield = 59.00%; FTIR (KBr) ν_max_ (cm^−1^): 3420.14 (N-H), 3215.24 (O-H), 1686.93 (C=O), 1621.36 (C=C), 1573.15 (C=N), 1508.06 (C-N), 1284.36 (C-O ether), 1238.08 (C-O ester), 772.35 (C-S); ESI^+^-MS: *m*/*z* 422.6 ([M-H]^+^); ^1^H-NMR (DMSO-*d_6_*, 500 MHz) δ (ppm): 8.145 (s, 1H, Cou), 7.345–7.329 (m, 2H, Cou), 7.256–7.252 (d, 1H, Ar, *J* = 2 Hz), 7.155–7.135 (dd, 1H, Ar, *J* = 2 and 5.5 Hz), 6.997 (s, 1s, Th), 6.801–6.762 (dd, 2H, Ar, *J* = 8 and 3.5 Hz), 3.935 (s, 3H, -OCH_3_), 2.287 (s, 3H, -CH_3_); ^13^C-NMR (DMSO-*d_6_*, 125 MHz) δ (ppm): 168.920 (Th), 158.862 (C=O), 146.263 (Ar), 145.374 (Ar), 145.192 (Cou), 142.595 (Cou), 140.923 (Cou), 126.602 (Ar), 124.670 (Ar), 120.219 (Cou), 119.463 (Cou), 117.069 (Cou), 115.648 (Cou), 114.451 (Ar), 113.345 (Th), 101.272 (Ar), 56.126 (-OCH_3_), 16.216 (-CH_3_).

(E)-*1-(4-(3,4-dihydroxyphenyl)thiazol-2-yl)-2-(1-(8-ethoxy-2-oxo-2*H*-chromen-3-yl)ethylidene)hydrazin-1-ium chloride* (**4c**): yellow solid; carbonization over 188 °C; yield = 99.50%; FTIR (KBr) ν_max_ (cm^−1^): 3356.50 (N-H), 3064.00 (O-H), 1716.34 (C=O ester), 1604.97 (C=C); 1510.47 (C-N); 1283.88 (C-O ether); 1207.70 (C-O ester); 770.90 (C-S); ESI^+^-MS: *m*/*z* 436.2 ([M-H]^+^); ^1^H-NMR (DMSO-*d_6_*, 500 MHz) δ (ppm): 8.146 (s, 1H, Cou), 7.400–7.382 (dd, 1H, Cou, *J* = 3 and 5 Hz), 7.327–7.278 (m, 2H, Cou, *J* = 3 Hz), 7.253–7.249 (d, 1H, Ar, *J* = 2 Hz), 7.151–7.131 (dd, 1H, Ar, *J* = 2 and 6 Hz), 7.007 (s, 1H, Th), 6.786–6.770 (d, 1H, Ar, *J* = 8 Hz), 4.216–4.174 (q, 2H, -CH_2_-, *J* = 7 Hz), 2.293 (s, 3H, -CH_3_), 1.432–1.404 (t, 3H, -CH_3_, *J* = 7 Hz); ^13^C-NMR (DMSO-*d_6_*, 125 MHz) δ (ppm): 168.948 (Th), 158.925 (C=O), 145.493 (Ar), 145.234 (Ar), 142.714 (Cou), 141.063 (Cou), 126.483 (Ar), 124.677 (Ar), 120.191 (Cou), 119.540 (Cou), 117.097 (Cou), 115.676 (Cou), 115.368 (Ar), 113.366 (Th), 101.314 (Ar), 64.420 (-CH_2_), 30.684 (-CH_3_), 16.279 (-CH_3_).

(E)-*1-(4-(3,4-dihydroxyphenyl)thiazol-2-yl)-2-(1-(6-bromo-2-oxo-2*H*-chromen-3-yl)ethylidene)hydrazin-1-ium chloride* (**4d**): yellow solid; carbonization over 252 °C; yield = 46.29%; FTIR (KBr) ν_max_ (cm^−1^): 3421.10 (N-H), 3073.96 (O-H), 1701.87 (C=O), 1617.98 (C=C), 1598.70 (C=N), 1523.49 (C-N), 1212.04 (C-O), 785.85 (C-S), 591.56 (C-Br); ESI^+^-MS: *m*/*z* 469.8 ([M-H]^+^); ^1^H-NMR (DMSO-*d_6_*, 500 MHz) δ (ppm): 8.147 (s, 2H, Cou), 7.794–7.771 (dd, 1H, Cou, *J* = 2.5 and 6.5 Hz), 7.425–7.408 (d, 1H, Cou, *J* = 8.5 Hz), 7.256–7.252 (d, 1H, Ar, *J* = 2 Hz), 7.154–7.133 (d, 1H, Ar, *J* = 6 Hz), 7.017 (s, 1H, Th), 6.783–6.767 (d, 1H, Ar, *J* = 8 Hz), 2.284 (s, 3H, -CH_3_); ^13^C-NMR (DMSO-*d_6_*, 125 MHz) δ (ppm): 168.892 (Th), 158.673 (C=O), 152.324 (Th), 145.458 (Ar), 145.227 (Ar), 139.355 (Cou), 134.420 (Cou), 131.040 (Cou), 127.477 (Ar), 125.762 (Ar), 120.863 (Cou), 118.217 (Cou), 117.083 (Cou), 116.250 (Cou), 115.676 (Cou), 113.359 (Th), 101.398 (Ar), 16.202 (-CH_3_).

(E)-*1-(4-(3,4-dihydroxyphenyl)thiazol-2-yl)-2-(1-(3-oxo-3*H*-benzo*[*f*]*chromen-2-yl)ethylidene)hydrazin-1-ium chloride* (**4e**): orange solid; carbonization over 260 °C; yield = 64.24%; FTIR (KBr) ν_max_ (cm^−1^): 3404.23 (N-H), 3129.90 (O-H), 1696.57 (C=O), 1609.79 (C=C), 1566.88 (C=N), 1510.47 (C-N); 1214.45 (C-O); 781.51 (C-S); ESI^+^-MS: *m*/*z* 441.8 ([M-H]^+^); ^1^H-NMR (DMSO-*d_6_*, 500 MHz) δ (ppm): 8.918 (s, 1H, Cou), 8.561–8.544 (d, 1H, Cou, *J* = 8.5 Hz), 8.239–8.221 (d, 1H, Cou, *J* = 9 Hz), 8.099–8.083 (d, 1H, Cou, *J* = 8 Hz), 7.803–7.770 (m, 1H, Cou), 7.673–7.612 (m, 2H, Cou), 7.272–7.267 (d, 1H, Ar, *J* = 2.5 Hz), 7.171–7.151 (dd, 1H, Ar, *J* = 2 and 6 Hz), 7.014 (s, 1H, Th), 6.788–6.772 (d, 1H, Ar, *J* = 8 Hz), 2.370 (s, 3H, -CH_3_); ^13^C-NMR (DMSO-*d_6_*, 125 MHz) δ (ppm): 169.081 (Th), 159.107 (C=O), 153.213 (Th), 145.416 (Ar), 145.213 (Ar), 136.527 (Cou), 133.545 (Cou), 129.955 (Cou), 128.856 (Cou), 128.576 (Cou), 126.217 (Ar), 125.496 (Ar), 122.094 (Cou), 117.097 (Cou), 116.439 (Cou), 115.655 (Cou), 113.366 (Cou), 112.932 (Th), 101.384 (Ar), 16.237 (-CH_3_).

(E)-*1-(4-(3,4-dihydroxyphenyl)thiazol-2-yl)-2-(1-(6-hydroxy-2-oxo-2*H*-chromen-3-yl)ethylidene)hydrazin-1-ium chloride* (**4f**): yellow solid; carbonization over 225 °C; yield = 47.18%; FTIR (KBr) ν_max_ (cm^−1^): 3423.99 (N-H), 3070.12 (O-H), 1691.27 (C=O), 1612.30 (C=C), 1563.02 (C=N), 1510.95 (C-N). 1223.61 (C-O); 774.76 (C-S); ESI^+^-MS: *m*/*z* 407.9 ([M-H]^+^); ^1^H-NMR (DMSO-*d_6_*, 500 MHz) δ (ppm): 8.066 (s, 1H, Cou), 7.295–7.277 (d, 1H, Cou, *J* = 9 Hz), 7.255–7.250 (d, 1H, Cou, *J* = 2.5 Hz), 7.16–7.14 (dd, 2H, Ar, *J* = 2.5 and 6 Hz), 7.091–7.067 (dd, 1H, Cou, *J* = 6.5 and 3 Hz), 6.991 (s, 1H, Th), 6.774–6.757 (d, 1H, Ar, *J* = 8.5 Hz), 2.270 (s, 3H, -CH_3_); ^13^C-NMR (DMSO-*d_6_*, 125 MHz) δ (ppm): 168.934 (Th), 159.394 (C=O), 153.948 (Th), 146.662 (Cou), 145.360 (Ar), 145.178 (Ar), 140.748 (Cou), 126.539 (Ar), 120.303 (Ar), 119.407 (Cou), 117.069 (Cou), 116.831 (Cou), 115.627 (Cou), 113.345 (Cou), 112.876 (Th), 101.258 (Ar), 16.307 (-CH_3_).

(E)-*1-(4-(3,4-dihydroxyphenyl)thiazol-2-yl)-2-(1-(7-hydroxy-2-oxo-2*H*-chromen-3-yl)ethylidene)hydrazin-1-ium chloride* (**4g**): dark red solid; carbonization over 220 °C; yield = 42.25%; FTIR (KBr) ν_max_ (cm^−1^): 3423.99 (N-H), 3121.22 (O-H), 1711.51 (C=O), 1606.41 (C=C), 1570.74 (C=N), 1488.78 (C-N), 774.76 (C-S); ESI^+^-MS: *m*/*z* 407.1 ([M-H]^+^); ^1^H-NMR (DMSO-*d_6_*, 500 MHz) δ (ppm): 8.063 (s, 1H, Cou), 7.689–7.672 (d, 1H, Cou, *J* = 8.5 Hz), 7.250–7.246 (d, 1H, Cou, *J* = 2 Hz), 7.153–7.132 (dd, 1H, Cou, *J* = 2 and 6.5 Hz), 6.972 (s, 1H, Th), 6.849–6.828 (dd, 1H, Ar, *J* = 2, 1, and 2.5 Hz), 6.772–6.750 (m, 2H, Ar), 2.260 (s, 1H, -CH_3_); ^13^C-NMR (DMSO-*d_6_*, 125 MHz) δ (ppm): 169.004 (Th), 161.745 (Cou), 159.576 (C=O), 155.355 (Th), 145.311 (Ar), 145.164 (Ar), 141.349 (Cou), 130.508 (Ar), 121.814 (Ar), 117.048 (Cou), 115.606 (Cou), 113.611 (Cou), 113.324 (Th), 111.344 (Cou), 101.797 (Ar), 16.272 (-CH_3_).

### 2.2. In Silico Studies

#### 2.2.1. Druggability Prediction

The in silico prediction of the druggability for compounds **4a**–**g** was performed using the SwissADME (http://www.swissadme.ch/, accessed on 20 February 2024) and admetSAR 2.0 web tools [39,40,41].

The physicochemical descriptors taken into consideration were the molecular weight (MW), the number of rotatable bonds (RB), the number of H-bond acceptors (HBA), the number of H-bond donors (HBD), the topological polar surface area (TPSA), the octanol–water partition coefficient implemented by Moriguchi (MLogP), the estimated solubility (ESOL), and the number of violations of Lipinski’s rule of five [42,43,44,45,46,47].

#### 2.2.2. Theoretical Quantum and Thermodynamical Calculations

For the compounds **4a**–**g**, in agreement with their structure, two mechanisms were studied in silico: the release of electrons and the hydrogen atom transfer (HAT). The ease of release of electrons from molecules is characterized by the energy levels of the frontier molecular orbitals HOMO (Highest Occupied Molecular Orbital) and LUMO (Lowest Unoccupied Molecular Orbital). The ease of HAT from molecules is characterized by the Bond Dissociation Enthalpy (BDE), computed on the homolytic cleavage of the atom-H bond [14,48,49,50].

The calculations for the studied compounds, **4a**–**g**, were performed using Spartan’24 (Wavefunction, CA, USA) at the B3LYP level of theory with the 6-31+G* basis set.

To evaluate how the solvent’s polarity affects the antioxidant activity of the compounds, calculations were conducted for vacuum, nonpolar solvent, and water as environmental factors.

### 2.3. In Vitro Studies

The stock solutions of both the reference antioxidants (ascorbic acid and Trolox) and compounds **4a**–**g** were prepared by dissolving the respective solid powders in DMSO to achieve a final concentration of 2 mM. Subsequently, a series of diluted solutions (0.2 mM) for each compound was prepared by further dilution in DMSO. The absorbance measurements were conducted using a Specord 210 PLUS UV-Vis spectrophotometer (Analytik Jena AG, Jena, Germany) with low-volume, single-use 10 mm cuvettes. Preliminary absorption spectra of the compounds, recorded over a wavelength range of 400 nm to 800 nm, showed no significant absorption peaks near the wavelengths used in the assays. All measurements were carried out with blank samples as references. To determine the activity of compounds **4a**–**g**, the half-maximal inhibitory concentration (IC_50_) values for the antiradical assays (DPPH and ABTS) and ferrous ion chelation were calculated using Equation (1). Additionally, for the electron transfer assays, the equivalent (Eq) molar activity for each compound was calculated using Equation (2).

(1) $$radical\;scavenging(\%)=\frac{control\;absorbance-sample\;absorbance}{control\;absorbance}\times 100$$

(2) $$equivalents\;of\;control=\frac{sample\;absorbance}{control\;absorbance}\times 100$$

#### 2.3.1. Antiradical Assays

The DPPH^•^ radical scavenging assay protocol was adapted from Brand-Williams et al. [51]. A volume of 100 μL of sample solutions at various concentrations were mixed with 1 mL of DPPH reagent and thoroughly mixed. The reaction mixture was incubated in the dark for 30 min, following the protocol described in the literature [52,53].

The decolorization assay of the green ABTS^•+^ was performed following the method described by Re et al. [54], as outlined in our group’s previous publication. The reagent, prepared in potassium phosphate buffer (0.1 M, pH = 7.4), was activated overnight with manganese dioxide (MnO_2_). Its stability was confirmed by maintaining a consistent absorbance of approximately 0.7 at 734 nm over a one-hour period prior to use. In all cuvettes, 150 μL of the tested compound solutions at increasing concentrations were combined with 2000 µL of ABTS^•+^ reagent. The mixtures were thoroughly shaken for 10 min at room temperature, protected from light. Following this, the absorbance of the solutions was measured spectrophotometrically at λ = 734 nm [52,53].

#### 2.3.2. Electron Transfer Capacity Assays

The Total Antioxidant Capacity (TAC) assay was performed according to the methodology described in our previous publication, which builds on initial reports from the literature. In sealed glass test tubes, 10 mL of reagent was mixed with 1000 µL of the compound- and standard-based solutions, followed by incubation in a water bath at 95 °C for 90 min. After cooling the test tubes to room temperature, 1 mL of each solution was diluted with 1 mL of water, and the absorbance was measured at λ = 695 nm [52,53,55,56].

The Reducing Power (RP) assay protocol employed is an adaptation of previously reported methodologies [52,53]. In glass test tubes, 1000 µL of solutions containing the compounds and standards was combined with 400 µL of phosphate buffer (0.2 M, pH = 6.6) and 400 µL of K_3_[Fe(CN)_6_] solution (1% *w*/*v*). The tubes were then sealed and incubated in a water bath at 50 °C for 20 min. After cooling to room temperature, 500 µL of trichloroacetic acid (10% *w*/*w*) was added to each test tube, and the mixtures were allowed to stand for 30 min. Subsequently, 250 µL of the resulting solutions was mixed with 140 µL of FeCl_3_ solution (0.1% *w*/*v*) and 1000 µL of distilled water, after which the absorbance was measured at λ = 695 nm.

In the Ferric Reducing Antioxidant Potential (FRAP) assay, 500 µL of solutions containing the compounds and reference antioxidants was mixed with 600 µL of FRAP reagent and 1200 µL of acetate buffer (0.3 M, pH = 3.6), as reported in the literature. The resulting solutions were thoroughly mixed in the dark, and their absorbance was spectrophotometrically measured at λ = 593 nm [52,53,57].

The electron-donating capacity of the compounds was evaluated using the CUPRAC (CUPric Reducing Antioxidant Capacity) method, following a protocol adapted from the original reports [52,53,58,59]. In test tubes, 250 µL of 10 mM copper(II) chloride (CuCl_2_), 1 mL of 1 M ammonium acetate buffer, and 250 µL of 7.5 mM neocuproine ethanolic solution were thoroughly mixed with 125 µL of the sample and reference compound solutions. The mixtures were shaken for 30 min in the dark, after which their absorbance was measured against a blank sample at a wavelength λ = 450 nm.

#### 2.3.3. Ferrous Ions Chelation Assay

The Ferrous Ions Chelation assay is based on an adaptation of the initial report of Dinis et al. [60]. In this assay, the solutions of compounds (2 mM) were mixed with 500 µL of 0.125 mM ferrous sulphate (FeSO_4_) and 500 µL of 0.315 mM ferrozine. The obtained samples were left at room temperature for 10 min, after which their absorbance was measured at λ = 562 nm, using ethylenediaminetetraacetic acid (EDTA) disodium salt (EDTA-Na_2_) as positive control [52,53,61,62,63].

## 3. Results

### 3.1. Chemical Synthesis

**Scheme 1:**
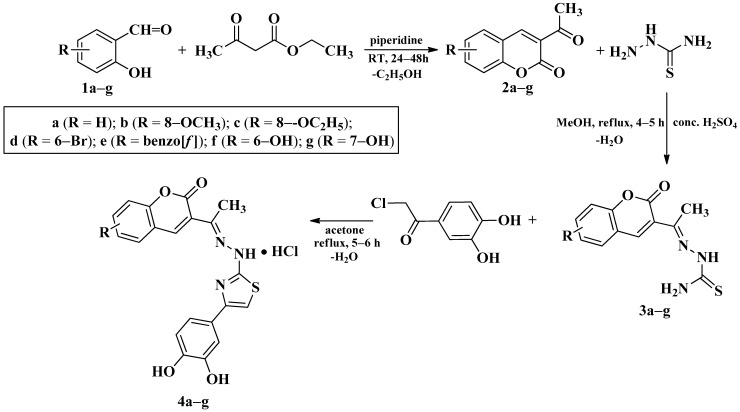
The general synthetic process of compounds **4a**–**g**. Legend: RT = room temperature; MeOH = methanol; conc. = concentrated.

A total of seven novel compounds **4a**–**g** were obtained from the condensation of ethyl acetoacetate with various salicylaldehydes **1a**–**g**, followed by further derivatization of the 3-acetylcoumarins **2a**–**g** with thiosemicarbazide and the condensation of coumarin-3-yl-thiosemicarbazones **3a**–**g** with 2-chloro-1-(3,4-dihydroxyphenyl)ethanone (Scheme 1).

The novel compounds were obtained in yields between 34.06–99.50% and their structural identity was confirmed through IR, MS, ^1^H-, and ^13^C-NMR spectral analyses. The progress of chemical reactions was verified using TLC and R_f_ determinations.

The graphical representations of all recorded IR, MS, and NMR spectra for the compounds **4a**–**g** and their intermediates are illustrated in Figures S1–S56 from Supplementary Materials.

### 3.2. In Silico Studies

#### 3.2.1. Druggability Prediction

The computed in silico physicochemical descriptors for compounds **4a**–**g** are presented in Table 1. The molecular weight was between 394.42 g/mol and 473.32 g/mol and the number of rotatable bonds was between four and six. Each compound had seven to eight HBAs and three to four HBDs, while the value of TPSA was between 140.77 Å^2^ and 161.00 Å^2^. The computed MLogP for every compound had a negative value, while the ESOL was predicted to be between 0.18 µg/mL and 3.07 µg/mL. All compounds had no violations of Lipinski’s rule of five.

#### 3.2.2. Theoretical Quantum and Thermodynamical Calculations

In all compounds **4a**–**g**, the HOMO was identified over the thiazolyl-catechol region, while LUMO was identified over the 2*H*-chromen-2-one (Figures S64–S70 from Supplementary Materials). The level of energy of the two frontier orbitals in vacuum, nonpolar environment, and water are presented in Table 2.

**Figure 2:**
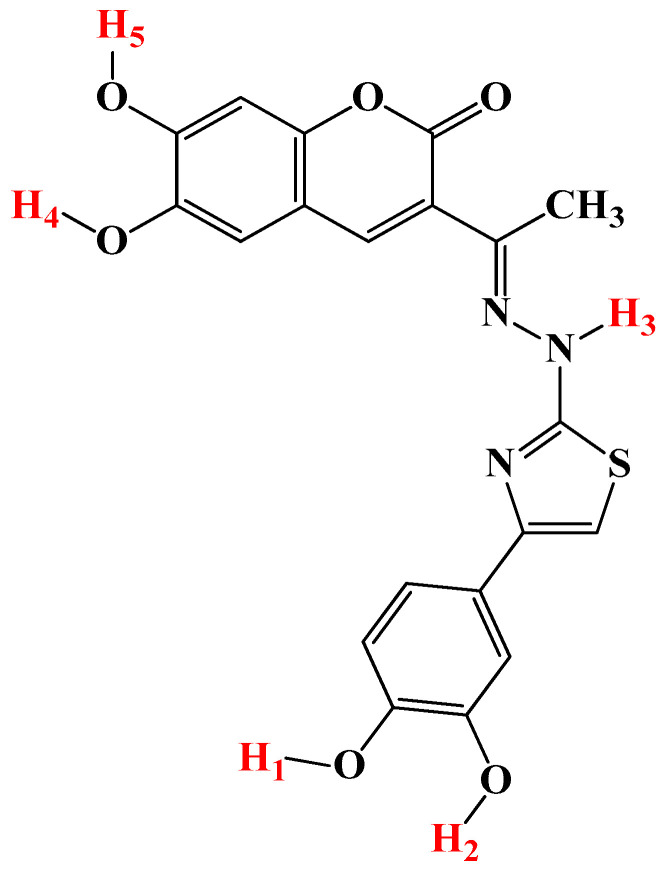
The general structure of compounds **4a**–**g**, with highlight on sites which can easily release hydrogen atoms. Legend: H_1_—hydrogen in the 3-hydroxy group, H_2_—hydrogen in the 4-hydroxy group, H_3_—hydrogen in the hydrazone group, H_4_—hydrogen in the 6-hydroxy group, H_5_—hydrogen in the 7-hydroxy group.

The bond dissociation was computed according to the possible sites of hydrogen atom release by homolytic breaking of heteroatom (N/O)-H bond, as exemplified in Figure 2. The amount of energy needed to break the respective bonds was computed in vacuum, nonpolar environment, and water (Table 3).

The HOMO, LUMO, electrostatic potential map, and spin density map for each of the compounds **4a**–**g** are available in the Supplementary Materials (Figures S57–S70).

### 3.3. In Vitro Studies

#### 3.3.1. Antiradical Assays

The antiradical capacity of the compounds **4a**–**g** was evaluated against two different radicals, DPPH^•^ and ABTS^•+^, compared to ascorbic acid and Trolox. The results are presented in Table 4 as IC_50_ values.

#### 3.3.2. Electron Transfer Capacity Assays

The evaluation of the electron transfer capacity of the compounds **4a**–**g** was conducted using four methods, involving various oxidizing agents and environmental factors (TAC, RP, FRAP, and CUPRAC) and the results of the assays are presented in Table 5 as molar Eq of reference antioxidants.

#### 3.3.3. Ferrous Ions Chelation Assay

The evaluation of the ferrous ions chelation ability of compounds **4a**–**g** was performed in the presence of ferrozine as a chromogenic chelator and using EDTA-Na_2_ as reference. Following this assay, all compounds showed a negligible chelation capacity compared to EDTA-Na_2_.

## 4. Discussion

### 4.1. Chemical Synthesis

All intermediate compounds **2a**–**g** and **3a**–**g** were previously reported in the literature by other research groups [23,24,25,26,27,28,29,30,31,32,33,34]. All final products **4a**–**g** have not been previously reported in the literature. Both intermediate compounds **3a**–**g** and final compounds **4a**–**g** were mainly obtained in the *E* configuration, considering it is the most energy-favorable [64].

The structures of intermediate 3-acetylcoumarins **2a**–**g** were confirmed by spectral analysis. According to the IR spectra, all intermediates showed additional νC=C bands between 1577.24 cm^−1^ and 1644.02 cm^−1^ and νC-O ester bands between 1200.47 cm^−1^ and 1243.38 cm^−1^, which confirmed the first reaction step was completed. Further confirmation was supported by the MS spectra, in which all the corresponding molecular peaks were identified.

The structures of intermediate coumarin-3-yl-ethylidenethiosemicarbazones **3a**–**g** were confirmed by spectral analysis. Based on the IR spectra, all intermediates showed additional νN-H wide stretching bands between 3458.71 cm^−1^ and 3374.82 cm^−1^, νC=N between 1586.65 cm^−1^ and 1503.72 cm^−1^, νC-N between 1505.17 cm^−1^ and 1428.51 cm^−1^, and the disappearance of the broad νC=O ketone bands, between 1685.00 cm^−1^ and 1603.52 cm^−1^. The MS spectra further confirmed the identity of the compounds, after the identification of all the corresponding molecular peaks.

The final products **4a**–**g** were structurally confirmed by extended spectral analysis. According to the IR spectra, all compounds had an additional wide νO-H stretching band in the region between 3064.06 cm^−1^ and 3129.90 cm^−1^ compared to the intermediates **3a**–**g**, meaning that the attachment of the catechol moiety to the final structure was successful. The most compelling evidence was provided by the MS spectra, where all the corresponding molecular peaks were identified, and by the ^1^H- and ^13^C-NMR spectra, where all the expected proton signals and the expected signals for the carbon atoms were identified in the corresponding regions of the spectra and with the expected multiplicity.

### 4.2. In Silico Studies

#### 4.2.1. Druggability Prediction

Based on the computed physicochemical descriptors, all compounds **4a**–**g** respected Lipinski’s rule of five, meaning that no compound had a molecular weight higher than 500 g/mol, the number of HBA (O and N atoms) was lower than 10, the number of HBD (NH and OH groups) was lower than 5, and the value of MLogP was lower than 4.15. From this point of view, compounds **4a**–**g** possess good druggability properties [45,46].

The grafting of methoxy, ethoxy, and hydroxy groups onto the coumarin moiety increased the number of HBD in compounds **4b**, **4c**, **4f**, and **4g**. Additionally, the insertion of methoxy and ethoxy groups increased the number of rotatable bonds in compounds **4b** and **4c**. Compounds **4d** and **4e**, substituted with 6-bromocoumarin and benzo[*f*]coumarin heterocycles, were predicted to have the lowest TPSA and ESOL and the highest MLogP, because of the hydrophobic nature of bromine and the annulation of an additional benzene ring.

In addition, all compounds had negative values of MLogP, due to their chemical state as salts. However, even with their predicted high hydrophilicity, based on the ESOL predictions, all compounds had moderate or poor solubility [39,47].

#### 4.2.2. Theoretical Quantum and Thermodynamical Calculations

Considering the possibility of an extended conjugation on almost the entire molecular structure [65], some of the electronic effects may be influenced by the substitution of a coumarin heterocycle, which can alter the antioxidant potential and energy level of HOMO and LUMO of compounds **4a**–**g**. HOMO energies refer to the compounds’ tendency to donate electrons, while LUMO energies refer to their tendency to accept electrons. The gap between the two orbitals helps to predict the stability and reactivity of the compounds.

In vacuum, the energy of HOMO in all compounds **4a**–**g** lies between −5.09 eV and −5.21 eV, with only small differences across the series. The 6-brominated compound **4d** had the lowest HOMO energy (−5.21 eV), indicating a slightly more stabilized HOMO in vacuum compared to the other compounds, which might suggest a lower tendency to donate electrons and a lower antioxidant potential than the rest of the compounds. Only minor changes in terms of energy of HOMO were identified for the studied compounds in non-polar solvent, showing that the non-polar solvent does not have a major effect on HOMO energy levels. In water, the energy of HOMO slightly decreased for all compounds, indicating a stabilization given by the very polar environment, with minor differences between compounds.

The energy of LUMO in vacuum ranges between −1.96 eV (compound **4g**) to −2.33 eV (compound **4d**, with the most stable LUMO). This suggests that compound **4d** could more readily accept electrons, when compared to the other compounds from the series. The non-polar environment generally increases the energy level of LUMO when compared to vacuum, indicating a destabilization of LUMO in the non-polar medium. The LUMO energies ranged from −1.90 eV (compound **4g**) to −2.22 eV (compound **4d**). In water, the energy of LUMO remained relatively close to that in the non-polar solvent, with some compounds showing small shifts. The LUMO energy of compound **4d** was the lowest at −2.27 eV.

The HOMO–LUMO gaps ranged in vacuum from 2.88 eV (the lowest, compound **4d**) to 3.13 eV (the highest, compound **4g**). The non-polar solvent slightly increased the HOMO–LUMO gaps when compared to vacuum. The gap for compound **4d** increased from 2.88 eV to 3.00 eV, while compound **4g** continued to have the largest gap (3.22 eV). The increase in gap energy can indicate a minor stabilization of both HOMO and LUMO in a non-polar environment. In water, the HOMO–LUMO gaps increased more, with compound **4g** showing the largest gap at 3.33 eV, while compound **4d** had a gap of 3.09 eV. The increase of the polarity of the solvent tended to increase the gap for all compounds, leading to more stability (hardness) and less reactivity (softness).

When analyzing how the substitution of the compounds would influence their electronic properties, they marginally influenced HOMO (identified over thiazolyl-catechol), but significantly influenced LUMO (identified over 2*H*-chromen-2-one, the only point of substitution of compounds **4a**–**g**). It is worth mentioning the HOMO–LUMO gap differences in compounds **4f** and **4g** due to different positions of the -OH groups and the increased gap in compound **4d** due to the presence of bromine substituent.

Compared with other studies computing the antioxidant potential in compounds with similar scaffolds, compounds **4a**–**g** tended to have lower HOMO–LUMO gaps, meaning that they are more reactive. Some of the reported HOMO–LUMO gaps in the literature and in previous studies by our research group ranged between 3.60 and 6.75 eV [14,36,53].

As previously stated in the article, HAT capacity of the compounds was characterized by BDE. For all compounds, due to the homolytic breaking of the studied bonds, it can be seen that the BDE was smaller in a nonpolar environment and the reactivity was increased when compared to vacuum (approximately 0.5 kcal/mol lower). On the other hand, the BDE was higher in water with approximately 3 kcal/mol and the activity was decreased.

Taking into account the bonds O-H_1_, O-H_2_, and N-H_3_ found in all evaluated compounds, it can be observed that the O-H_1_ site was the most favorable to release hydrogen atoms in all compounds and in all tested environments. Hydrazone (N-H_3_ site), which had a BDE with approximately 4 kcal/mol higher than the O-H_1_ in vacuum and approximately 4.6 kcal/mol higher in a nonpolar environment due to the effect of water on its radicalization, would reduce the respective difference at approximately 2.5 kcal/mol between the BDE of O-H_1_ and the BDE of N-H_3_.

In the case of the entire series of compounds, the analysis of the BDE for all the three common sites (O-H_1_, O-H_2_, N-H_3_), in all compounds **4a**–**g** and in any of the studied environments, indicated insignificant differences of reactivity for a given site, independent to the substitution of the 2*H*-chromen-2-one. Therefore, the studied substitutions of 2*H*-chromen-2-one will negligibly influence the reactivity at the level of catechol (O-H_1_ and O-H_2_) or hydrazone (N-H_3_).

The additional phenol group on the 2*H*-chromen-2-one structure in compounds **4f** (group O-H_4_) and **4g** (group O-H_5_) exhibited the highest BDE in this series, surpassing all other studied sites. Therefore, the third phenol group in these compounds is expected to provide a slight increase in antioxidant activity compared to compounds **4a**–**e**, based on the BDE values, although it represents a supplementary hydrogen donor site. Between the two, the O-H_5_ bond in compound **4g** (7-hydroxy-2*H*-chromen-2-one) is expected to break more easily than the O-H_4_ bond in compound **4f** (6-hydroxy-2*H*-chromen-2-one). As for the solvent’s effect on the radicalization of the two hydroxy-2*H*-chromen-2-ones, the same observed trend in the other compounds applies to the additional phenol OH groups: radicalization occurs more readily in vacuum or nonpolar environments and is less favorable in water.

Compared with other studies computing the antioxidant potential in compounds with similar scaffolds, compounds **4a**–**g** tended to have lower BDE values, meaning that they are stronger free radical scavengers. Some of the reported BDE values in the literature ranged between 74.88 and 102.56 kcal/mol [14,48,66].

### 4.3. In Vitro Studies

#### 4.3.1. Antiradical Assays

All tested compounds showed superior antiradical activity against both employed radicals compared to the references. Against the DPPH^•^ radical, the IC_50_ values for compounds **4a**–**g** were lower compared to ascorbic acid (IC_50_ = 50.17 µM) and Trolox (IC_50_ = 36.69 µM), ranging between 23.84–33.49 µM. Similarly, against the ABTS^•+^ radical all compounds had better activity compared to Trolox (IC_50_ = 16.57 µM), with values ranging between 7.06 and 14.04 µM (Table 4).

In both cases, the best antiradical activity was exhibited by compound **4g**, substituted with a 7-hydroxy group on the coumarin heterocycle, closely followed by compound **4f**, substituted with a 6-hydroxy group. Compared to the other five compounds (**4a**–**e**), the enhanced antiradical activity of compounds **4f** and **4g** is due to the supplementary hydroxy group in the molecule, with position 7 being more suitable for the activity since the BDE of 7-OH group (BDE = 76.70 kcal/mol) is lower than the BDE of 6-OH group (BDE = 78.63 kcal/mol). As predicted by the in silico studies, compound **4d** (R = 6-Br) had the lowest antiradical activity, as evidenced by the smaller HOMO-LUMO gap compared to the rest of the compounds, which showed that bromine substitution was not favorable for antiradical activity.

Compared to other studies that conducted antiradical assays, compounds **4a**–**g** showed intermediate results in both assays. Some of the results reported in the literature and in previous studies by our research group ranged between 1.7 and 142.2 μM for the DPPH^•^ radical scavenging assay and between 2.55 and 17.66 μM for the ABTS^•+^ decolorization assay [14,37,38,53].

#### 4.3.2. Electron Transfer Capacity Assays

In the TAC assay, which consisted in the reduction of Mo^6+^ to Mo^5+^, the best electron transfer capacity was observed for compound **4f** (R = 6-OH), closely followed by **4g** (R = 7-OH), both having a greater than two-fold higher potency compared to ascorbic acid (Table 5). In contrast to our expectations, compound **4d** had a better electron transfer capacity than the other four compounds, where the capacity decreased as follows: **4c** > **4a** = **4b** > **4e** (Table 5).

The results following the RP assay, which consisted in the reduction of Fe^3+^ to Fe^2+^, were somewhat different compared to the results from the TAC assay. The best electron transfer capacity was observed for compound **4g** this time, followed by compound **4f**, with a two-fold capacity increase compared to both references. Compounds **4a–c** and **4e** had similar electron transfer capacities, decreasing as follows: **4a** > **4b** > **4c** > **4e**, while the weakest capacity was registered by compound **4d**, which was weaker than that of Trolox (Table 5).

Regarding the FRAP assay, the compounds presented electron transfer capacities in a narrower range, between 1.14 and 1.45 molar Eq Trolox. The best capacity was observed for compound **4f**, followed by **4g**, while the weakest was observed for compound **4d** (Table 5).

Following the CUPRAC assay, all compounds exhibited very good electron transfer capacity. Compounds **4a**–**c** and **4e**–**g** displayed a greater than three-fold capacity increase compared to Trolox, while compound **4d** showed an increase of over two-fold capacity (Table 5).

Compared to other previous studies by our research group in which similar electron transfer capacity assays were conducted in similar conditions and on related compounds, compounds **4a**–**g** showed overall similar results. The reported results ranged between 0.36 and 2.38 molar Eq ascorbic acid for the TAC assay, 1.60 and 4.24 molar Eq ascorbic acid and 1.22 and 3.53 molar Eq Trolox for the RP assay, 0.95 and 1.66 molar Eq Trolox for the FRAP assay, and 1.61 and 6.03 molar Eq Trolox for the CUPRAC assay [53,67].

**Figure 3:**
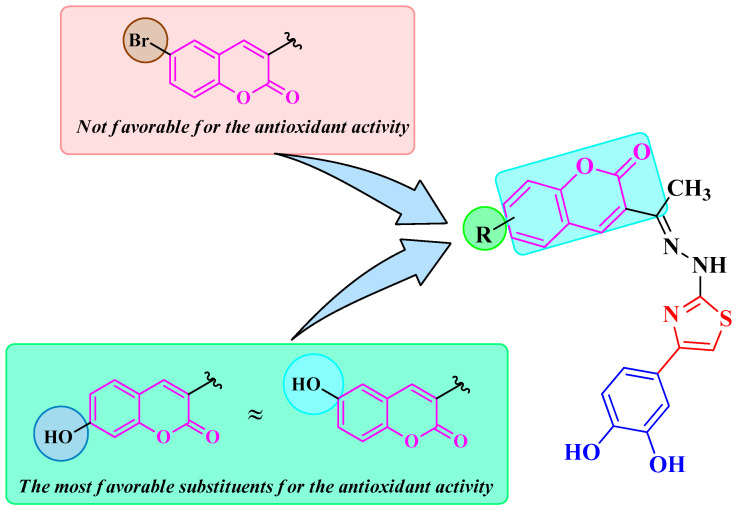
Qualitative structure–antioxidant activity relationships in compounds **4a**–**g**.

Considering the results in both antiradical and electron transfer capacity assays, qualitative structure–antioxidant activity relationships showed that hydroxy group substitutions in compounds **4f** and **4g** were the most favorable for antioxidant activity, while bromine substitution in compound **4d** was the least favorable for the activity (Figure 3). This may be explained by the electron-withdrawing properties of bromine, which decreases the electron density on the coumarin heterocycle and the tendency to donate electrons.

#### 4.3.3. Ferrous Ions Chelation Assay

None of the compounds **4a**–**g** exhibited ferrous ions chelation capacity when compared to EDTA-Na_2_. We aimed at using a different reference compound; however, the literature reports regarding different standards were scarce. We believe that EDTA-Na_2_ is too strong to draw comparisons with synthetic compounds that contain only some chelation sites, compared to natural polyphenolic extracts, which have more chelation sites.

Additionally, another hypothesis for the reduced chelation capacity of compounds **4a**–**g** may be attributed to their existence in *E* configuration, preventing the intramolecular chelation between the C=O group of the coumarin heterocycle and N-H group of the hydrazone linker [68,69].

### 4.4. Limitations

The identified limitations of this study include the absence of some assays that were previously performed by our research group, like the NO radical scavenging assay or the copper ions chelation assay, the absence of calculations for the hardness (η) and chemical potential (μ) parameters in the studied environments, the lack of an appropriate reference chelation agent for the ferrous ions chelation assay, and the absence of an experimental model in cells and/or animals.

However, we considered the last limitation a necessary one, in order to test our hypothesis regarding the antioxidant potential of compounds **4a**–**g** and select the most potent ones for further investigations. Therefore, the obtained results highlight potent antioxidant activity for compounds **4a**–**g**, which could be further explored for the possibility of a synergistic effect between the antioxidant activity of these compounds and a possible antimicrobial or anticancer activity they may have.

## 5. Conclusions

This study illustrated the chemical design and synthesis of seven novel 3,4-dihydroxy–thiazole–coumarin hybrid compounds, followed by in silico studies regarding their antioxidant potential, with the observations being transposed into in vitro assays.

The chemical design of compounds **4a**–**g** consisted of combining structural elements available in reported compounds with antioxidant activity. The chemical synthesis consisted of a three-step process of condensation reactions. All intermediate and final compounds **4a**–**g** were confirmed through IR, MS, ^1^H-, and ^13^C-NMR spectral analyses.

The proposed in silico studies were represented by druggability predictions, and theoretical quantum and thermodynamical calculations of their antioxidant potential. According to the druggability predictions, all compounds had good druggability properties, with no violations of Lipinski’s rule of five, and poor-to-moderate solubility despite their chemical state as salts. Based on the conducted theoretical calculations, the studied substitutions on the coumarin heterocycle in compounds **4a**–**g** were predicted to have negligible influence on the reactivity of catechol and hydrazone sites. Compound **4f** emerged as the most promising antioxidant molecule, having the highest HOMO–LUMO gaps and some of the lowest BDE values.

The proposed in vitro studies were represented by antiradical, electron transfer capacity, and ferrous ions chelation assays for the antioxidant activity. In accordance with the theoretical calculations, following the antioxidant assays, all compounds presented important activity, with compounds **4f** and **4g** highlighted as the most potent molecules.

Based on the observations made in this study, compounds **4a**–**g** have been defined as promising antioxidants based on the in silico and in vitro assays conducted in this study. The present study represents a starting point for further exploration of the antioxidant activity, using animal or cell models, or the possibility of studying a synergistic effect between the antioxidant activity of these compounds and any possible antimicrobial or anticancer activity that these compounds may possess. Compounds **4f** and **4g** serve as HIT/Leader molecules for further research.

## Figures and Tables

**Table 1:** The computed in silico physicochemical descriptors for compounds **4a**–**g**.

Compound	MW (g/mol)	No. RB	No. HBA	No. HBD	TPSA (Å^2^)	MLogP	ESOL (µg/mL)
**4a**	394.42	4	7	3	140.77	−1.81	2.14
**4b**	424.45	5	8	3	150.00	−2.11	1.98
**4c**	438.48	6	8	3	150.00	−1.89	1.19
**4d**	473.32	4	7	3	140.77	−1.21	0.31
**4e**	444.48	4	7	3	140.77	−1.11	0.18
**4f**	410.42	4	8	4	161.00	−2.33	3.07
**4g**	410.42	4	8	4	161.00	−2.33	3.07

**Table 2:** The levels of energy of the frontier molecular orbitals for compounds **4a**–**g** (eV).

Compound	Vacuum			Nonpolar			Water		
	HOMO	LUMO	gap	HOMO	LUMO	gap	HOMO	LUMO	gap
**4a**	−5.14	−2.10	3.04	−5.17	−2.03	3.14	−5.34	−2.09	3.25
**4b**	−5.14	−2.10	3.04	−5.18	−2.04	3.14	−5.34	−2.13	3.21
**4c**	−5.14	−2.09	3.05	−5.17	−2.04	3.13	−5.33	−2.16	3.17
**4d**	−5.21	−2.33	2.88	−5.22	−2.22	3.00	−5.36	−2.27	3.09
**4e**	−5.12	−2.18	2.94	−5.15	−2.12	3.03	−5.31	−2.21	3.10
**4f**	−5.17	−2.12	3.05	−5.18	−2.05	3.13	−5.34	−2.12	3.22
**4g**	−5.09	−1.96	3.13	−5.12	−1.90	3.22	−5.31	−1.98	3.33

**Table 3:** The BDE for the sites which can release hydrogen atoms from compounds **4a**–**g** in vacuum, nonpolar environment, and water (kcal/mol).

Compound	Vacuum					Nonpolar					Water				
	O-H_1_	O-H_2_	N-H_3_	O-H_4_	O-H_5_	O-H_1_	O-H_2_	N-H_3_	O-H_4_	O-H_5_	O-H_1_	O-H_2_	N-H_3_	O-H_4_	O-H_5_
**4a**	66.18	68.72	70.54	-	-	65.40	68.24	70.31	-	-	69.22	72.37	72.05	-	-
**4b**	66.26	68.77	70.63	-	-	65.52	68.23	70.31	-	-	69.36	72.46	72.28	-	-
**4c**	66.24	68.76	70.37	-	-	65.55	68.27	70.29	-	-	69.29	72.31	71.95	-	-
**4d**	66.45	68.84	70.49	-	-	65.66	68.26	70.36	-	-	69.43	72.43	72.17	-	-
**4e**	66.29	68.79	70.19	-	-	65.59	68.23	69.90	-	-	69.38	72.34	71.57	-	-
**4f**	66.31	68.78	70.62	78.63	-	65.52	68.15	70.24	77.14	-	69.42	72.52	72.18	80.63	-
**4g**	66.09	68.62	70.12	-	76.70	65.40	68.17	69.63	-	75.70	69.30	72.44	71.24	-	79.10

**Table 4:** The IC_50_ values for compounds **4a**–**g** against the DPPH^•^ (µM) and ABTS^•+^ (µM).

Compound	DPPH^•^	ABTS^•+^
**4a**	29.90	12.62
**4b**	29.54	11.77
**4c**	29.88	11.60
**4d**	33.49	14.04
**4e**	28.60	10.88
**4f**	24.57	8.38
**4g**	23.84	7.06
**Ascorbic acid**	50.17	-
**Trolox**	36.69	16.57

**Table 5:** Results of the electron transfer capacity assays for compounds **4a**–**g**, expressed as molar Eq activity of ascorbic acid and/or Trolox (2 mM stock solutions taken in equimolar amounts).

Compound	TAC	RP		FRAP	CUPRAC
	Eq Ascorbic Acid	Eq Ascorbic Acid	Eq Trolox	Eq Trolox	Eq Trolox
**4a**	1.53	1.96	1.52	1.37	3.09
**4b**	1.53	1.84	1.43	1.40	3.16
**4c**	1.47	1.83	1.42	1.38	3.21
**4d**	1.61	1.08	0.84	1.14	2.27
**4e**	1.33	1.71	1.33	1.20	2.99
**4f**	2.20	2.54	1.98	1.45	3.63
**4g**	2.18	2.59	2.02	1.42	3.60

## Data Availability

The data presented in this study are available in this article.

## Supplementary Materials

The following supporting information can be downloaded at: https://www.mdpi.com/article/10.3390/antiox14060636/s1, Figures S1–S21: The IR spectra for compounds **2a–4g**; Figures S22–S42: The MS spectra for compounds **2a–4g**; Figures S43-S49: The ^1^H-NMR spectra for compounds **4a**–**g**; Figures S50–S56: The ^13^C-NMR spectra for compounds **4a**–**g**; Figures S57–S63: The spin density maps for compounds **4a**–**g**; Figures S64–S70: The depictions of HOMO and LUMO and the electrostatic potential maps for compounds **4a**–**g**.
