# Supplementary material for: Novel 3,4-Dihydroxyphenyl-Thiazole-Coumarin Hybrid Compounds: Synthesis, In Silico and In Vitro Evaluation of Their Antioxidant Activity
Source: Antioxidants (Basel). 2025 May 26;14(6):636. doi: 10.3390/antiox14060636 (PMC12189554; doi:10.3390/antiox14060636)
Supplement: Supplementary file 1 [file antioxidants-14-00636-s001.zip › antioxidants-3624949-supplementary.pdf]

antioxidants

MDPI

Article

Supplementary Material: Novel 3,4-Dihydroxyphenyl-Thiazole-Coumarin Hybrid Compounds: Synthesis, In Silico and In Vitro Evaluation of the Antioxidant Activity

Daniel Ungureanu, Gabriel Marc, Brîndușa Tiperciuc, Cristina Moldovan, Ioana Ionuț, Anca Stana, Ilioara Oniga, Laurian Vlase, Adrian Pîrnău, Ovidiu Oniga

1. Figures

1.1. IR Spectra

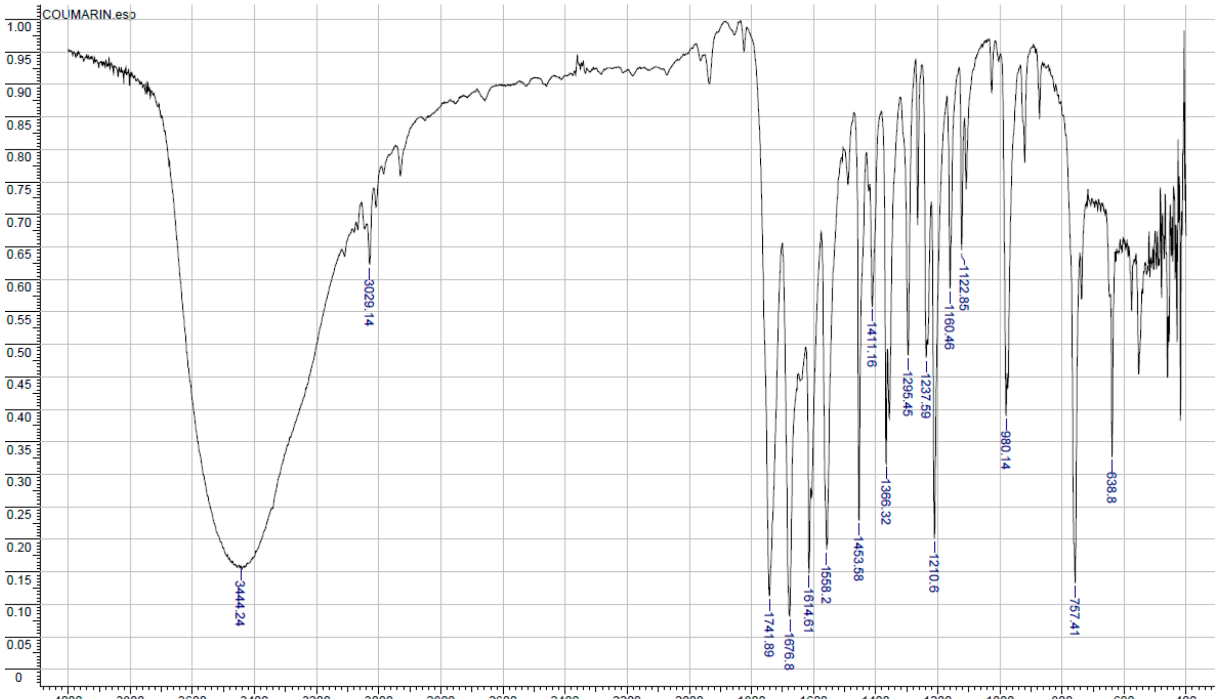

Figure S1. The IR spectrum for the compound **2a**.

 https://doi.org/10.3390/antiox14060636

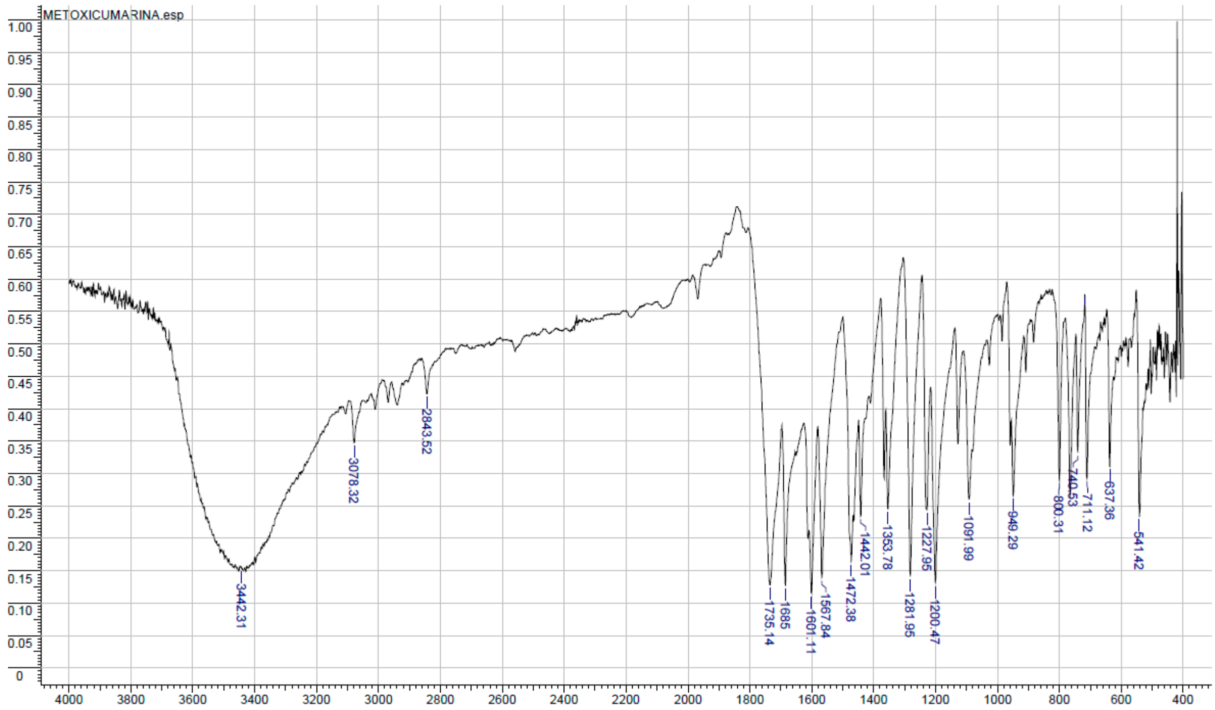

Figure S2. The IR spectrum for the compound **2b**.

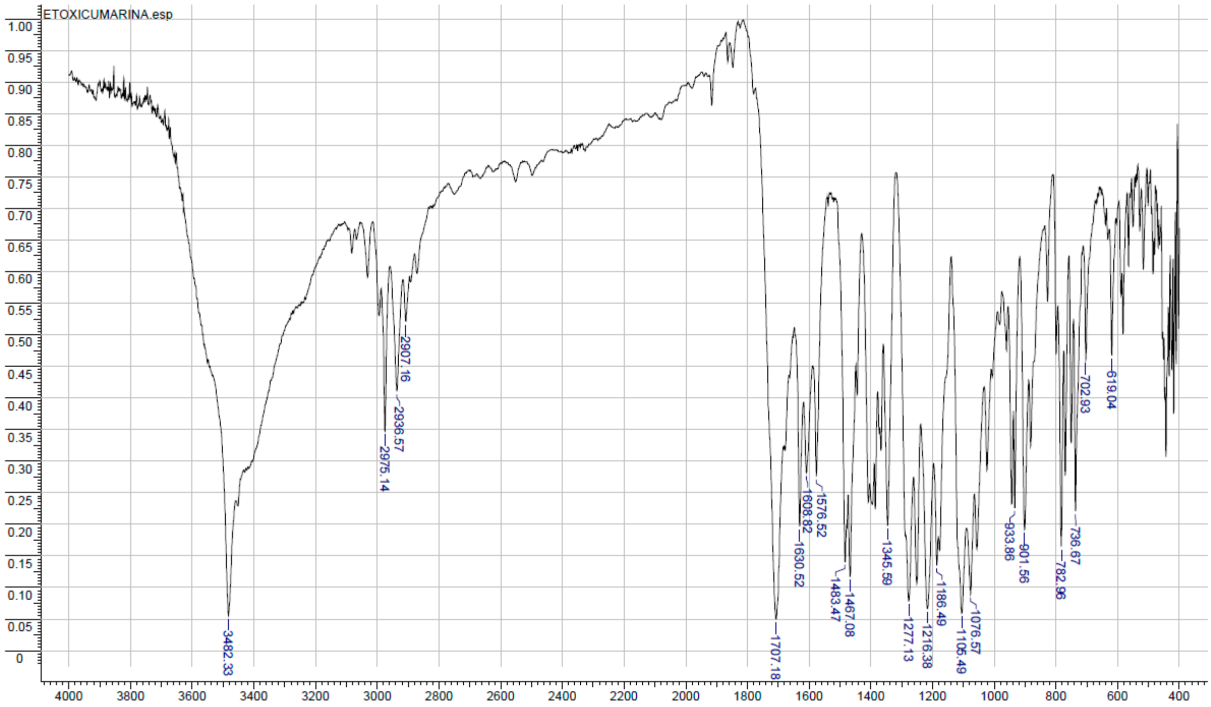

Figure S3. The IR spectrum for the compound **2c**.

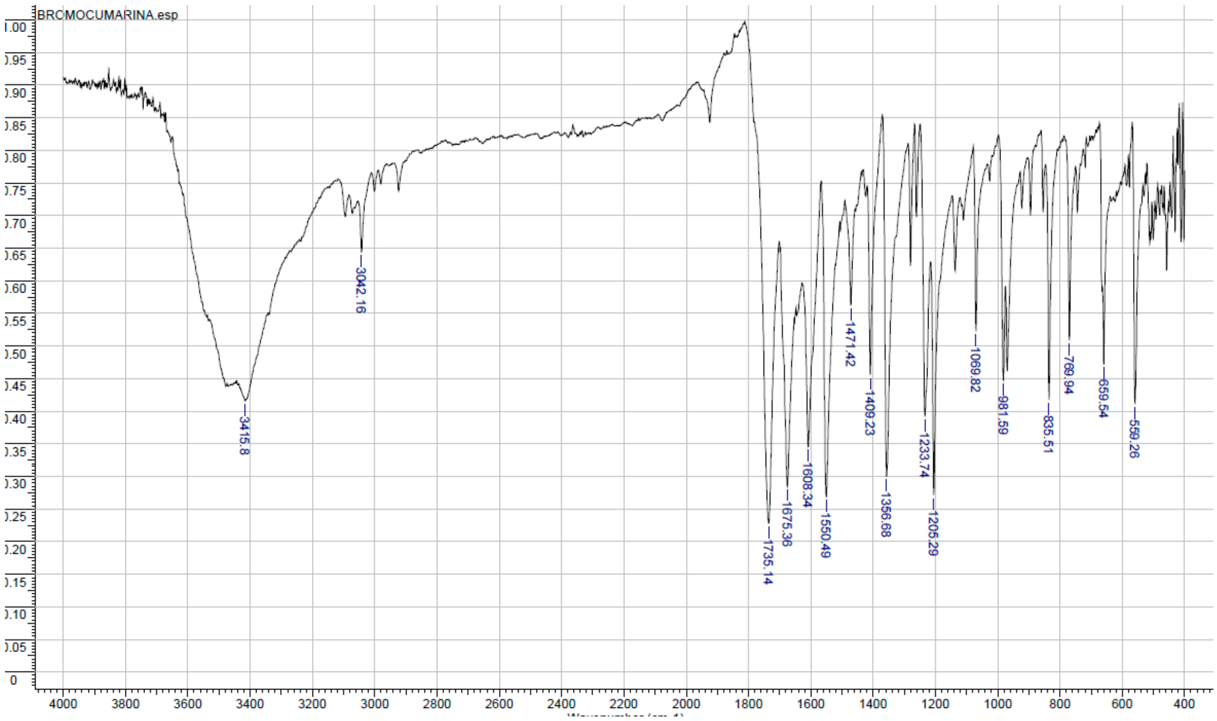

Figure S4. The IR spectrum for the compound **2d**.

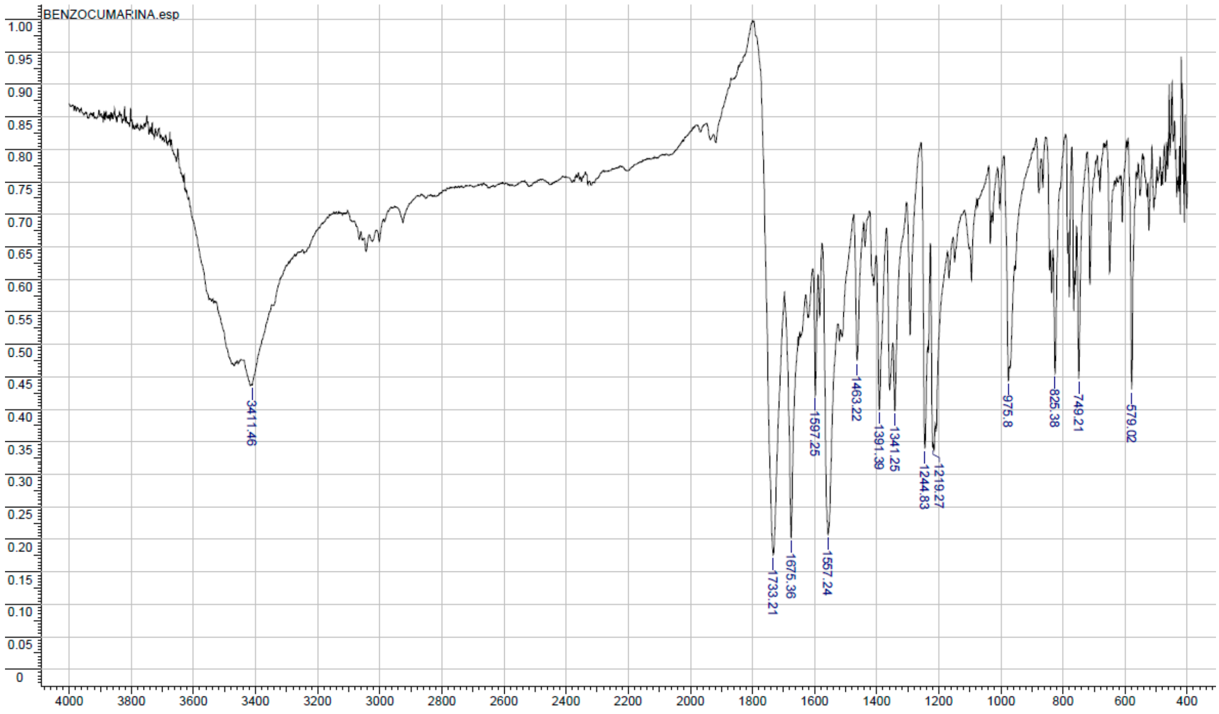

Figure S5. The IR spectrum for the compound **2e**.

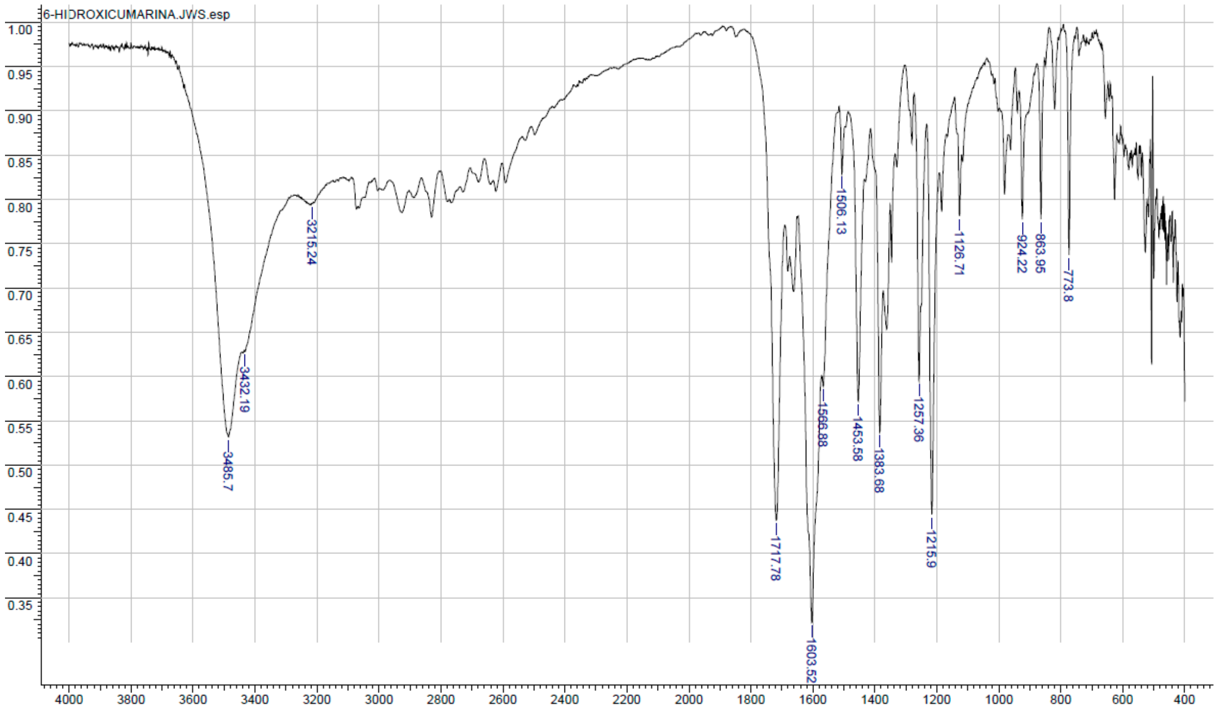

Figure S6. The IR spectrum for the compound **2f**.

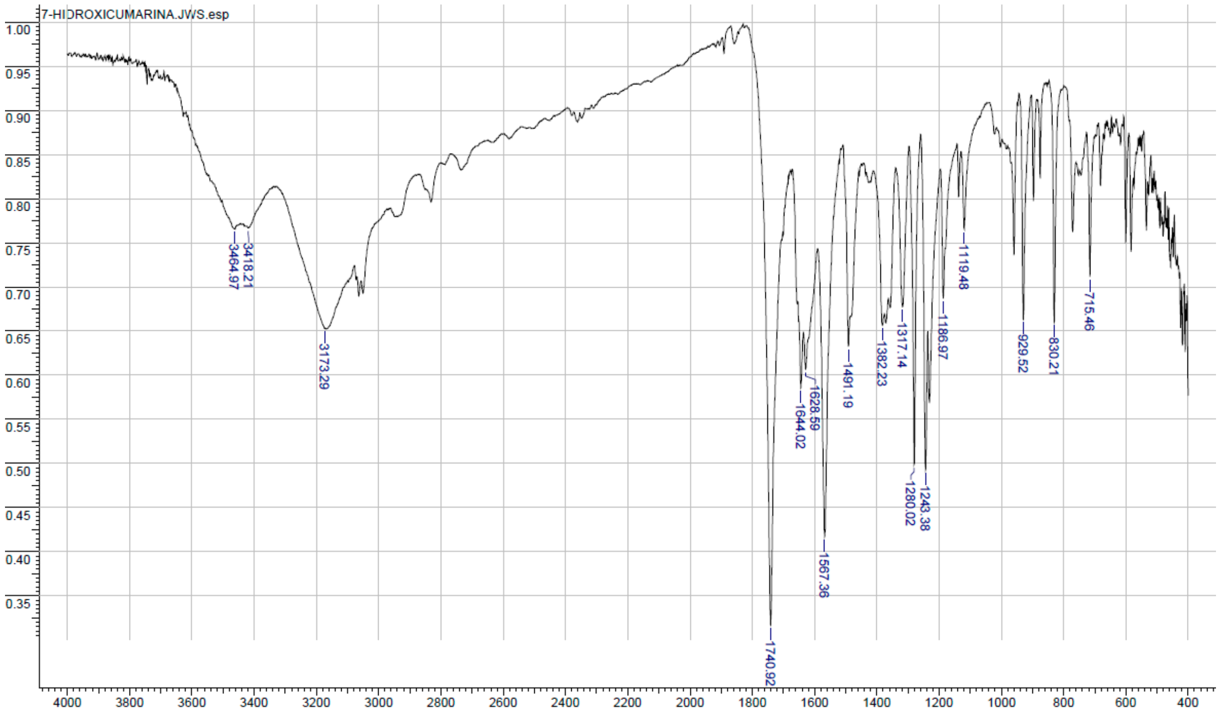

Figure S7. The IR spectrum for the compound **2g**.

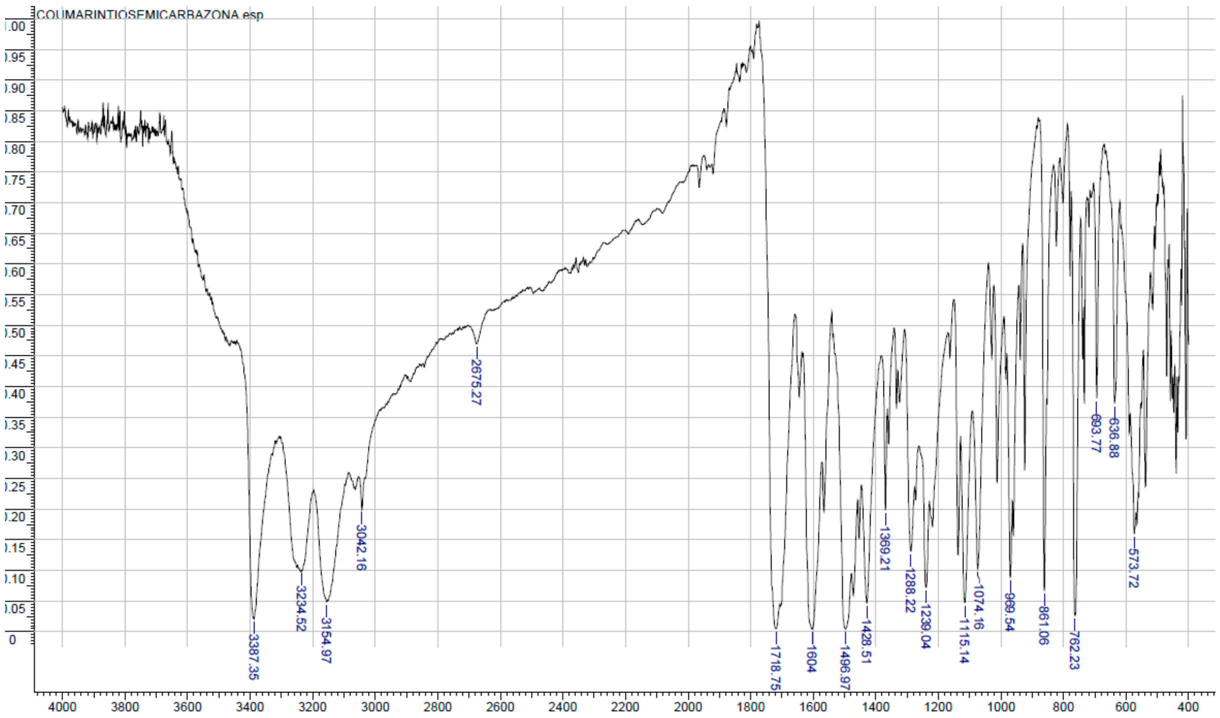

Figure S8. The IR spectrum for the compound **3a**.

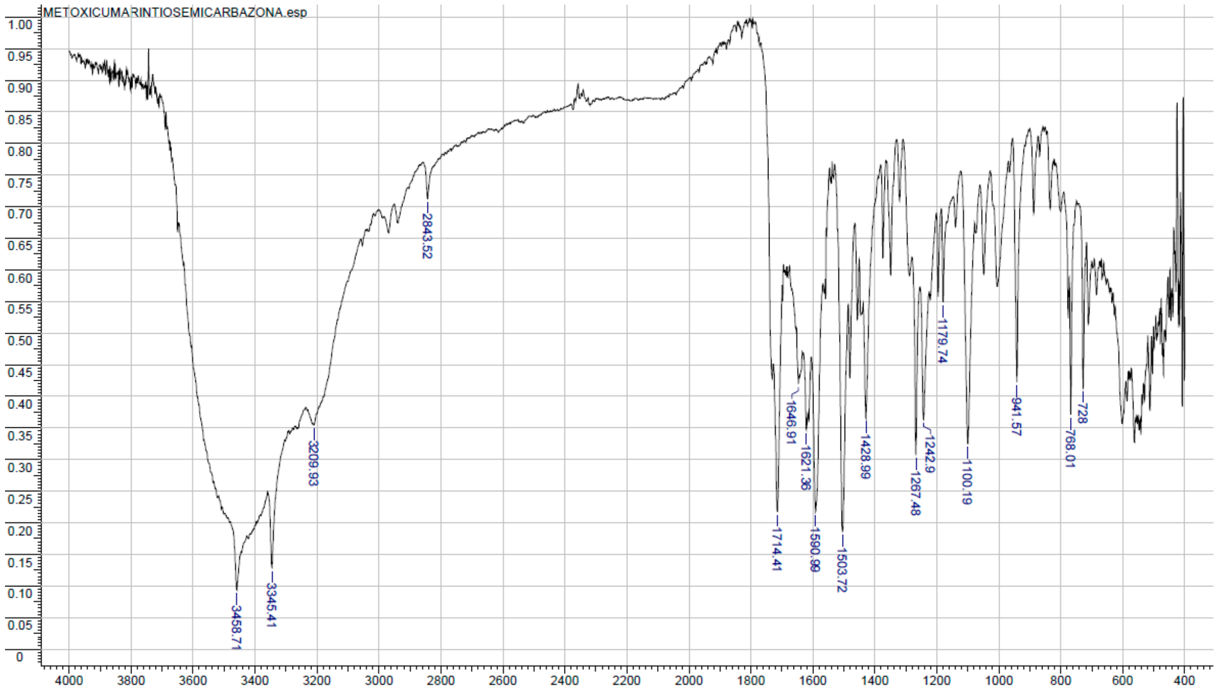

Figure S9. The IR spectrum for the compound **3b**.

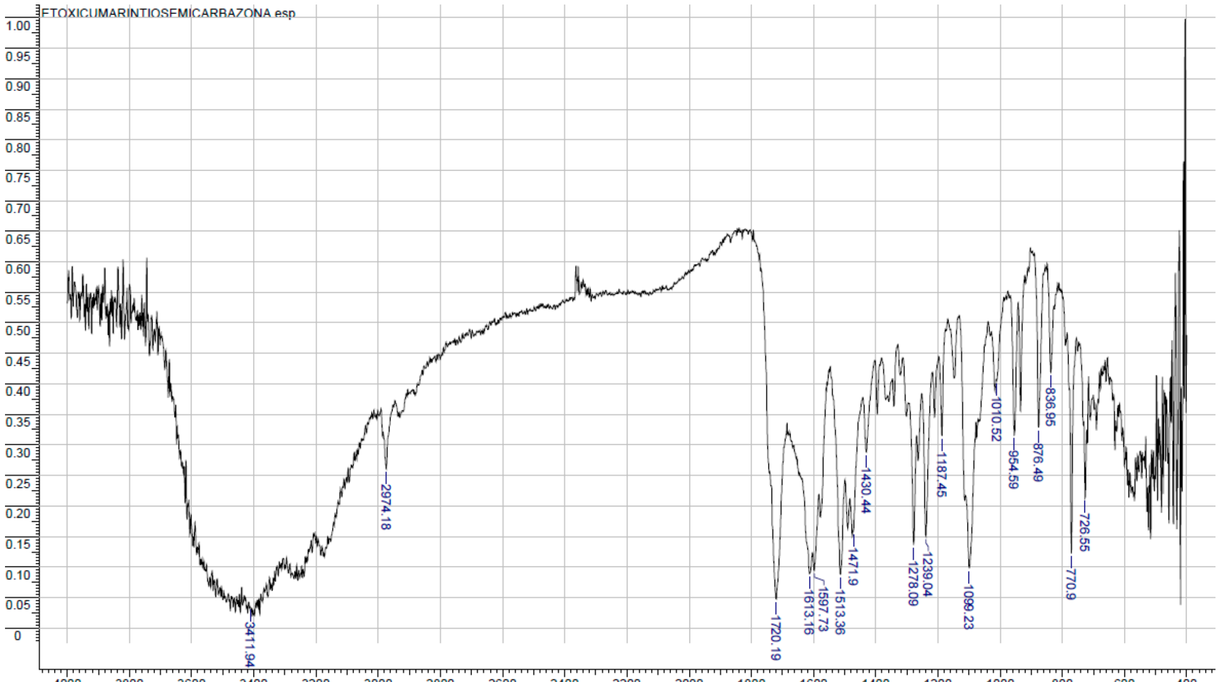

Figure S10. The IR spectrum for the compound **3c**.

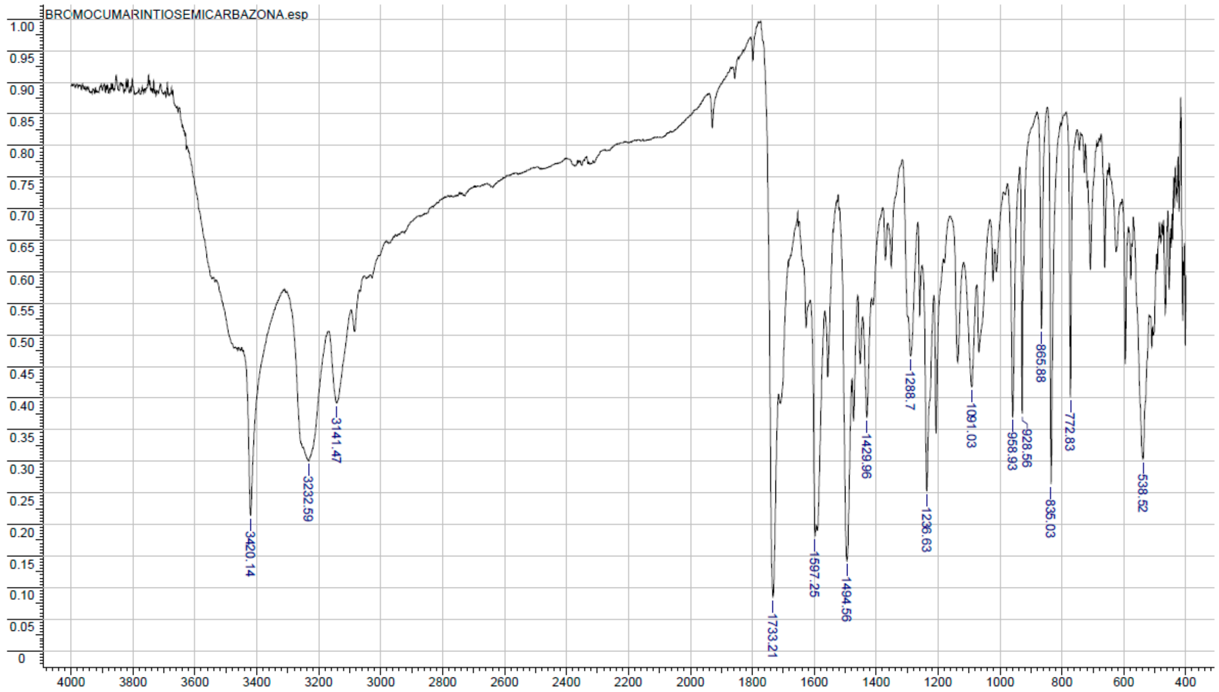

Figure S11. The IR spectrum for the compound **3d**.

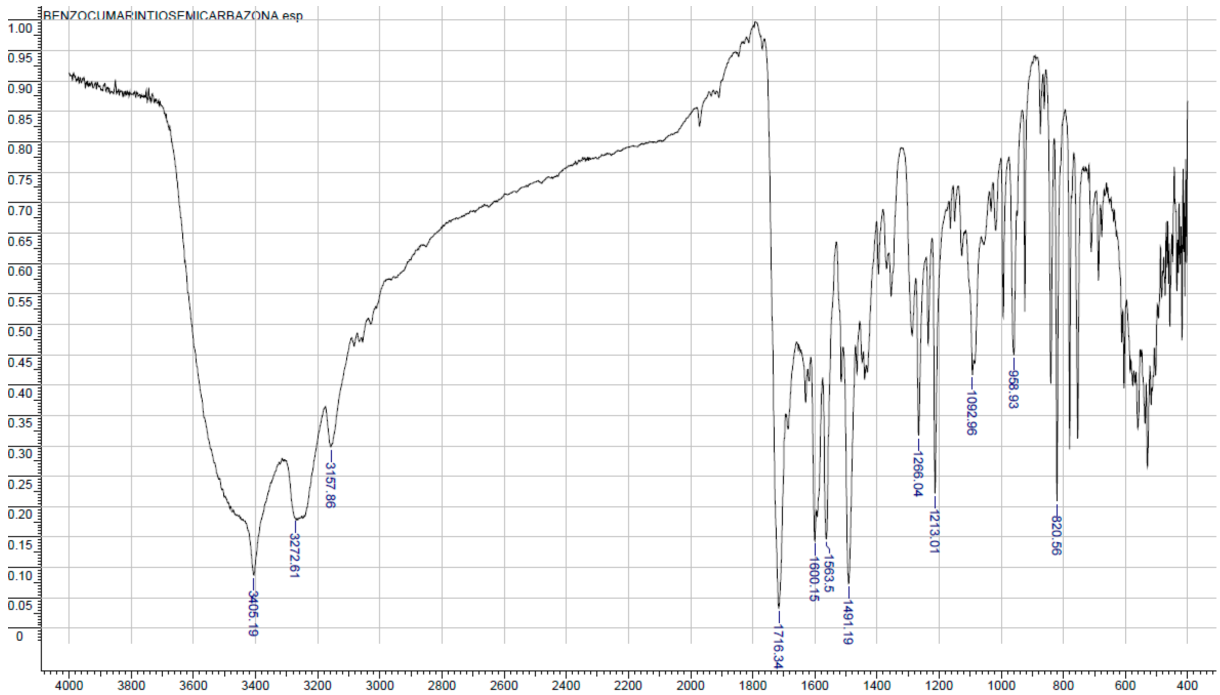

Figure S12. The IR spectrum for the compound **3e**.

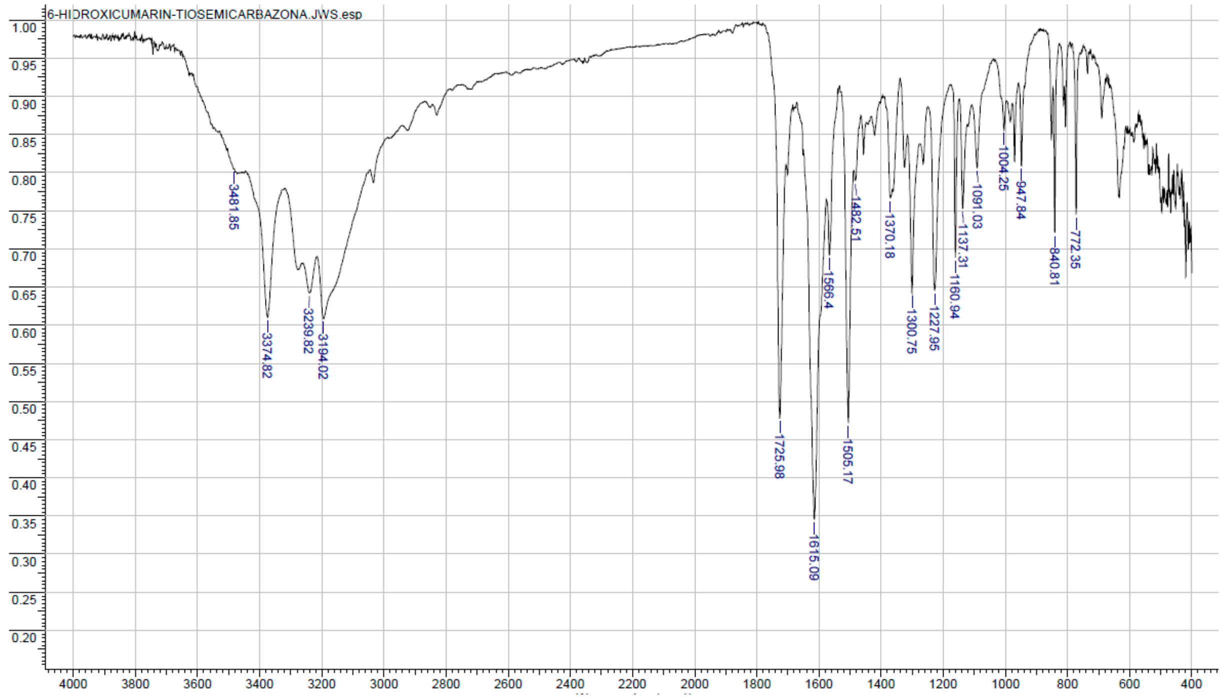

Figure S13. The IR spectrum for the compound **3f**.

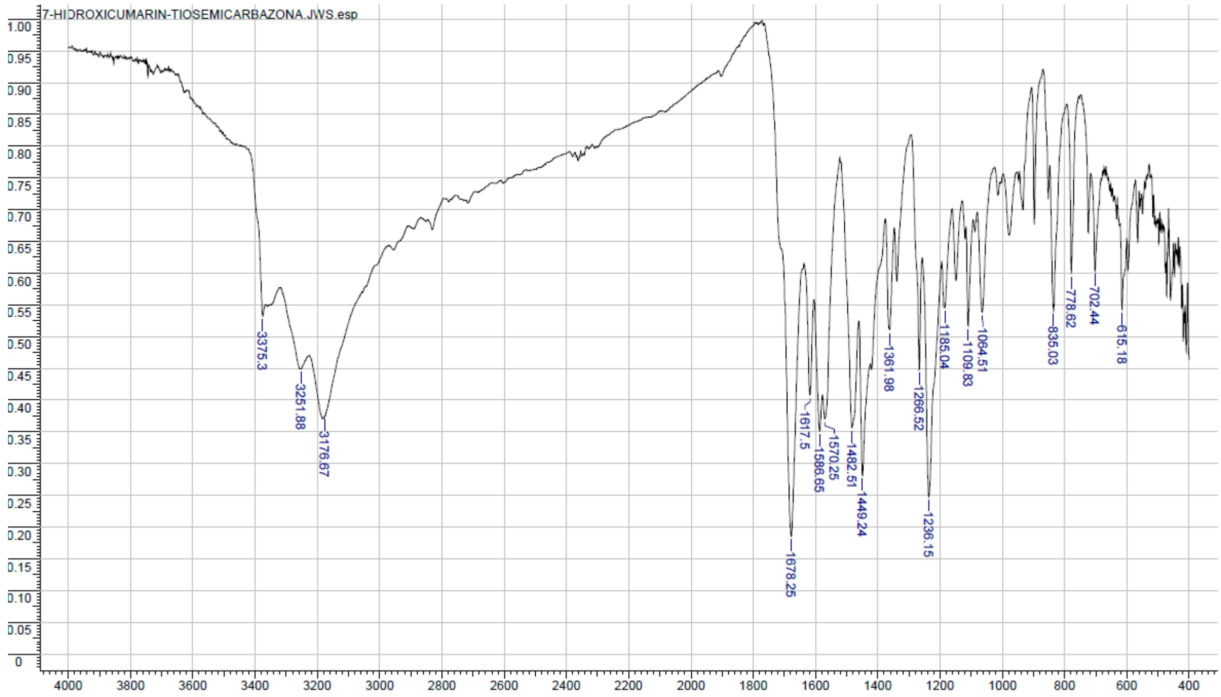

Figure S14. The IR spectrum for the compound **3g**.

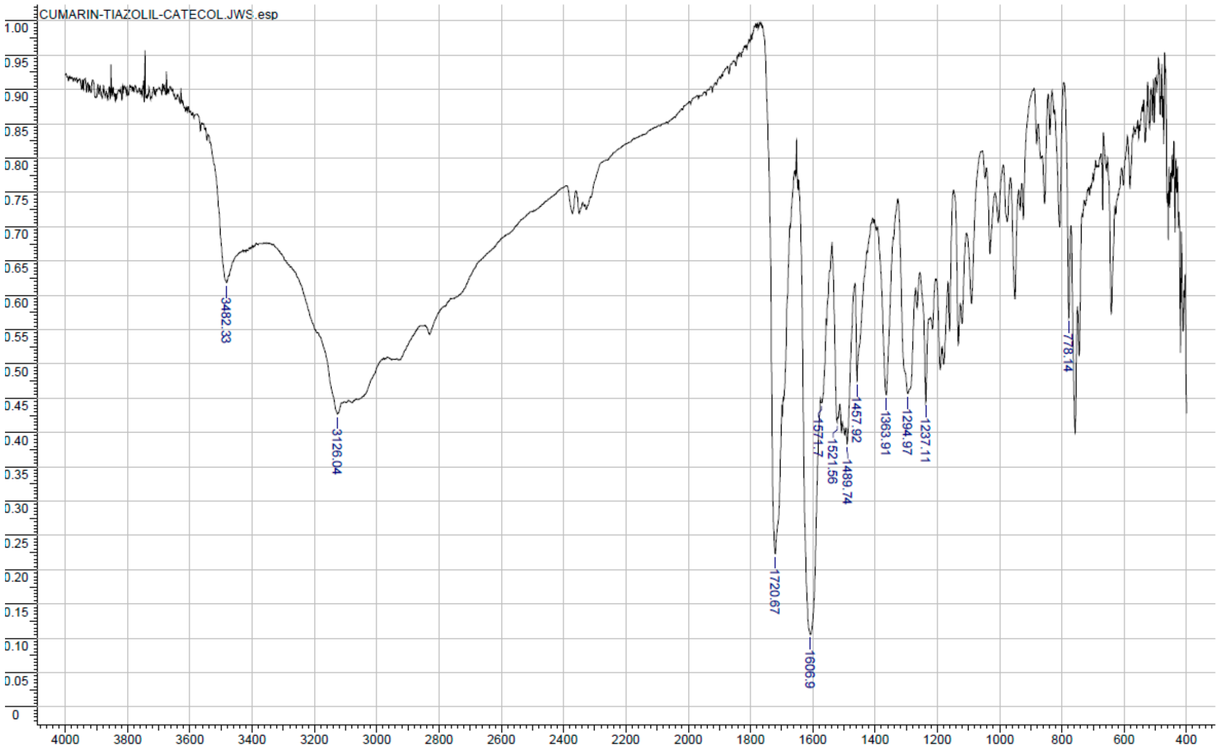

Figure S15. The IR spectrum for the compound **4a**.

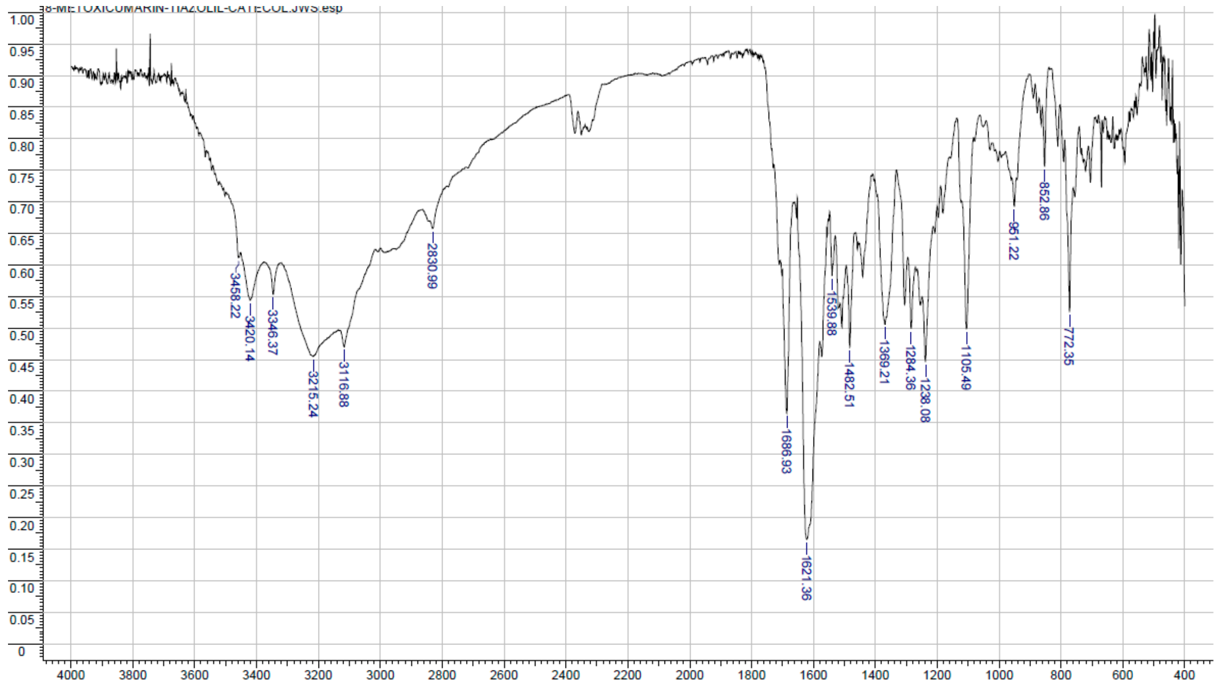

Figure S16. The IR spectrum for the compound **4b**.

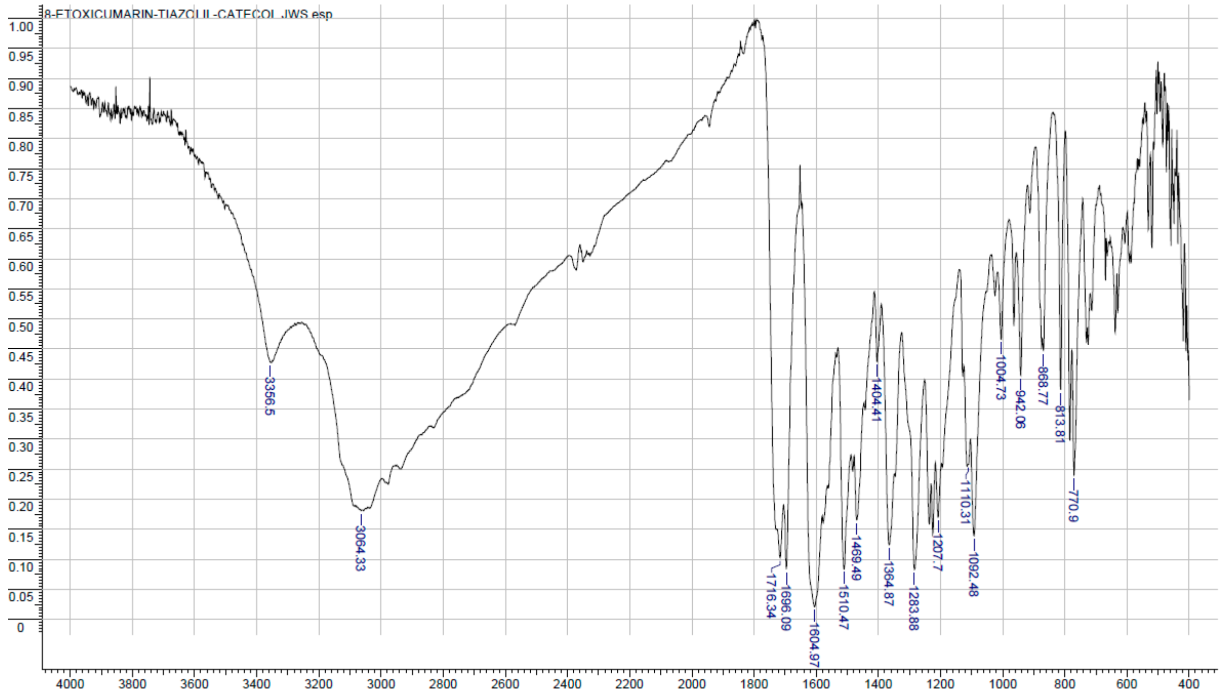

Figure S17. The IR spectrum for the compound **4c**.

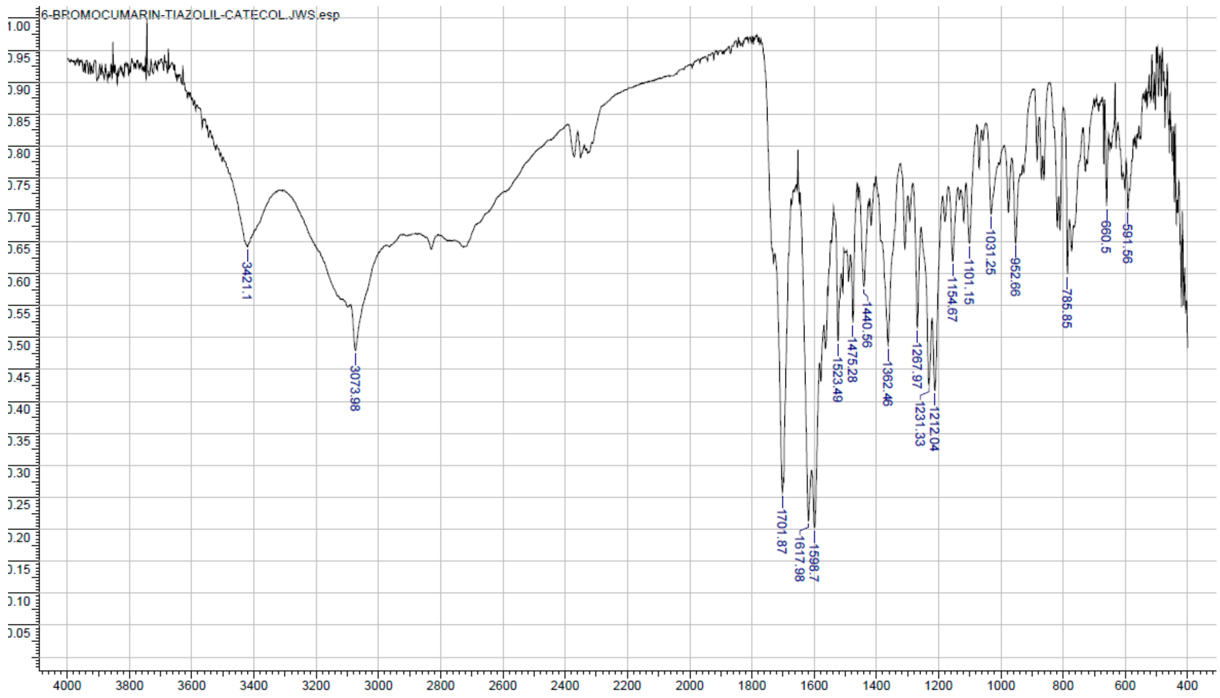

Figure S18. The IR spectrum for the compound **4d**.

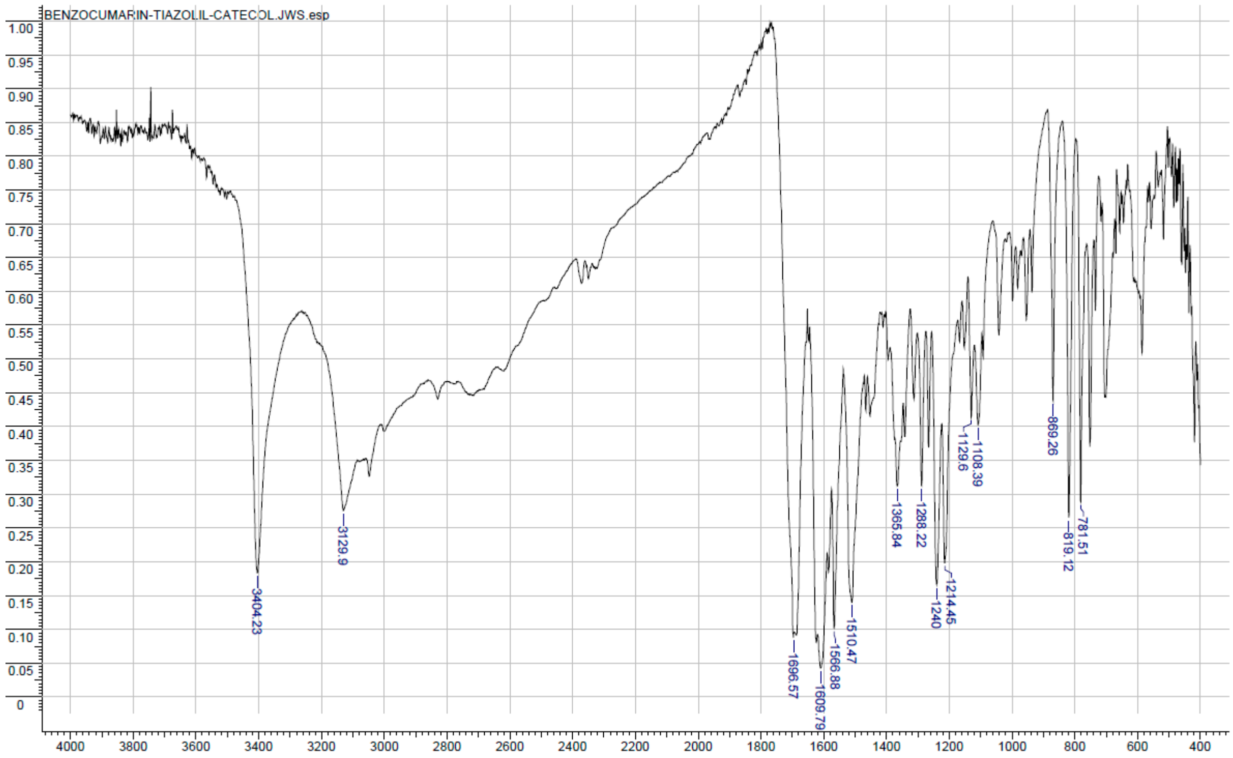

Figure S19. The IR spectrum for the compound **4e**.

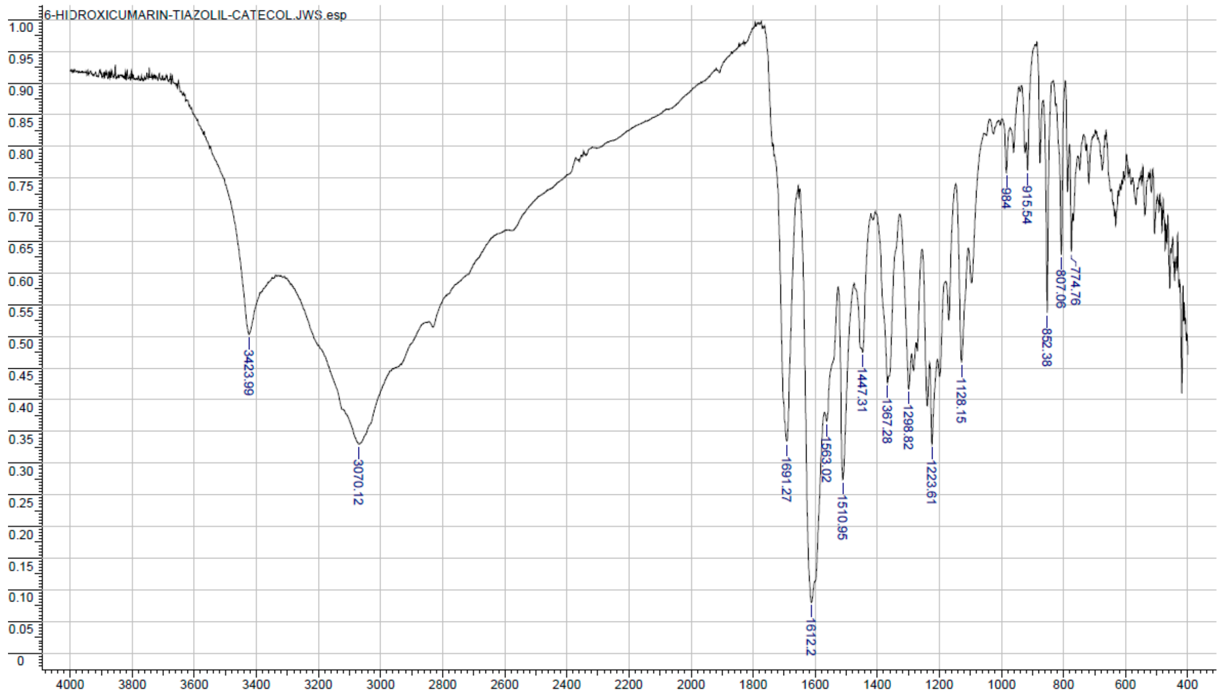

Figure S20. The IR spectrum for the compound **4f**.

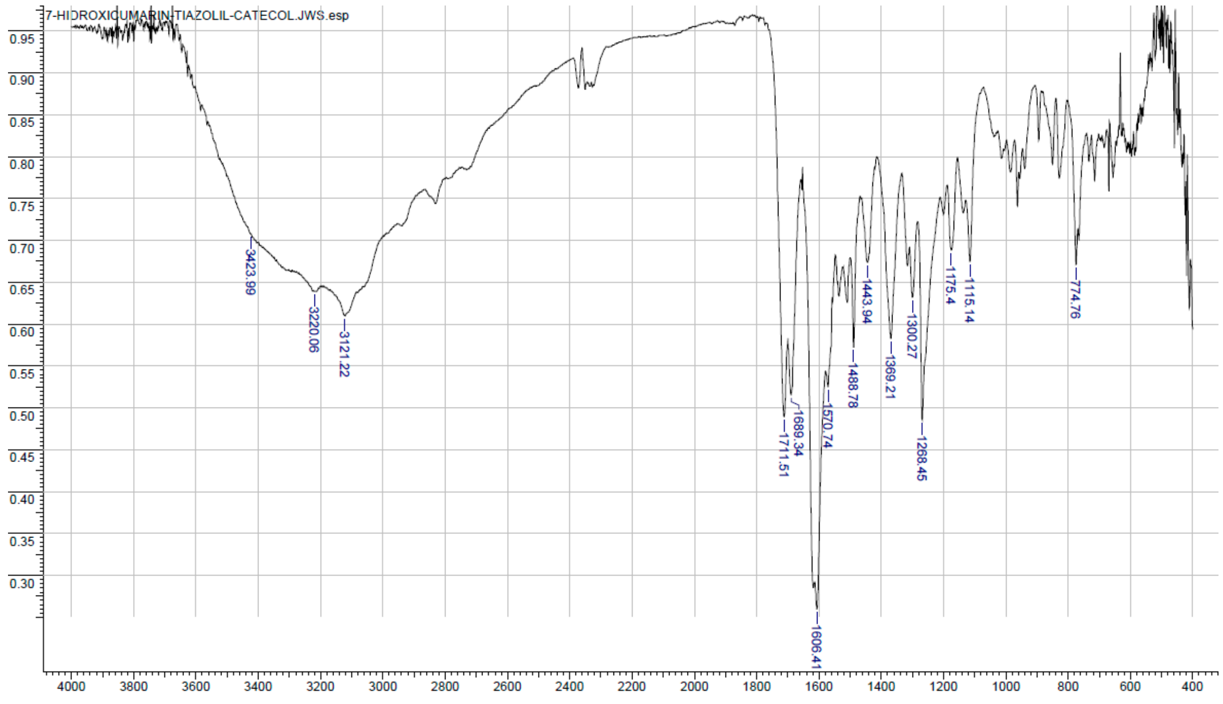

Figure S21. The IR spectrum for the compound **4g**.

1.2. MS Spectra

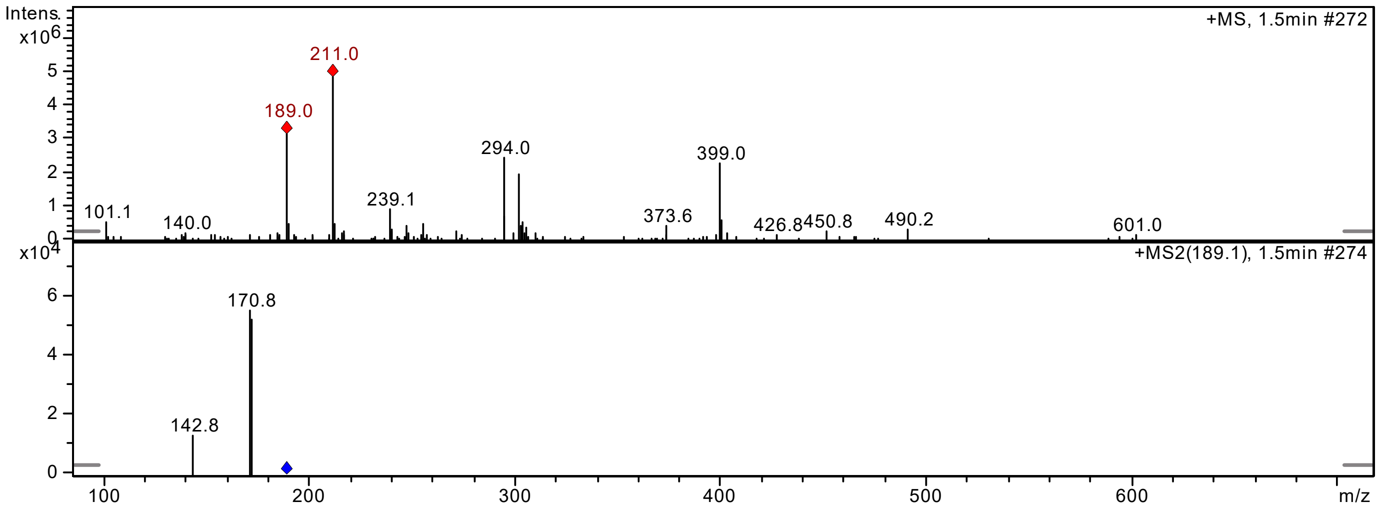

Figure S22. The MS spectrum for the compound **2a**.

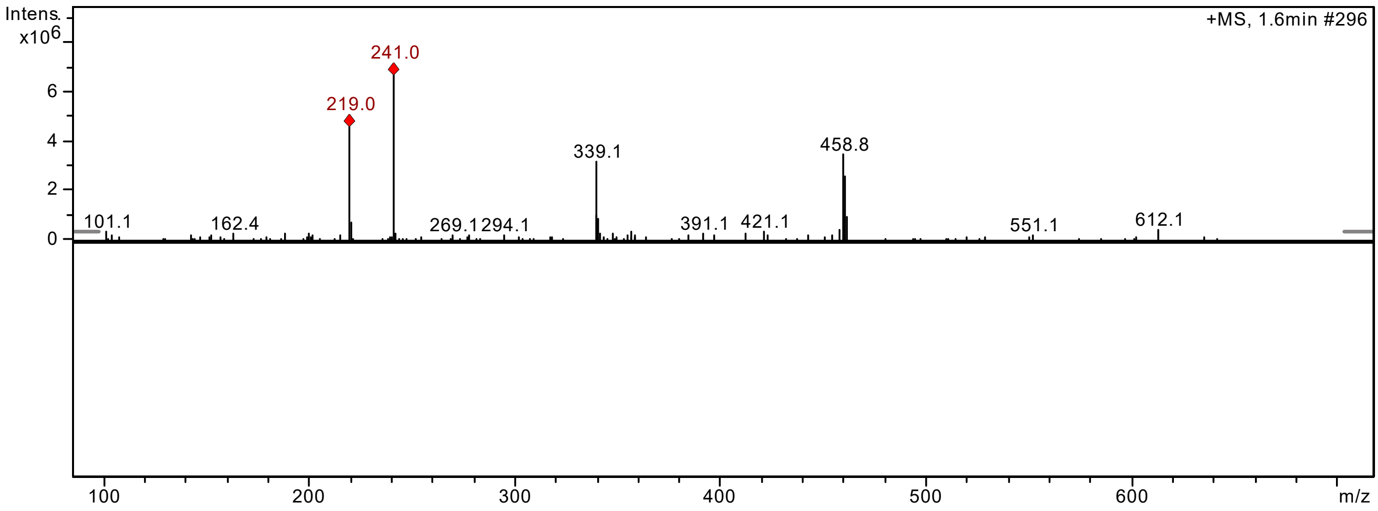

Figure S23. The MS spectrum for the compound **2b**.

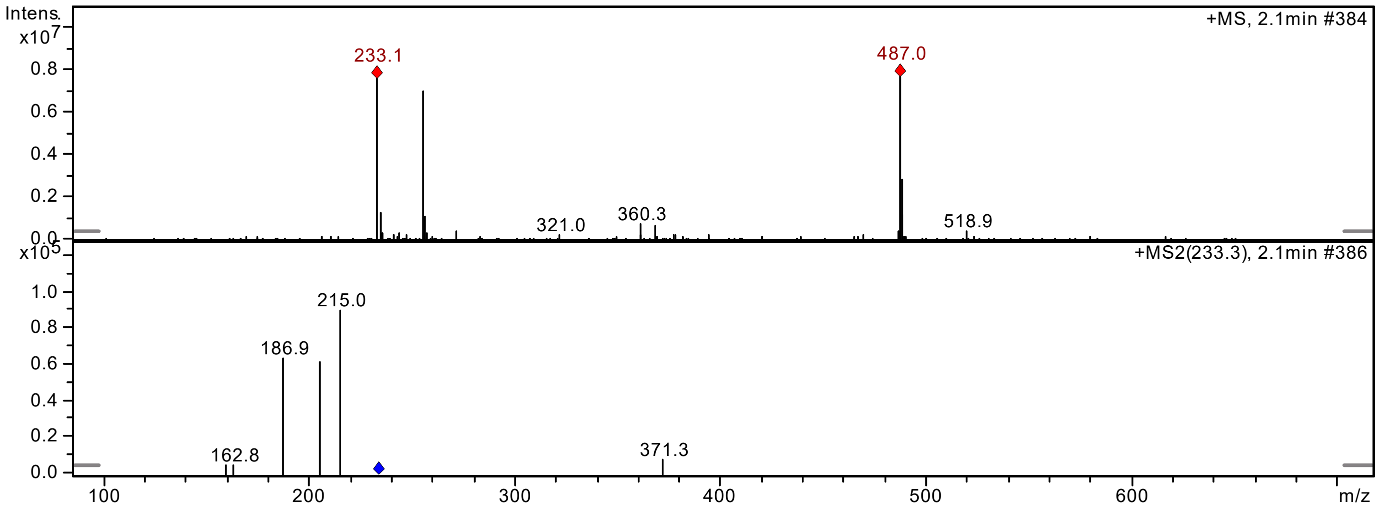

Figure S24. The MS spectrum for the compound **2c**.

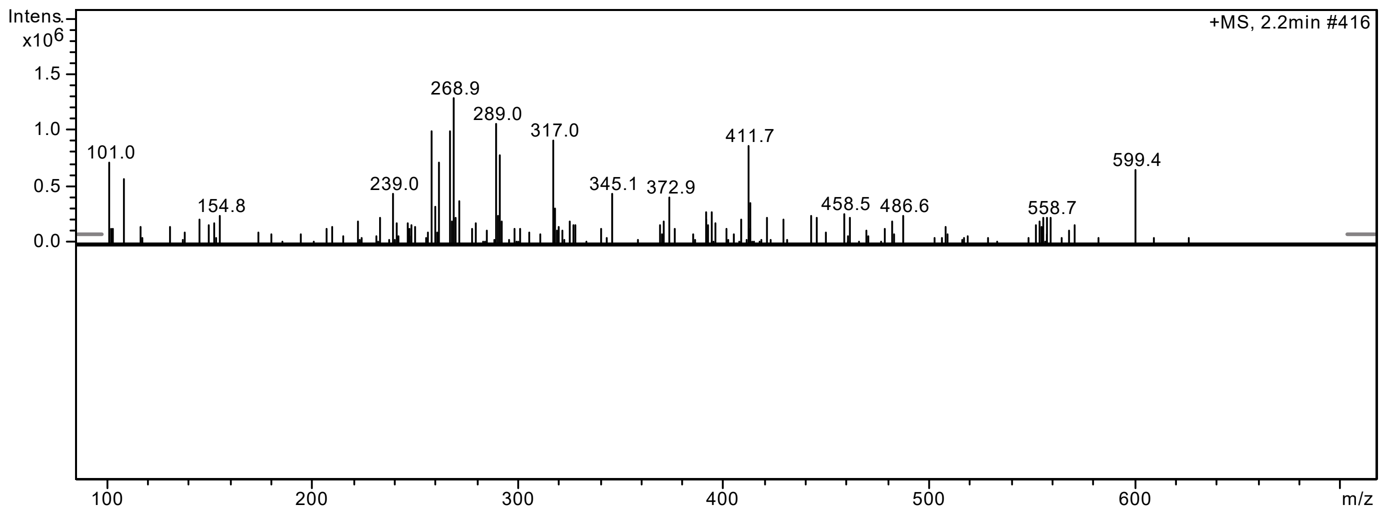

Figure S25. The MS spectrum for the compound **2d**.

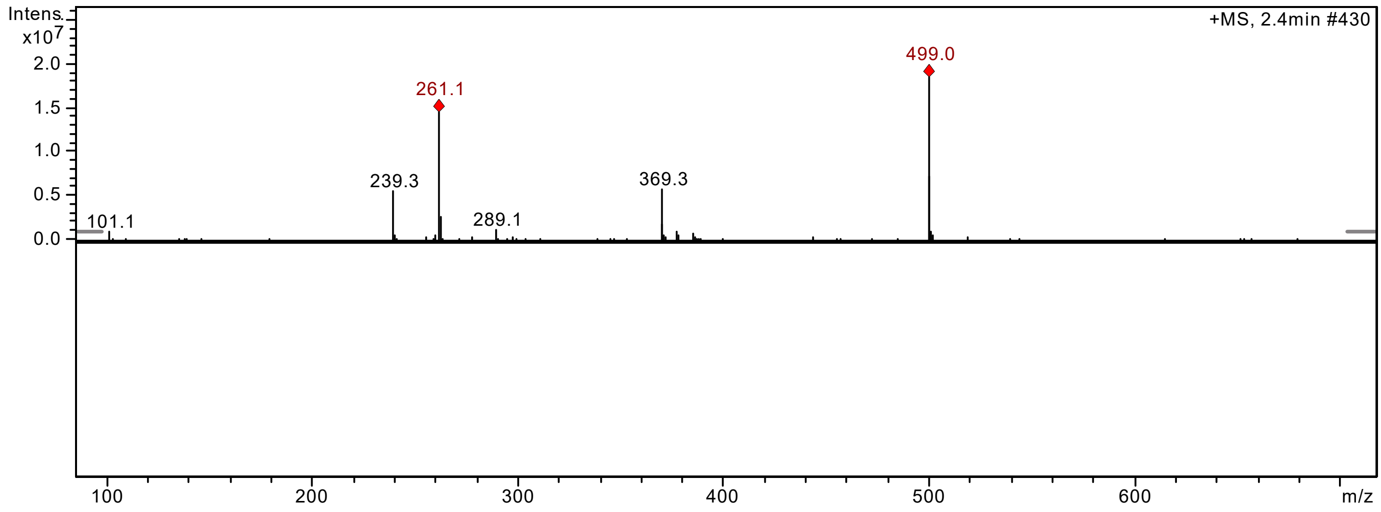

Figure S26. The MS spectrum for the compound **2e**.

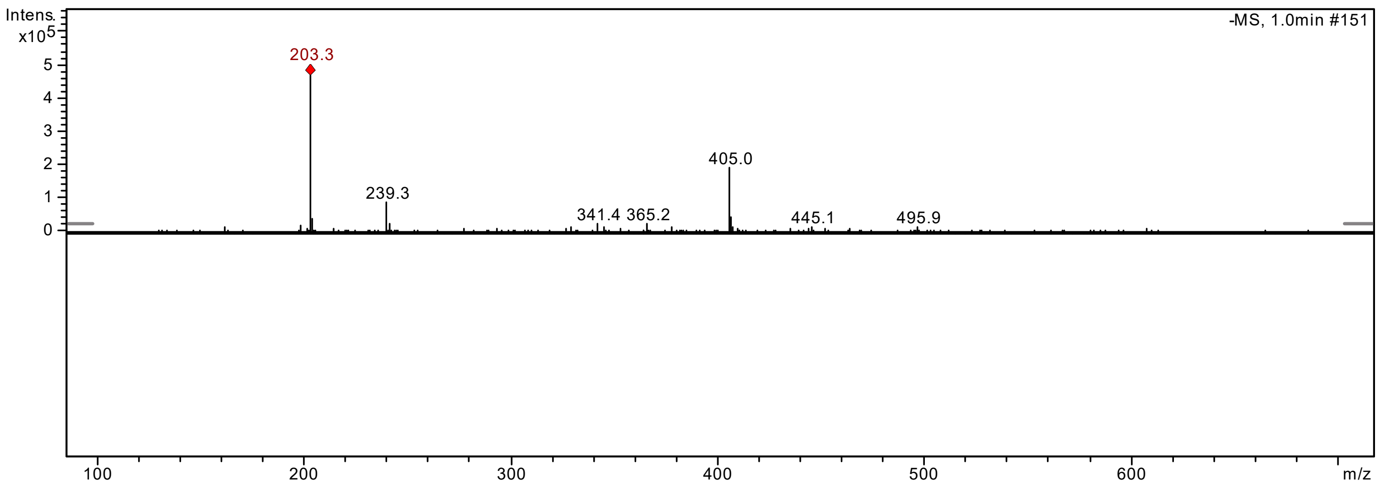

Figure S27. The MS spectrum for the compound **2f**.

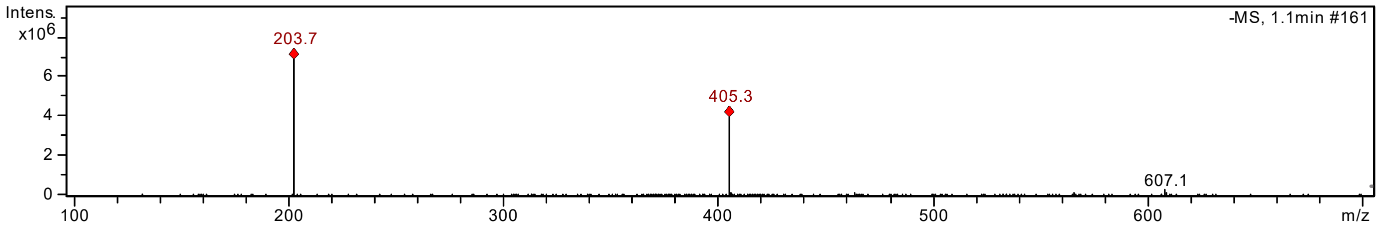

Figure S28. The MS spectrum for the compound **2g**.

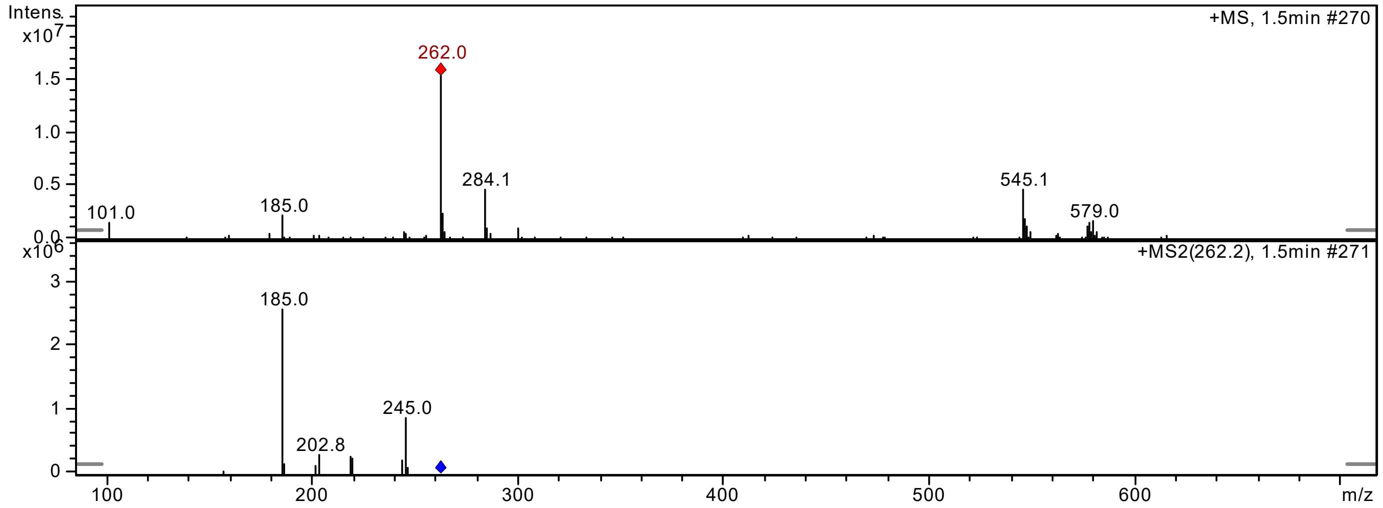

Figure S29. The MS spectrum for the compound **3a**.

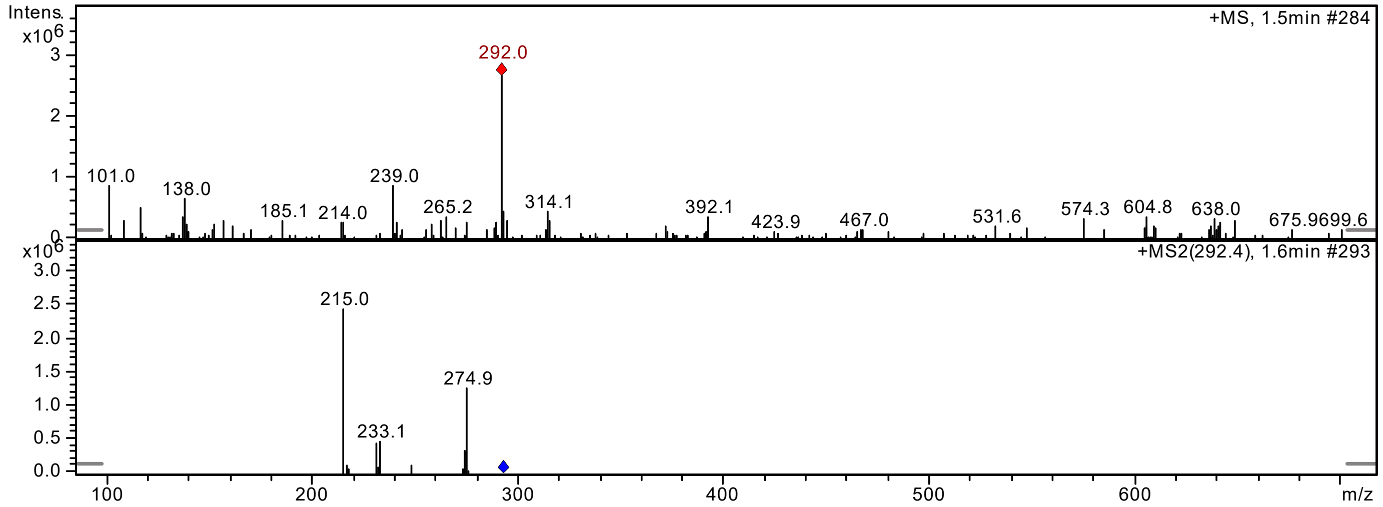

Figure S30. The MS spectrum for the compound **3b**.

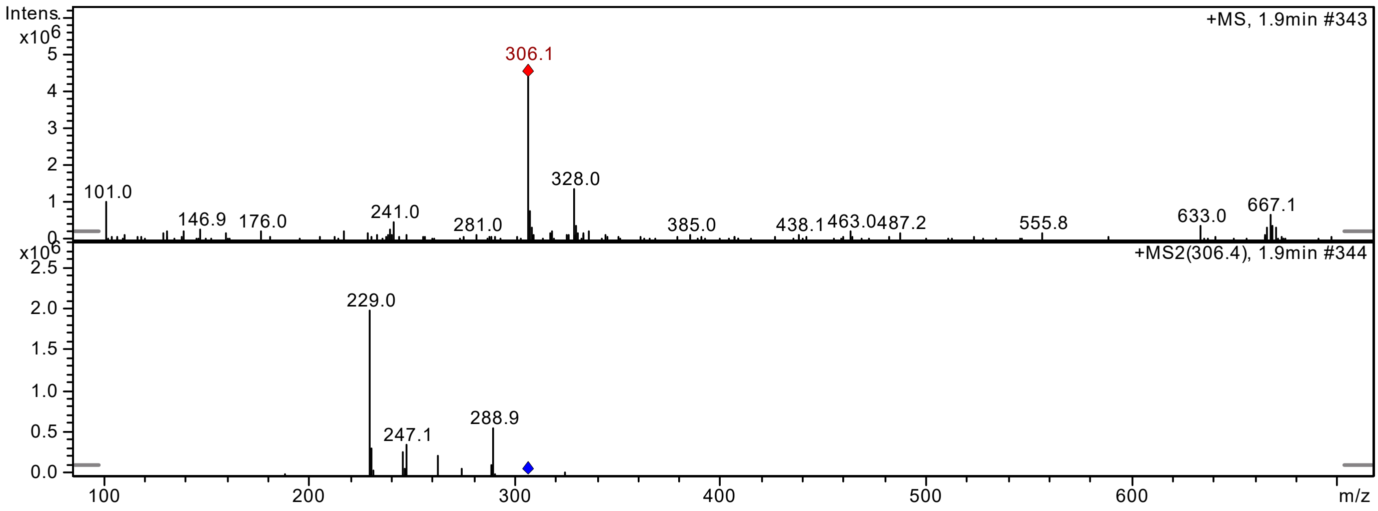

Figure S31. The MS spectrum for the compound **3c**.

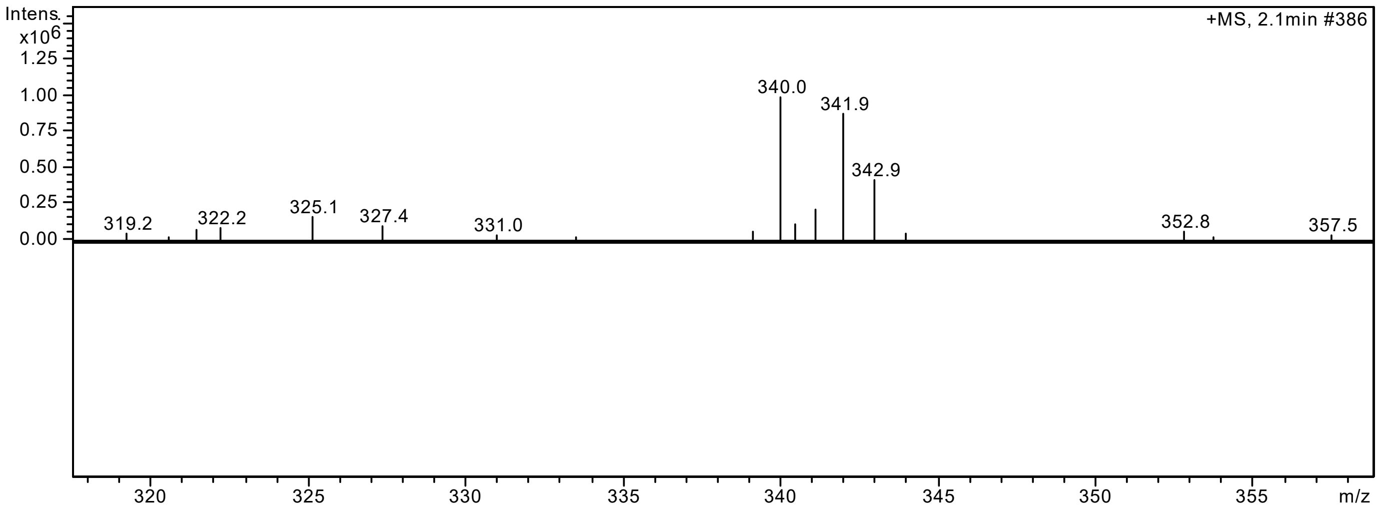

Figure S32. The MS spectrum for the compound **3d**.

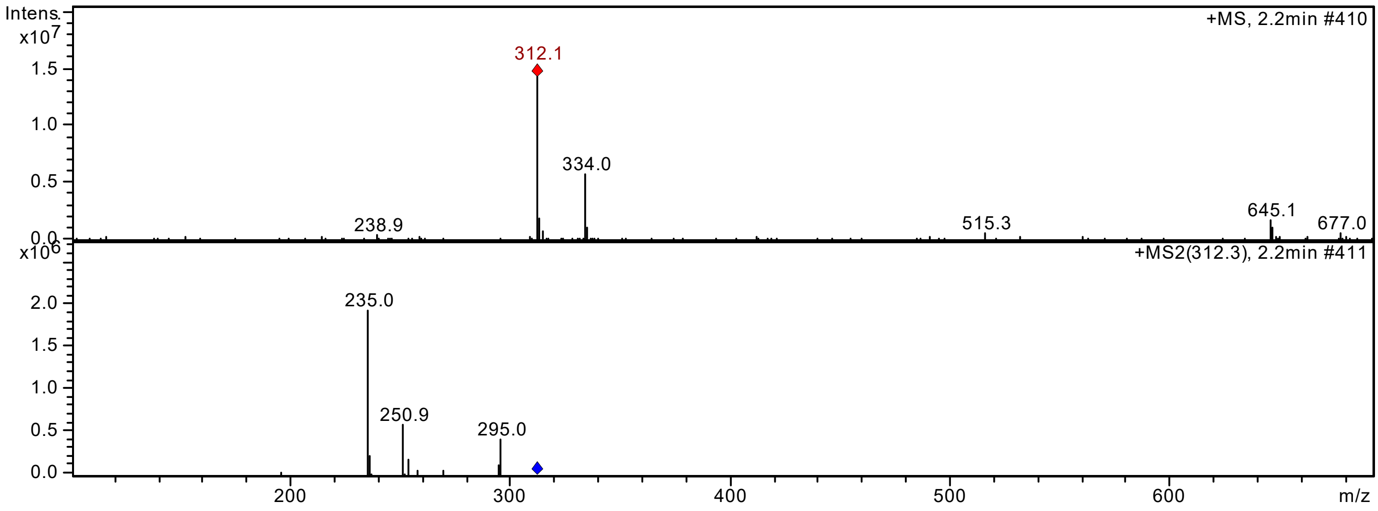

Figure S33. The MS spectrum for the compound **3e**.

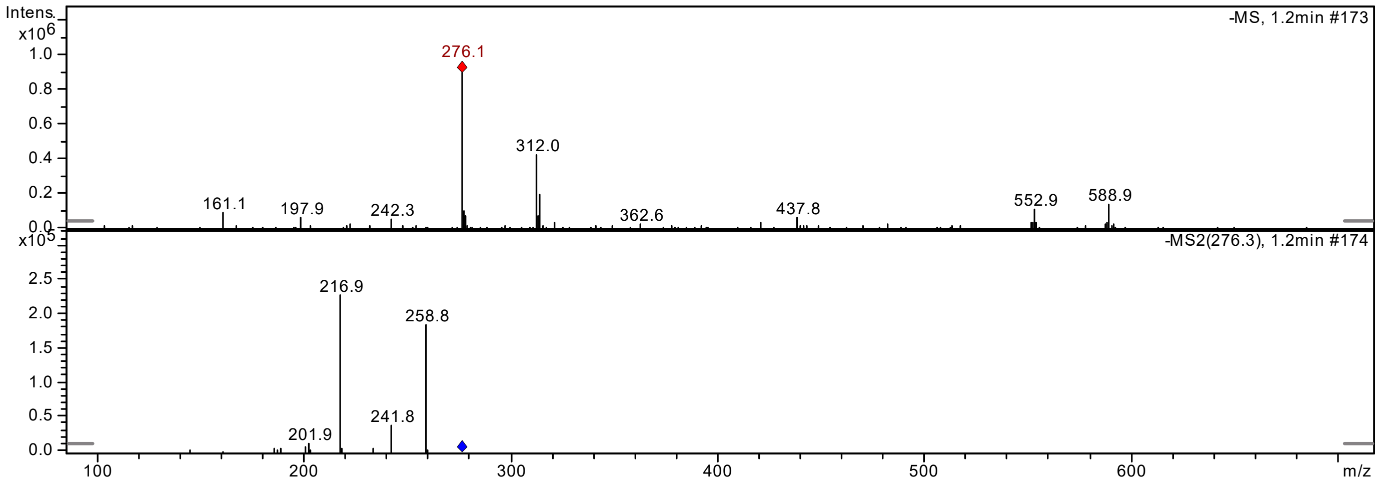

Figure S34. The MS spectrum for the compound **3f**.

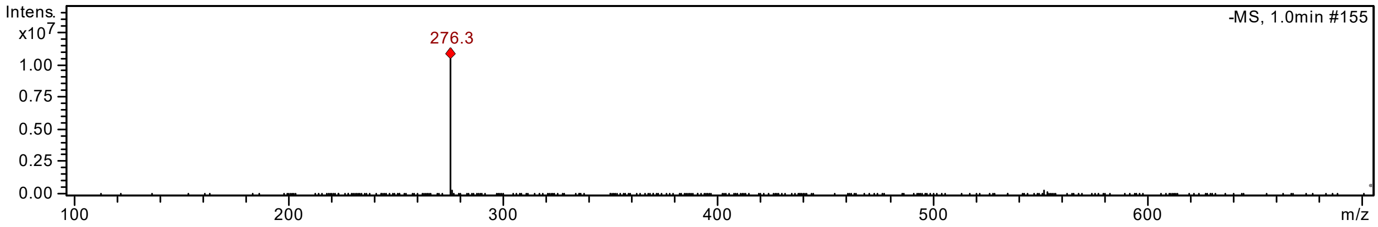

Figure S35. The MS spectrum for the compound **3g**.

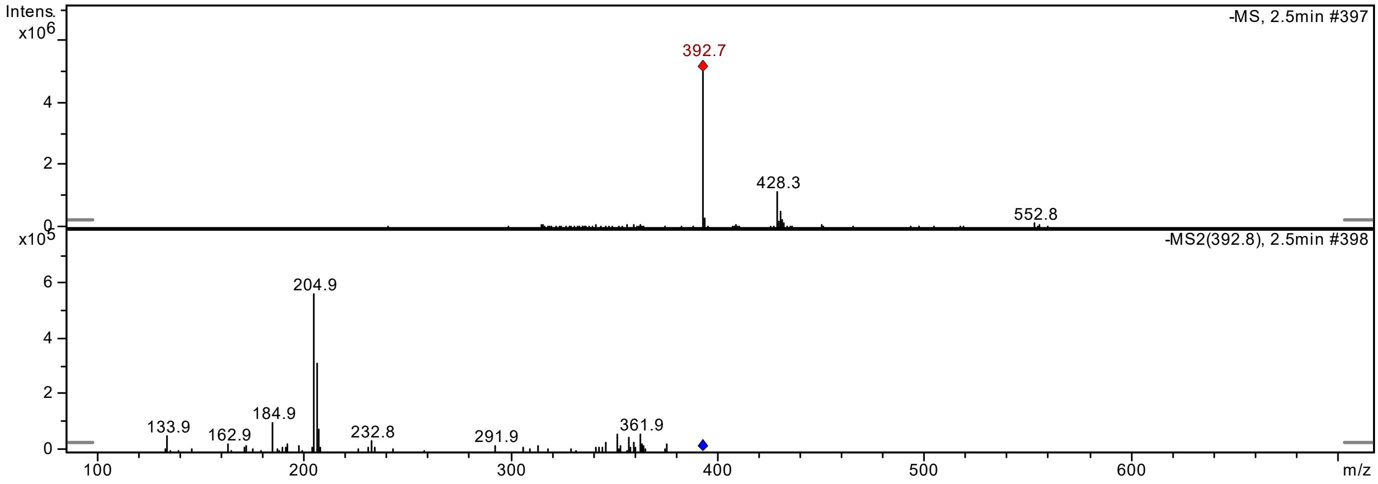

Figure S36. The MS spectrum for the compound **4a**.

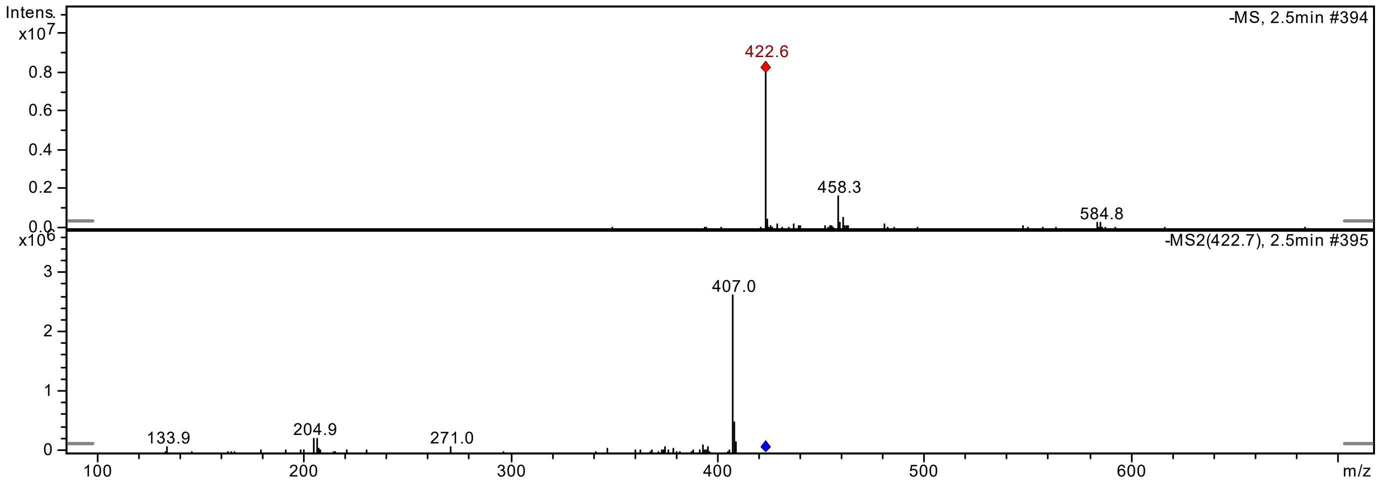

Figure S37. The MS spectrum for the compound **4b**.

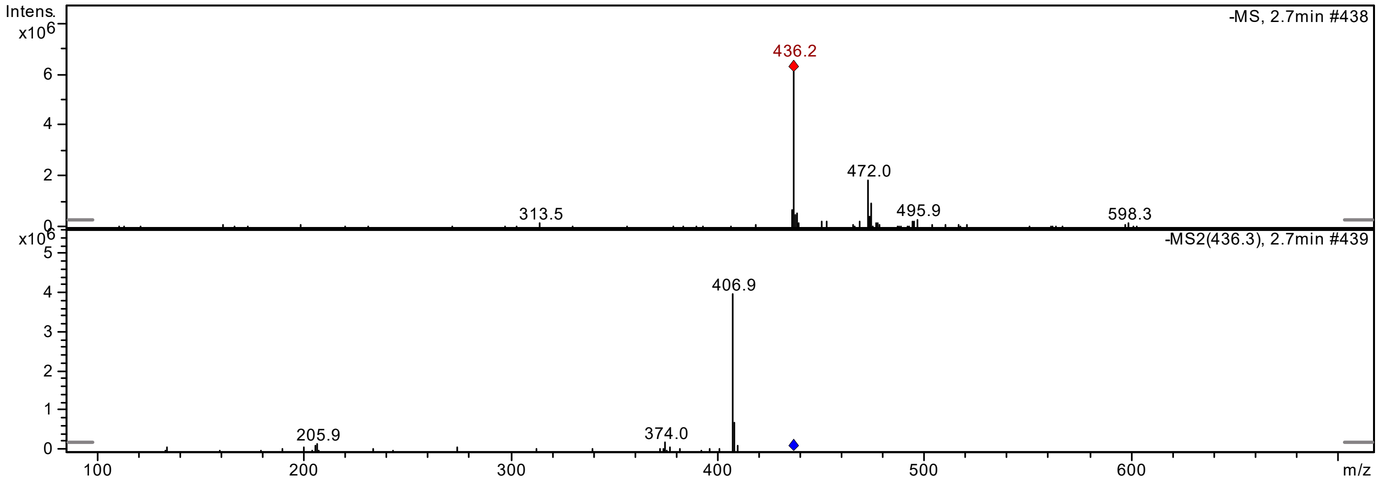

Figure S38. The MS spectrum for the compound **4c**.

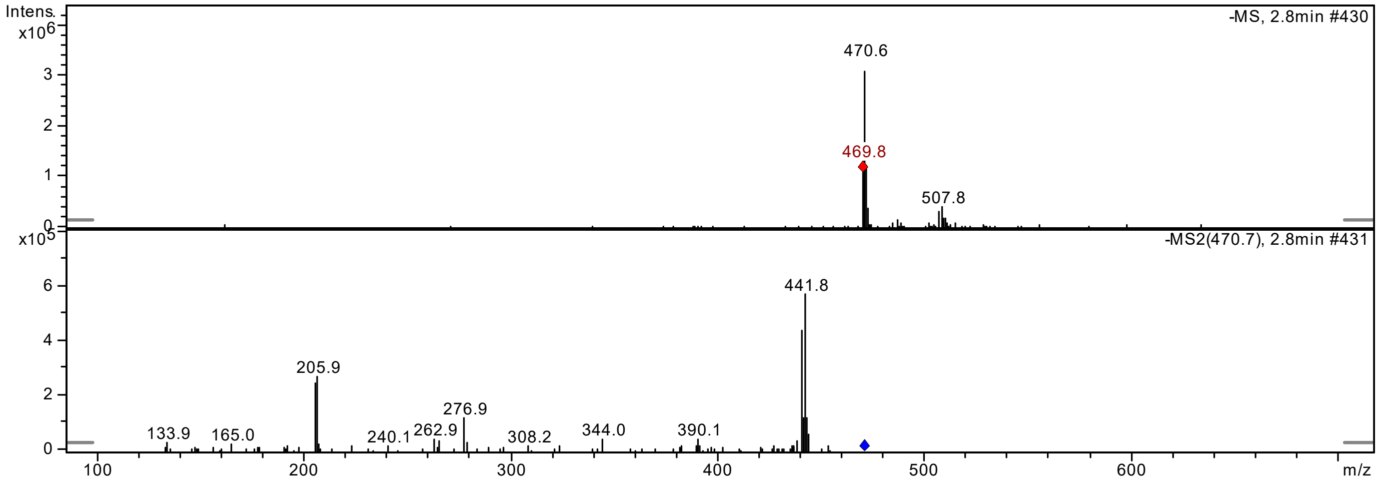

Figure S39. The MS spectrum for the compound **4d**.

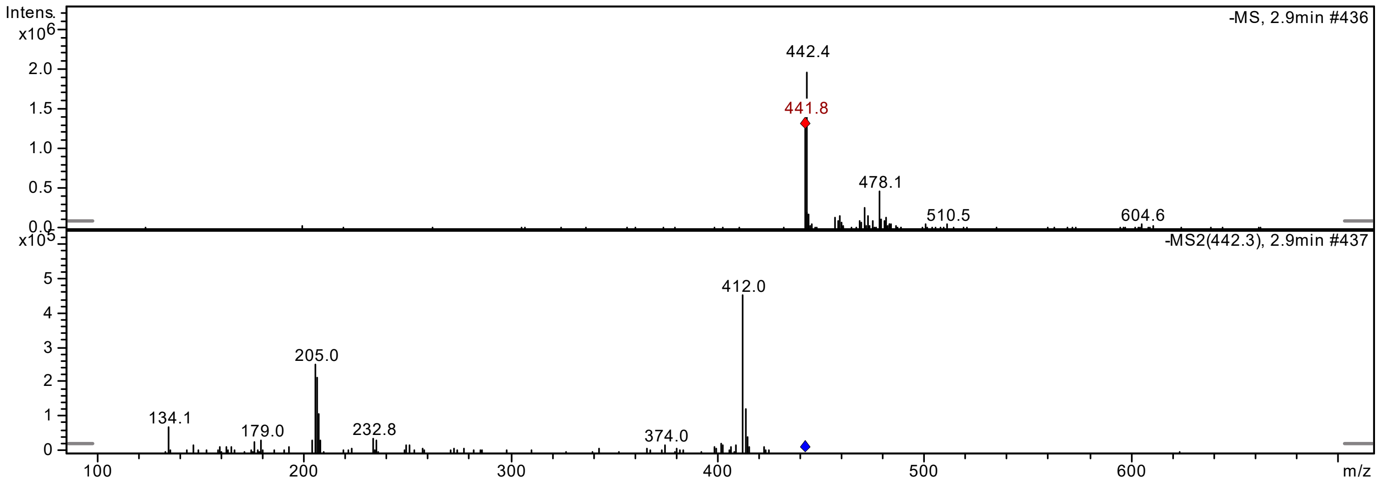

Figure S40. The MS spectrum for the compound **4e**.

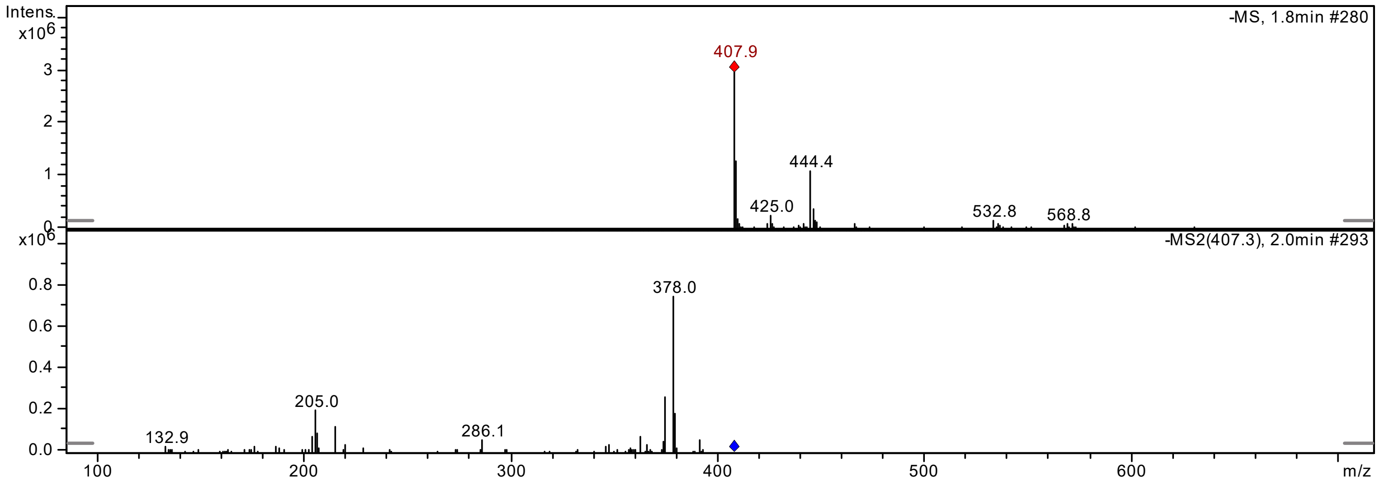

Figure S41. The MS spectrum for the compound **4f**.

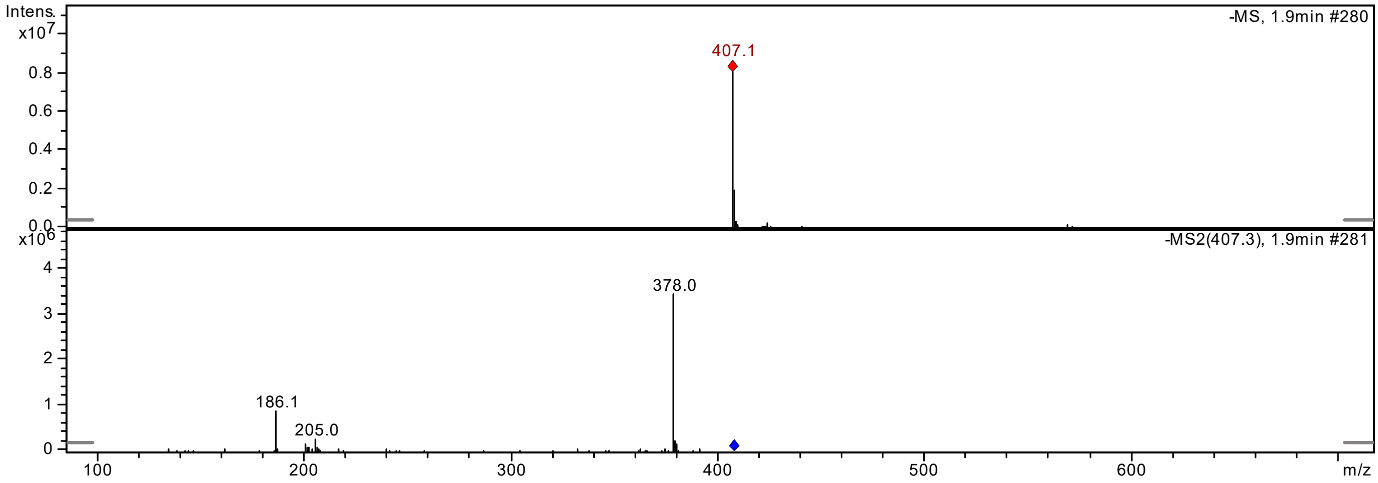

Figure S42. The MS spectrum for the compound **4g**.

1.3. ^{1}H-NMR Spectra

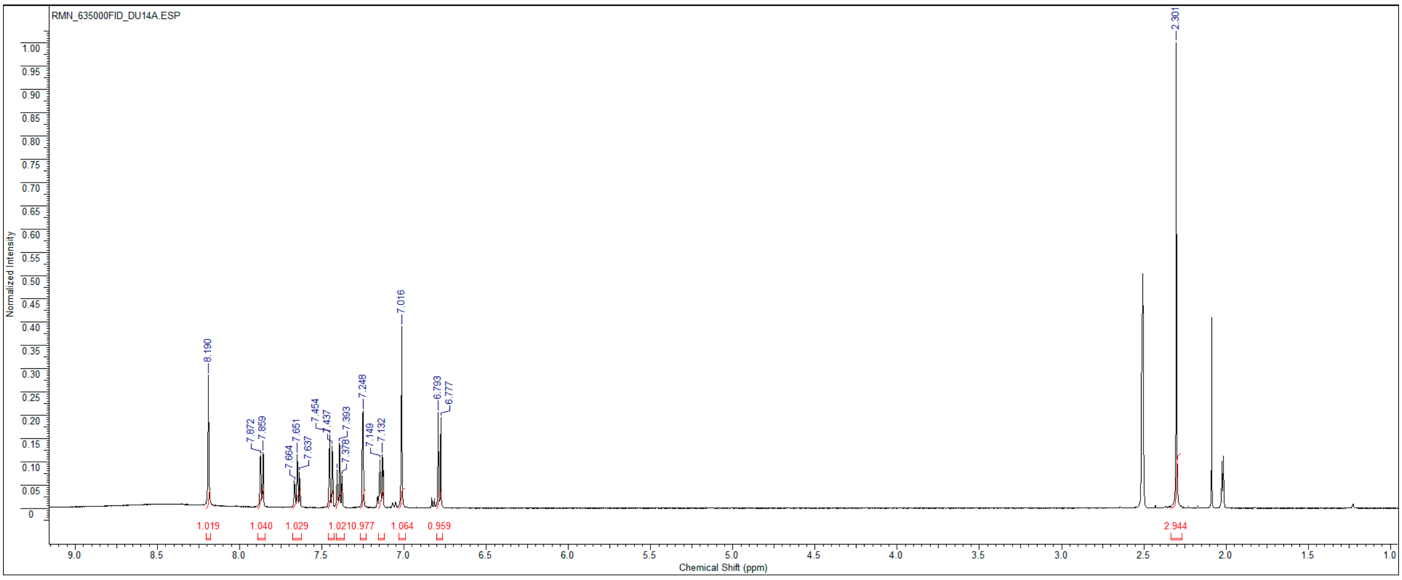

Figure S43. The ^{1}H-NMR spectrum for the compound **4a**.

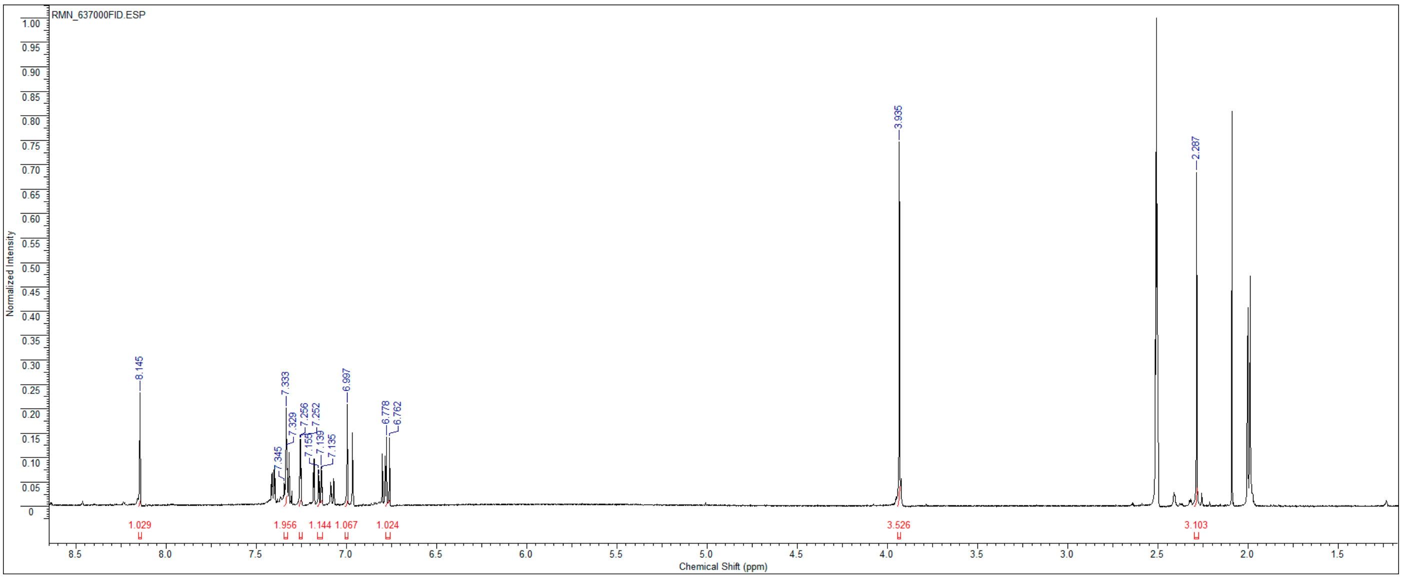

Figure S44. The ^{1}H-NMR spectrum for the compound **4b**.

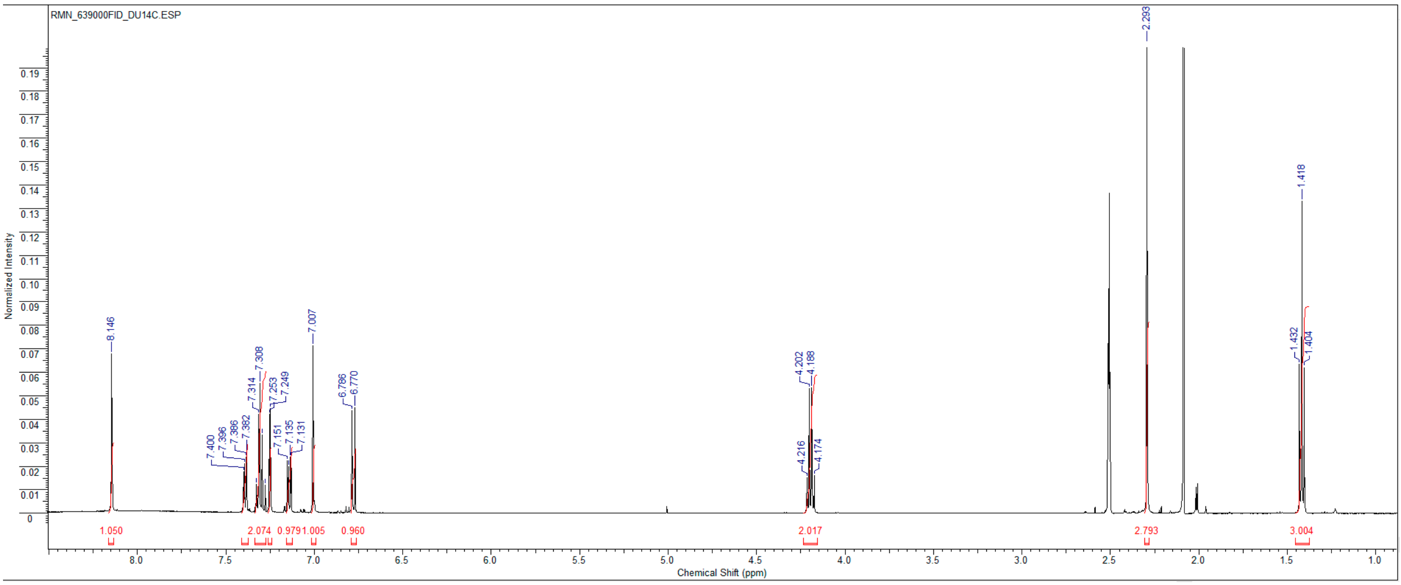

Figure S45. The ^{1}H-NMR spectrum for the compound **4c**.

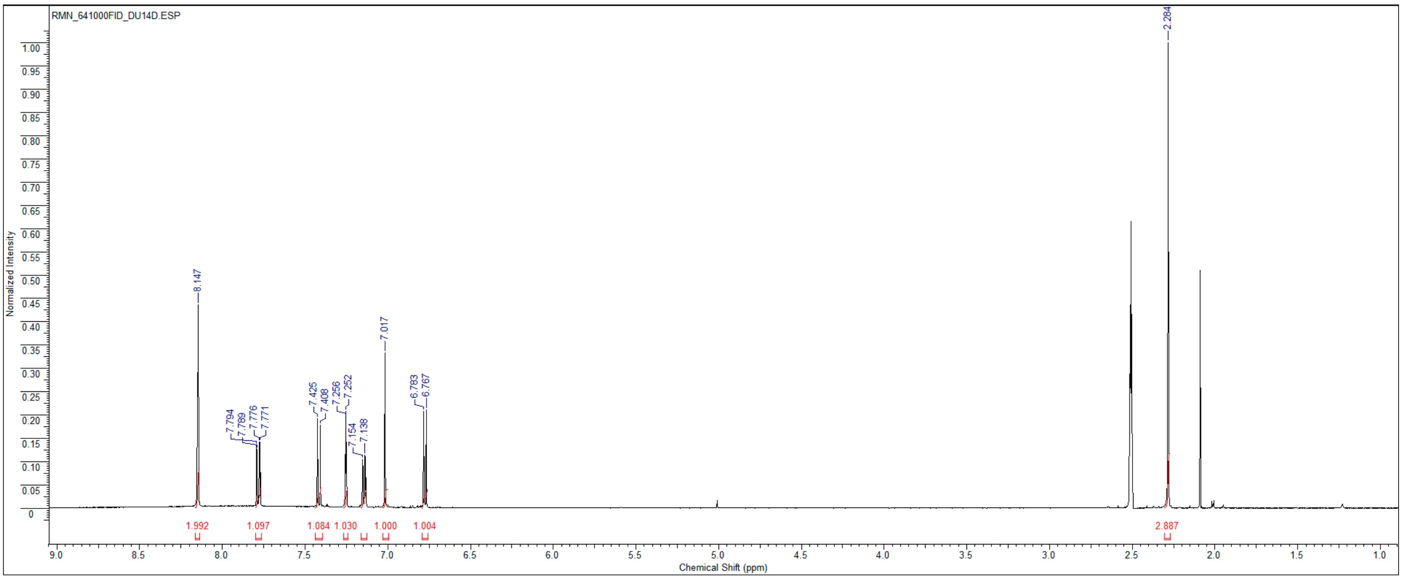

Figure S46. The ^{1}H-NMR spectrum for the compound **4d.**

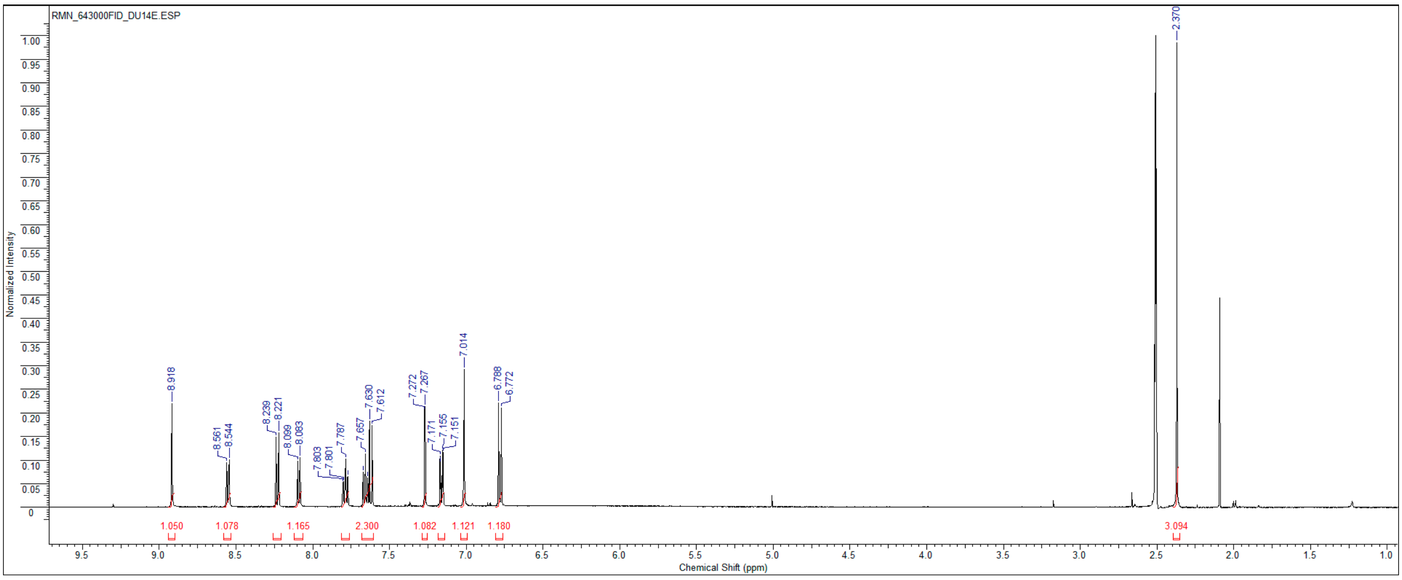

Figure S47. The ^{1}H-NMR spectrum for the compound **4e**.

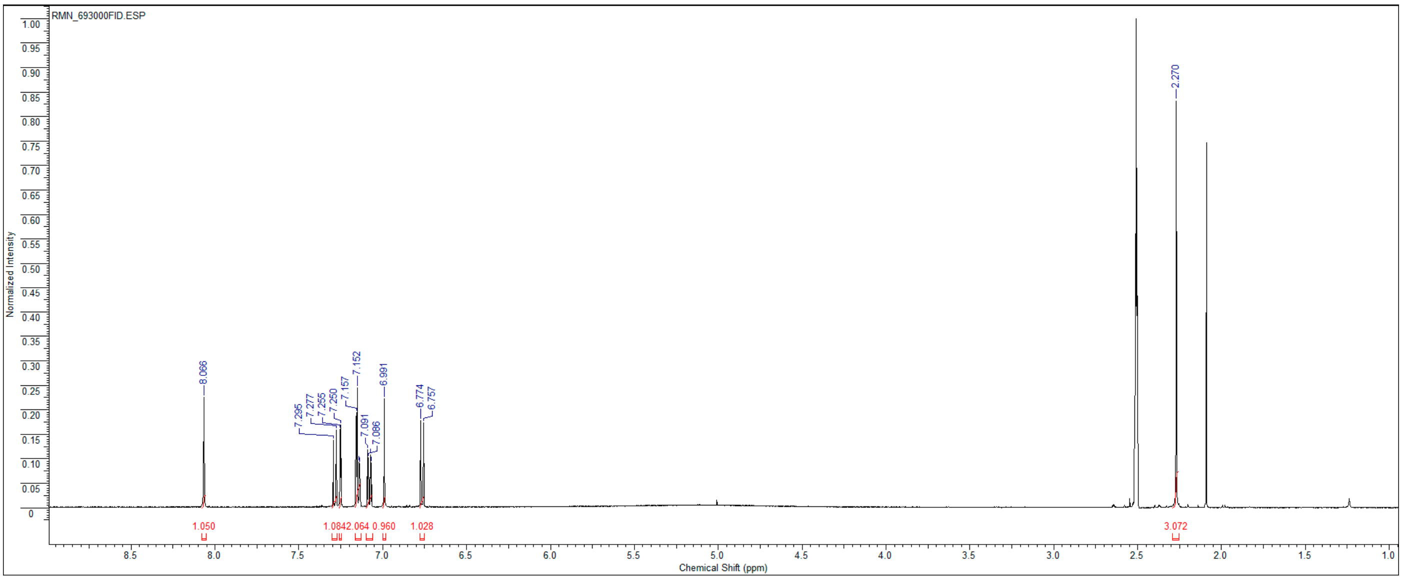

Figure S48. The ^{1}H-NMR spectrum for the compound **4f**.

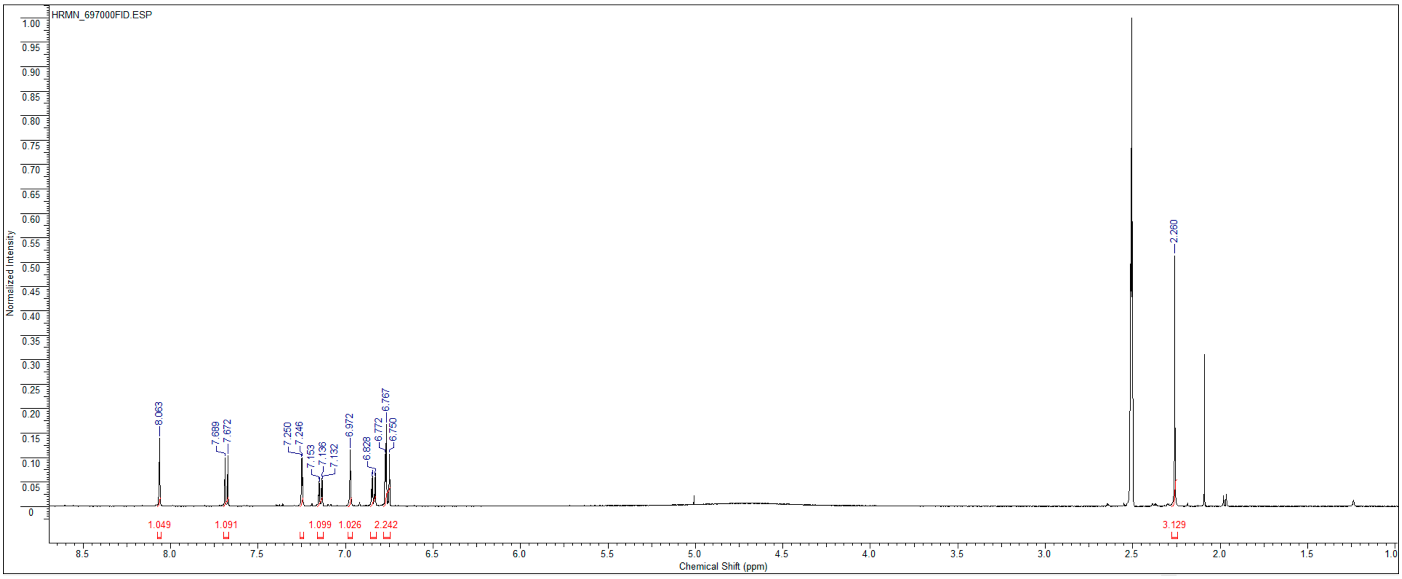

Figure S49. The ^{1}H-NMR spectrum for the compound **4g**.

1.4. ^{13}C-NMR Spectra

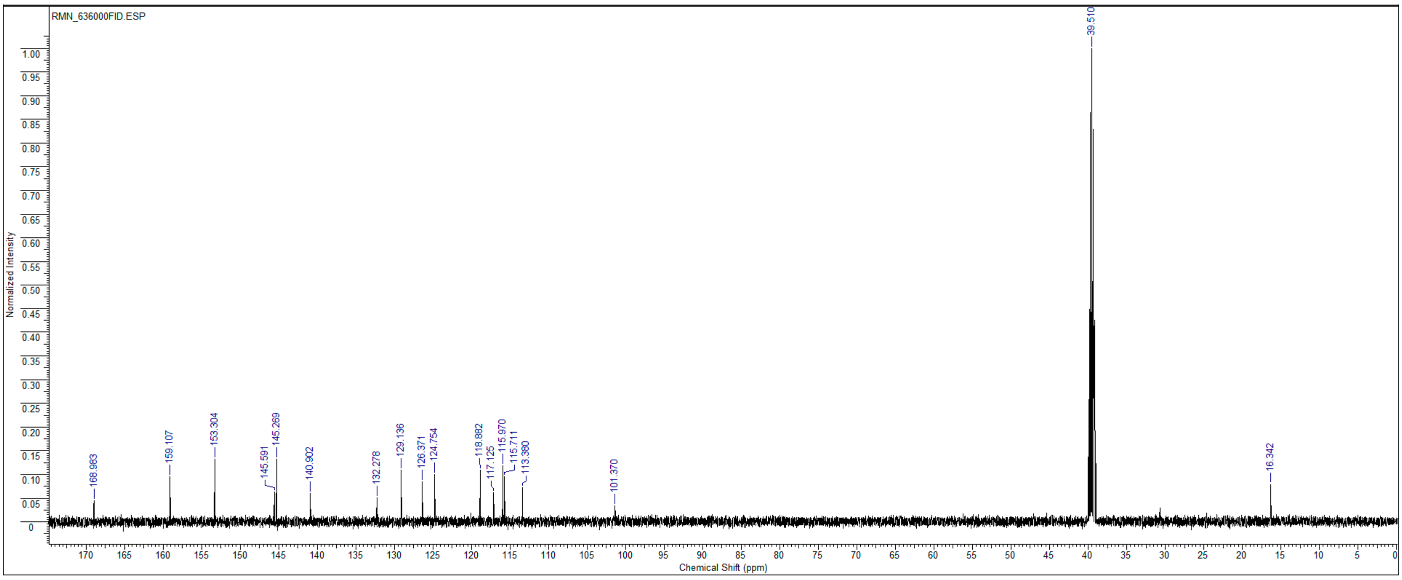

Figure S50. The ^{13}C-NMR spectrum for the compound **4a**.

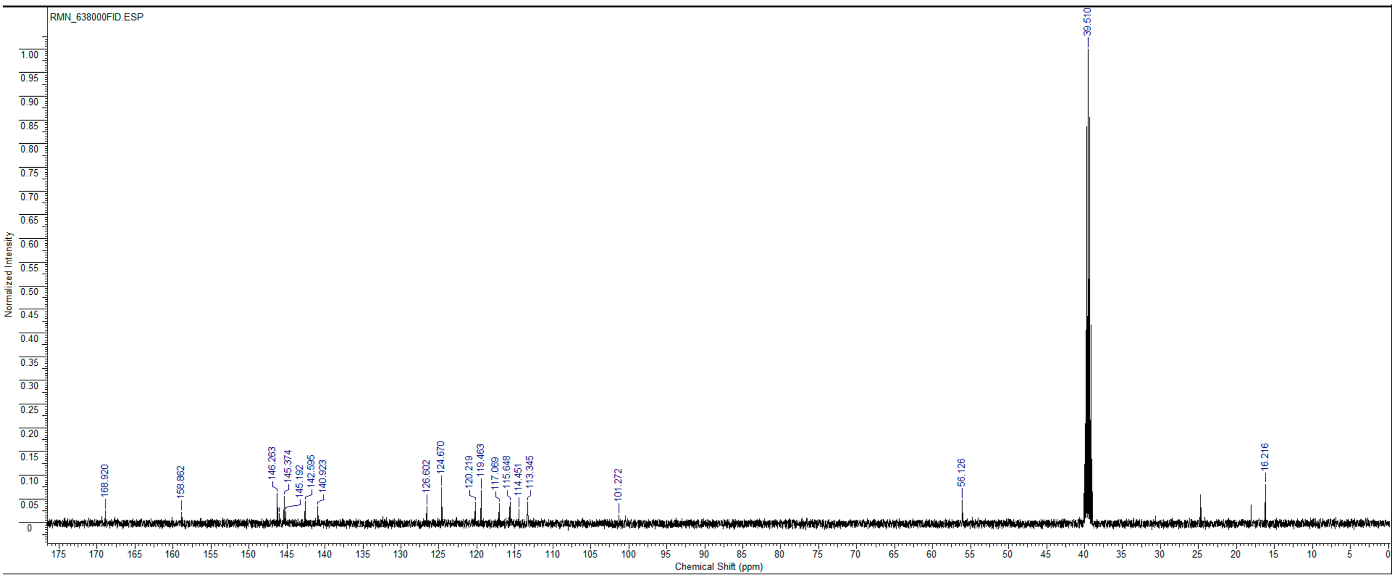

Figure S51. The ^{13}C-NMR spectrum for the compound **4b**.

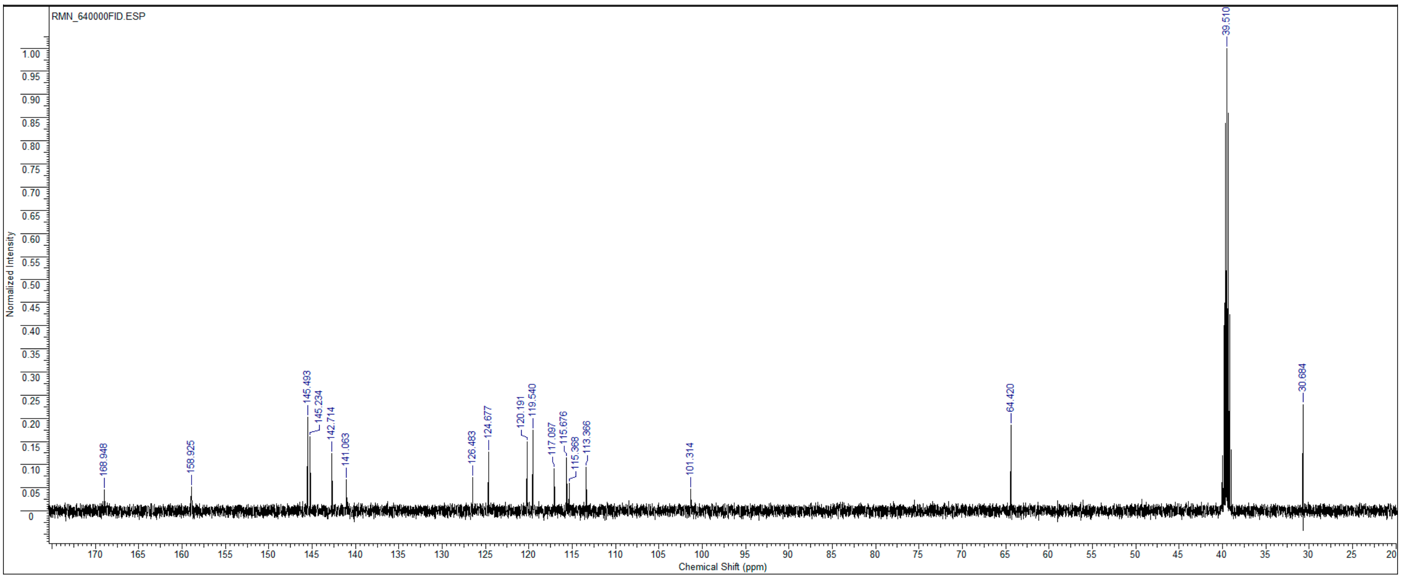

Figure S52. The ^{13}C-NMR spectrum for the compound **4c**.

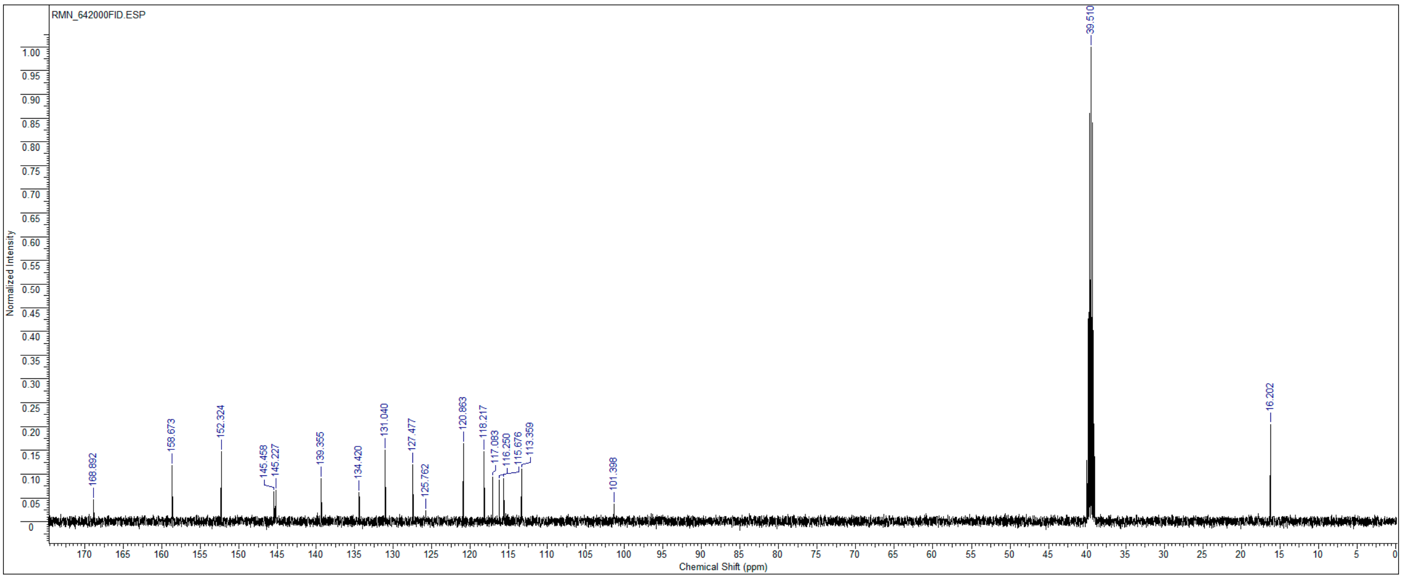

Figure S53. The ^{13}C-NMR spectrum for the compound **4d**.

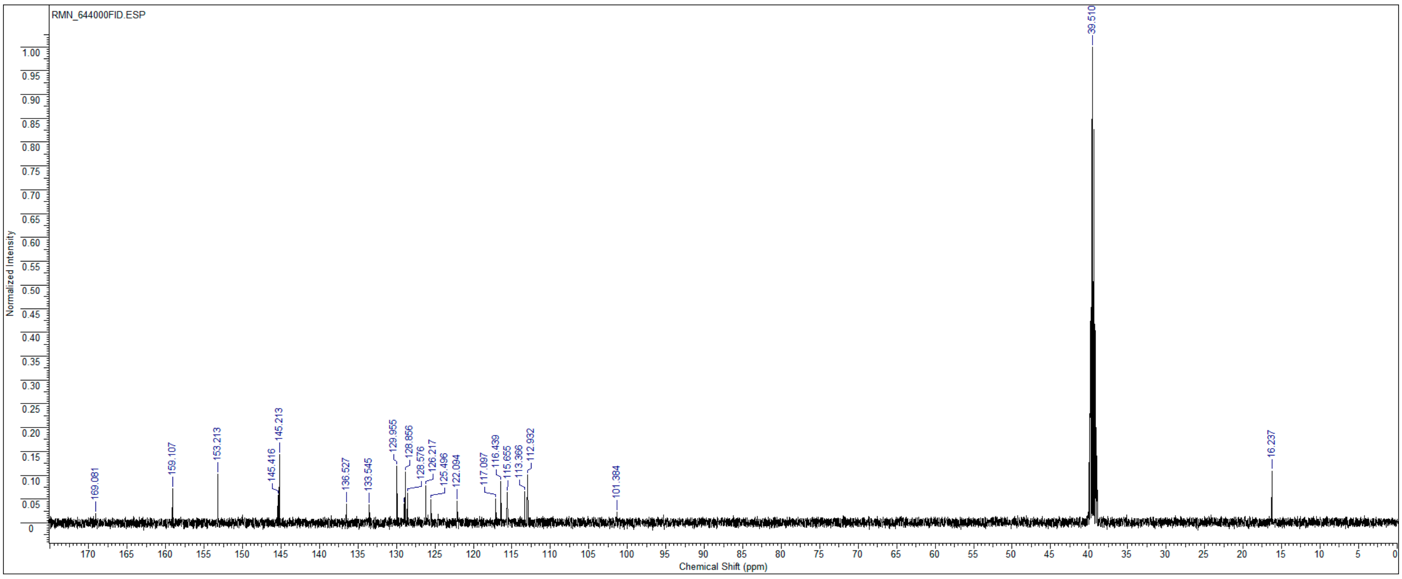

Figure S54. The ^{13}C-NMR spectrum for the compound **4e**.

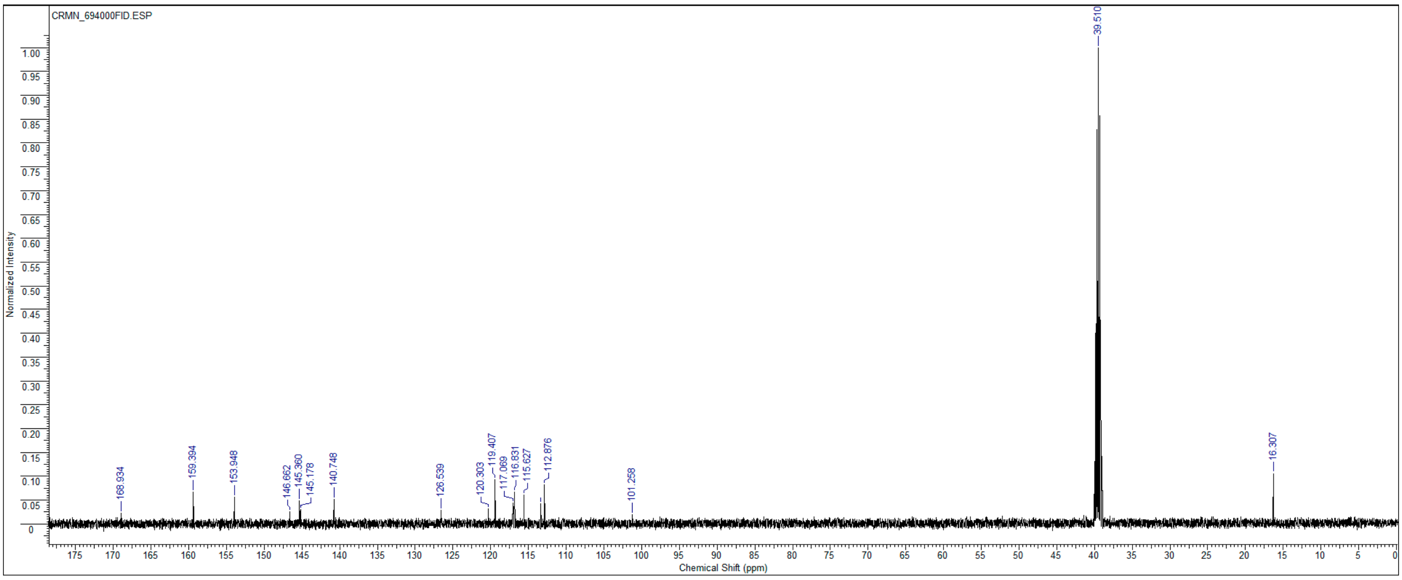

Figure S55. The ^{13}C-NMR spectrum for the compound **4f**.

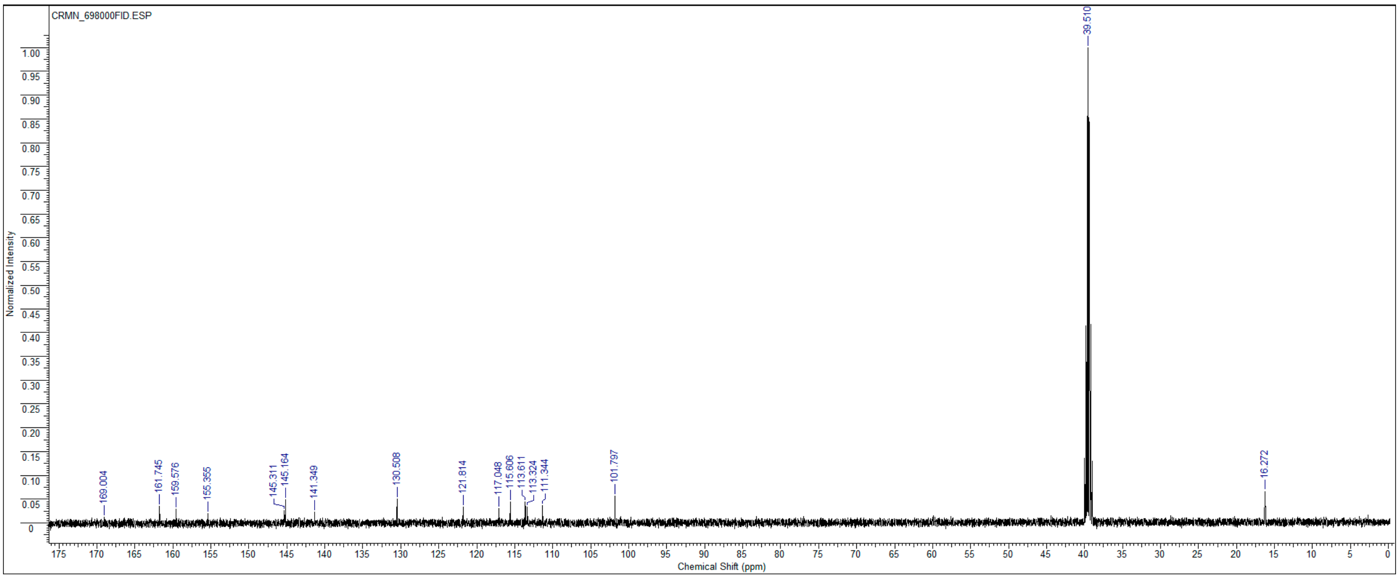

Figure S56. The ^{13}C-NMR spectrum for the compound **4g**.

1.5. Spin Density Maps

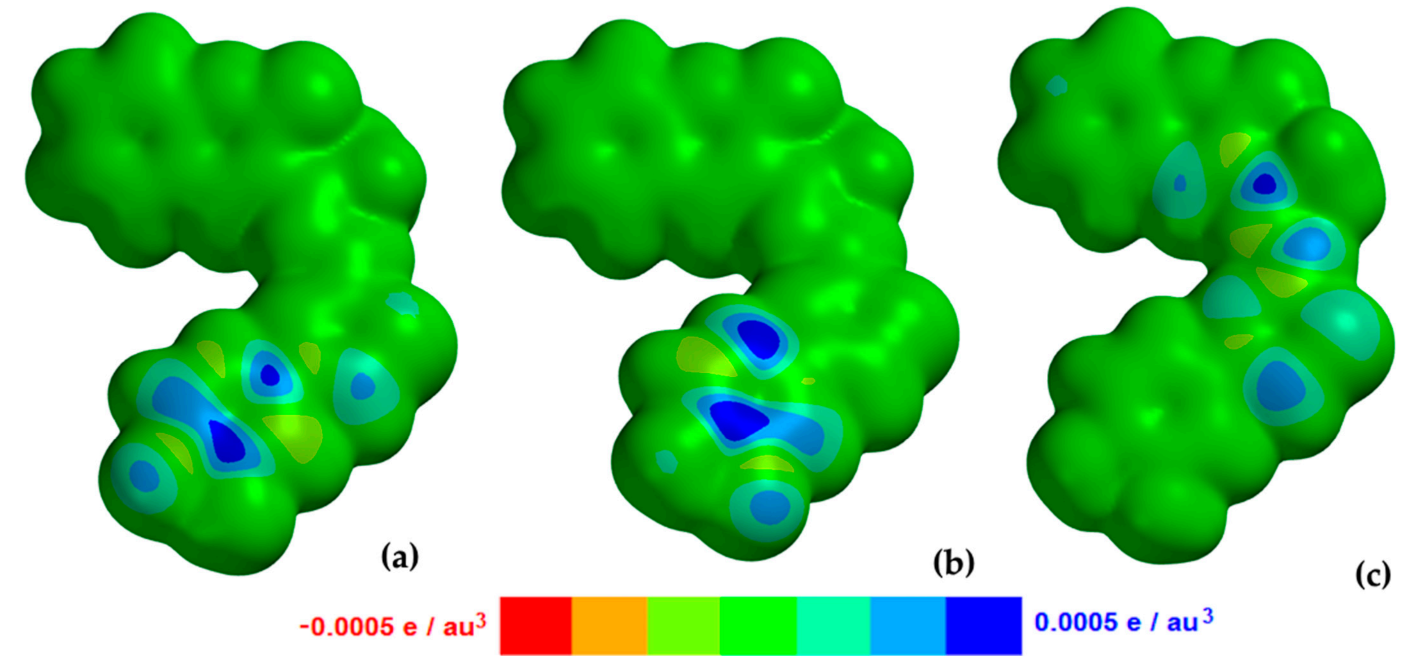

Figure S57. Spin density maps for compound **4a** depending on: **a)** 4′-OH radical; **b)** 3′-OH radical; **c)** hydrazone radical.

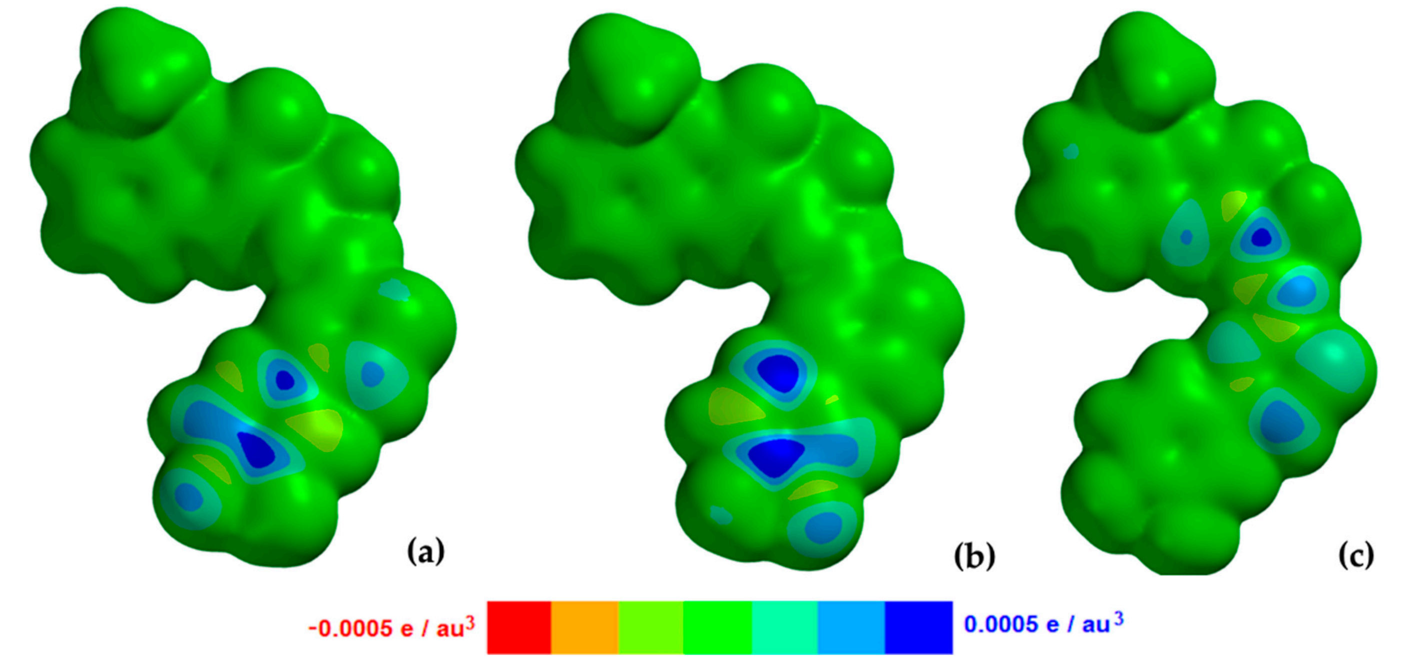

Figure S58. Spin density maps for compound **4b** depending on: **a)** 4′-OH radical; **b)** 3′-OH radical; **c)** hydrazone radical.

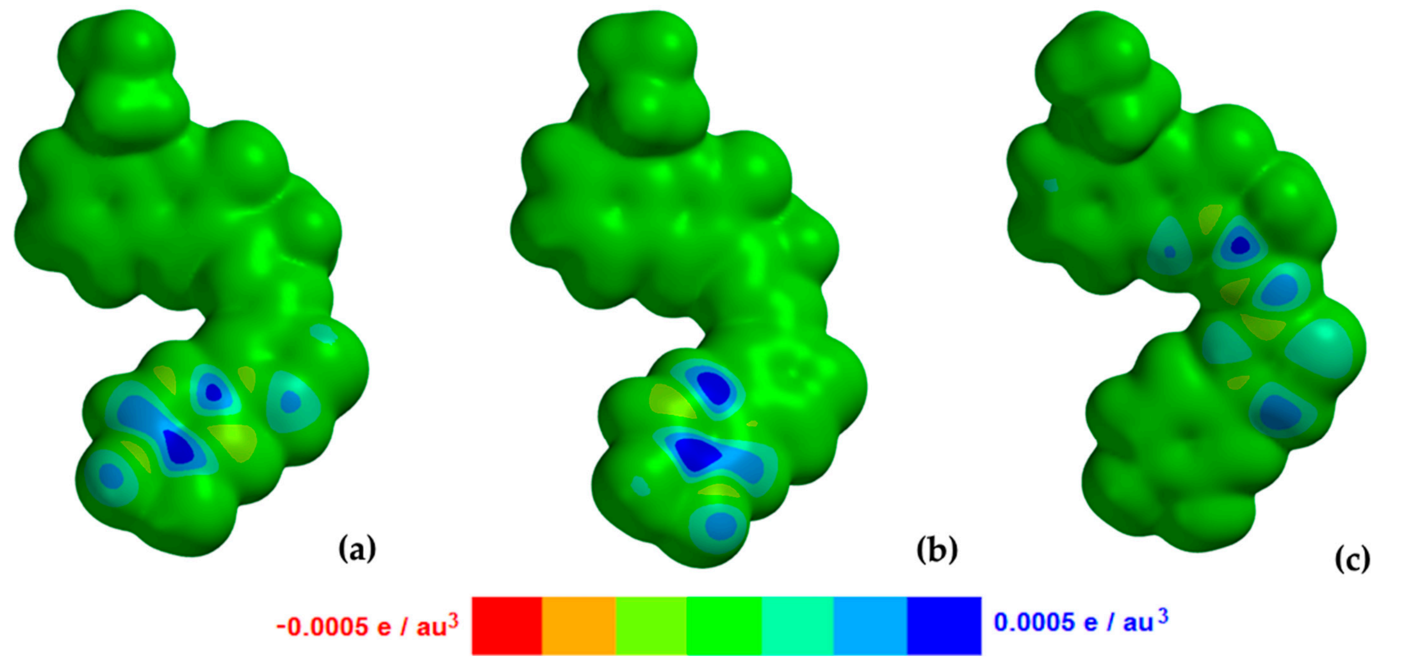

Figure S59. Spin density maps for compound **4c** depending on: **a)** 4′-OH radical; **b)** 3′-OH radical; **c)** hydrazone radical.

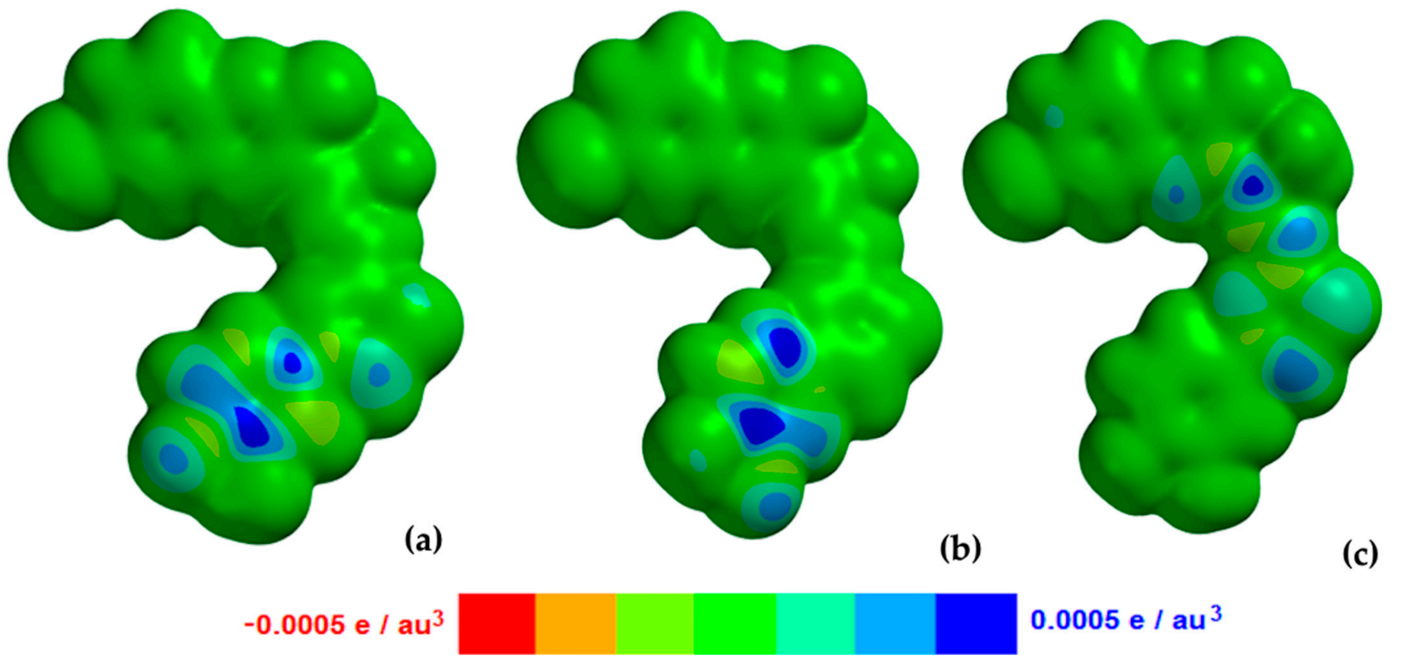

Figure S60. Spin density maps for compound **4d** depending on: **a)** 4′-OH radical; **b)** 3′-OH radical; **c)** hydrazone radical.

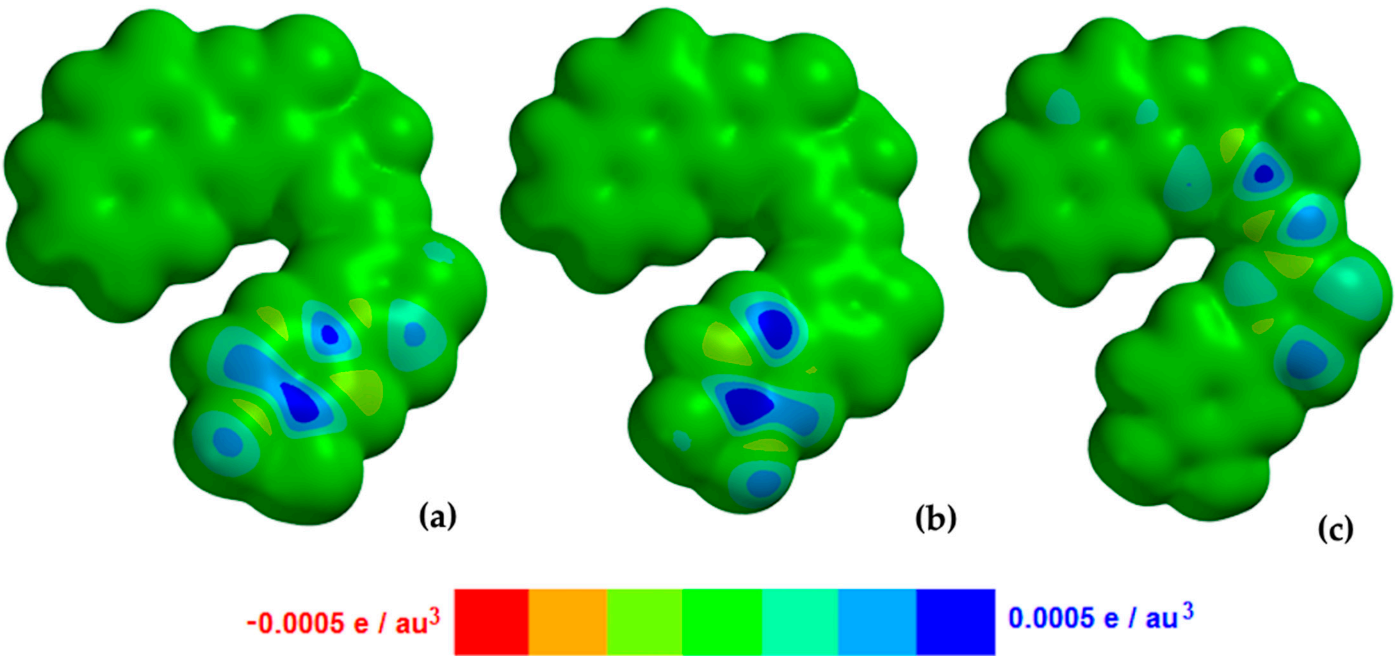

Figure S61. Spin density maps for compound **4e** depending on: **a)** 4′-OH radical; **b)** 3′-OH radical; **c)** hydrazone radical.

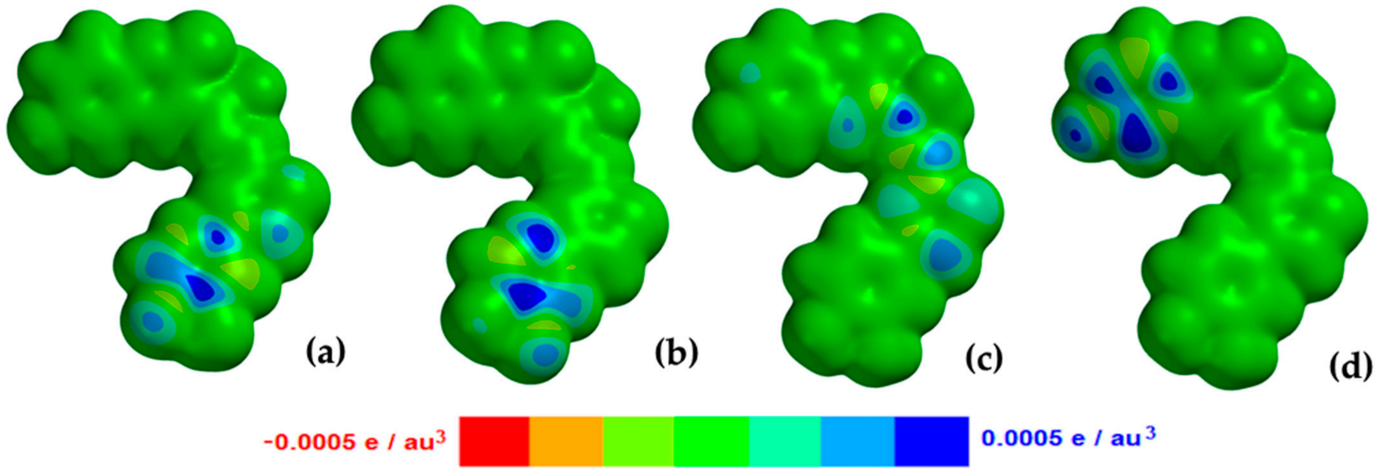

Figure S62. Spin density maps for compound **4f** depending on: **a)** 4′-OH radical; **b)** 3′-OH radical; **c)** hydrazone radical; **d)** 6-OH radical.

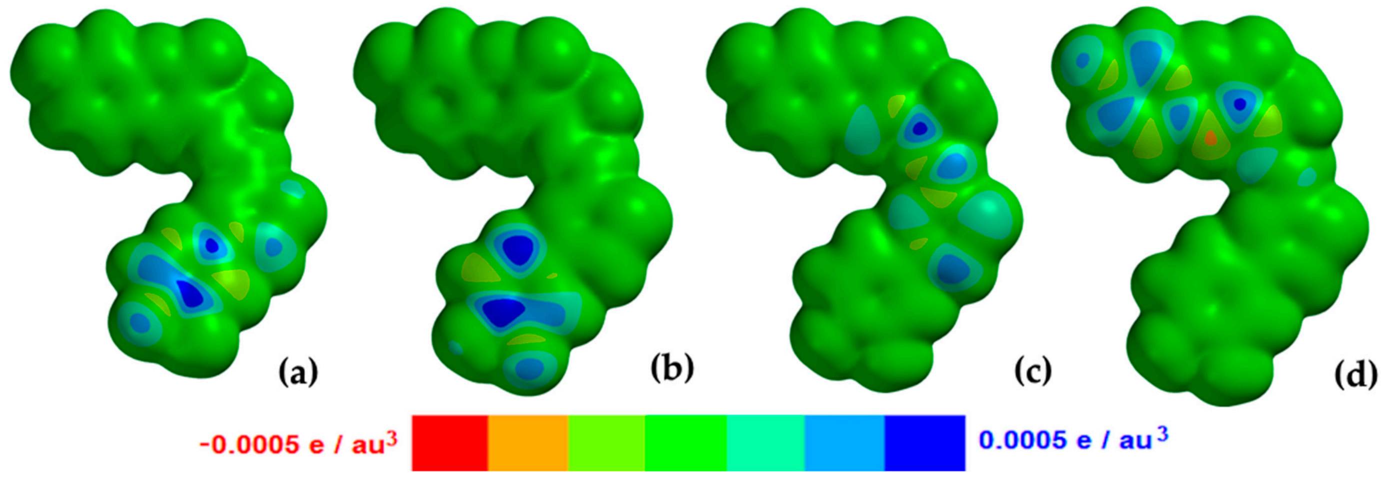

Figure S63. Spin density maps for compound **4g** depending on: **a)** 4′-OH radical; **b)** 3′-OH radical; **c)** hydrazone radical; **d)** 7-OH radical.

1.6. Depictions of HOMO and LUMO and Electrostatic Potential Maps

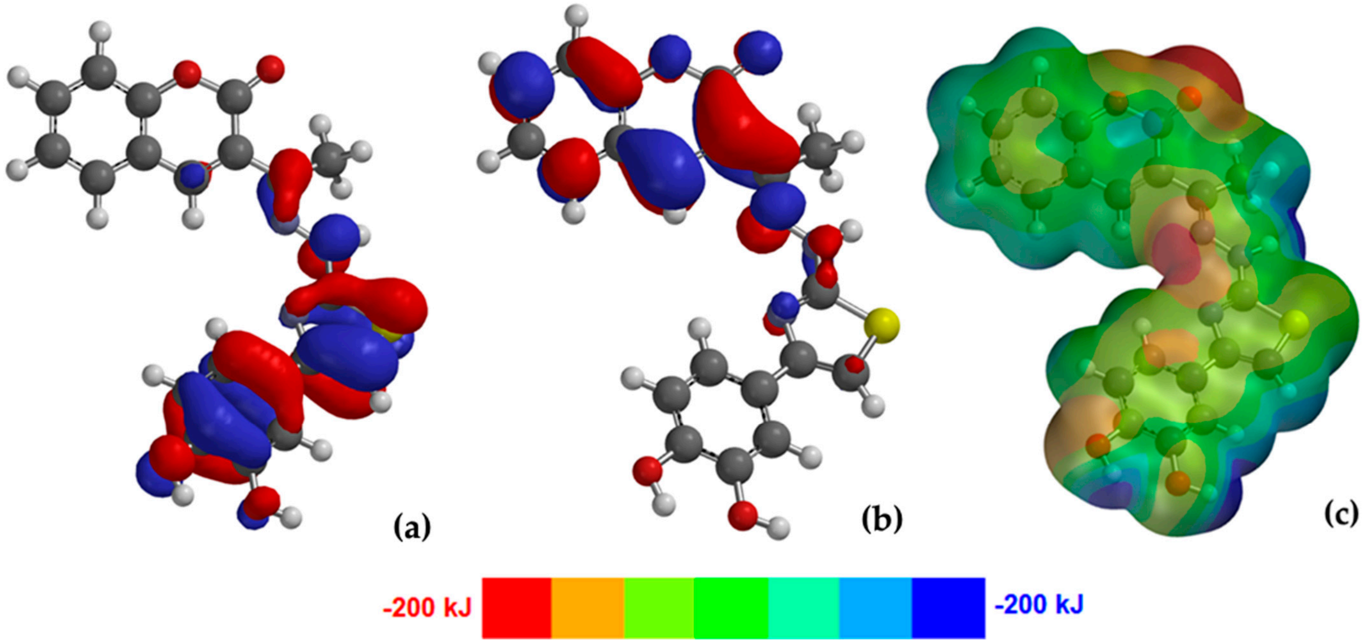

Figure S64. Depictions of HOMO (**a**) and LUMO (**b**) and the electrostatic potential map (**c**) for compound **4a**. HOMO was identified over the thiazolyl-catechol region, while LUMO was identified over the 2*H*-chromen-2-one heterocycle.

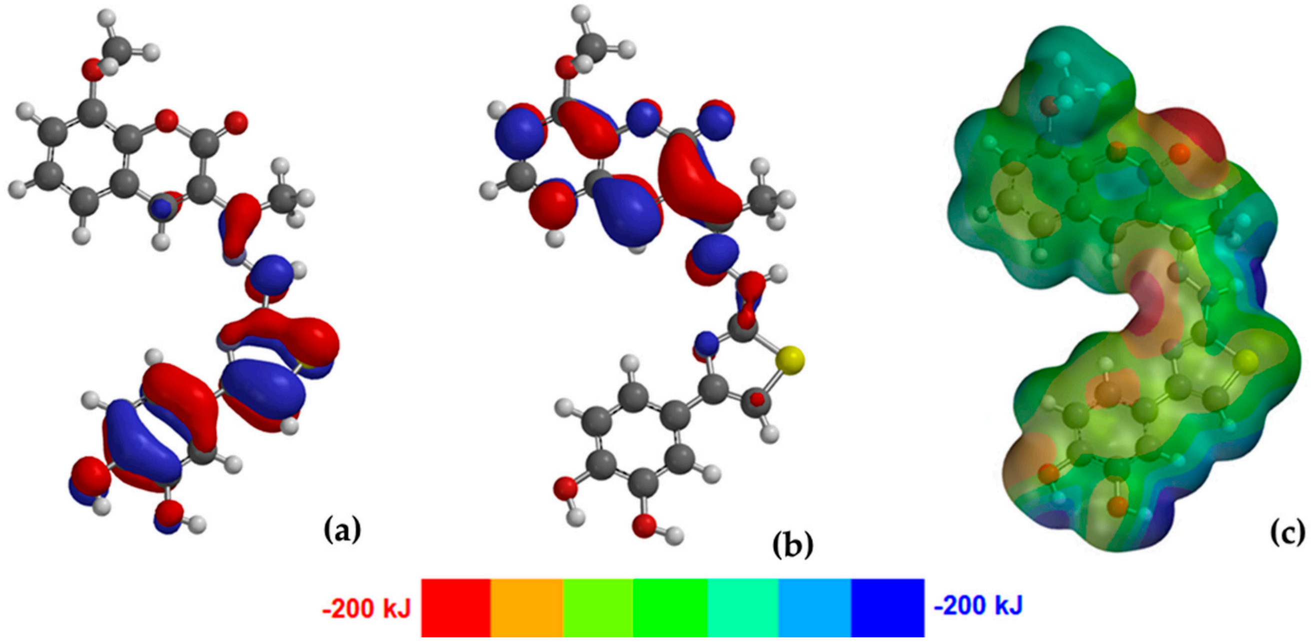

Figure S65. Depictions of HOMO (**a**) and LUMO (**b**) and the electrostatic potential map (**c**) for compound **4b**. HOMO was identified over the thiazolyl-catechol region, while LUMO was identified over the 2*H*-chromen-2-one heterocycle.

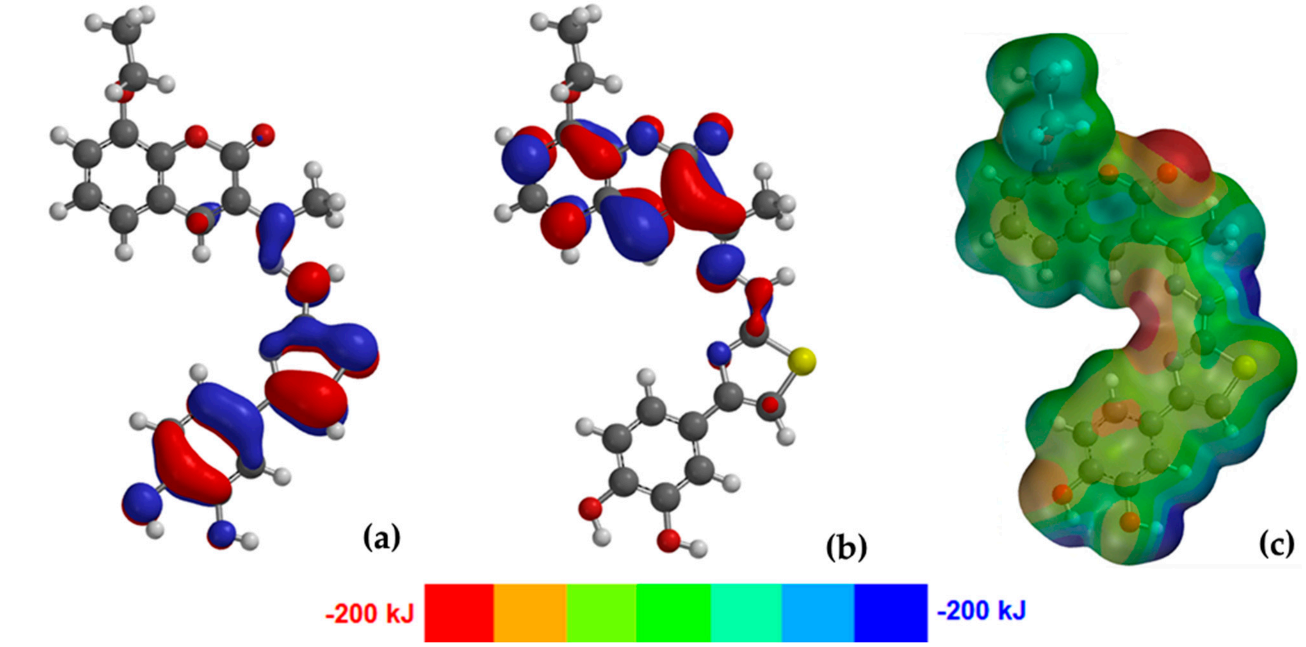

Figure S66. Depictions of HOMO (**a**) and LUMO (**b**) and the electrostatic potential map (**c**) for compound **4c**. HOMO was identified over the thiazolyl-catechol region, while LUMO was identified over the 2*H*-chromen-2-one heterocycle.

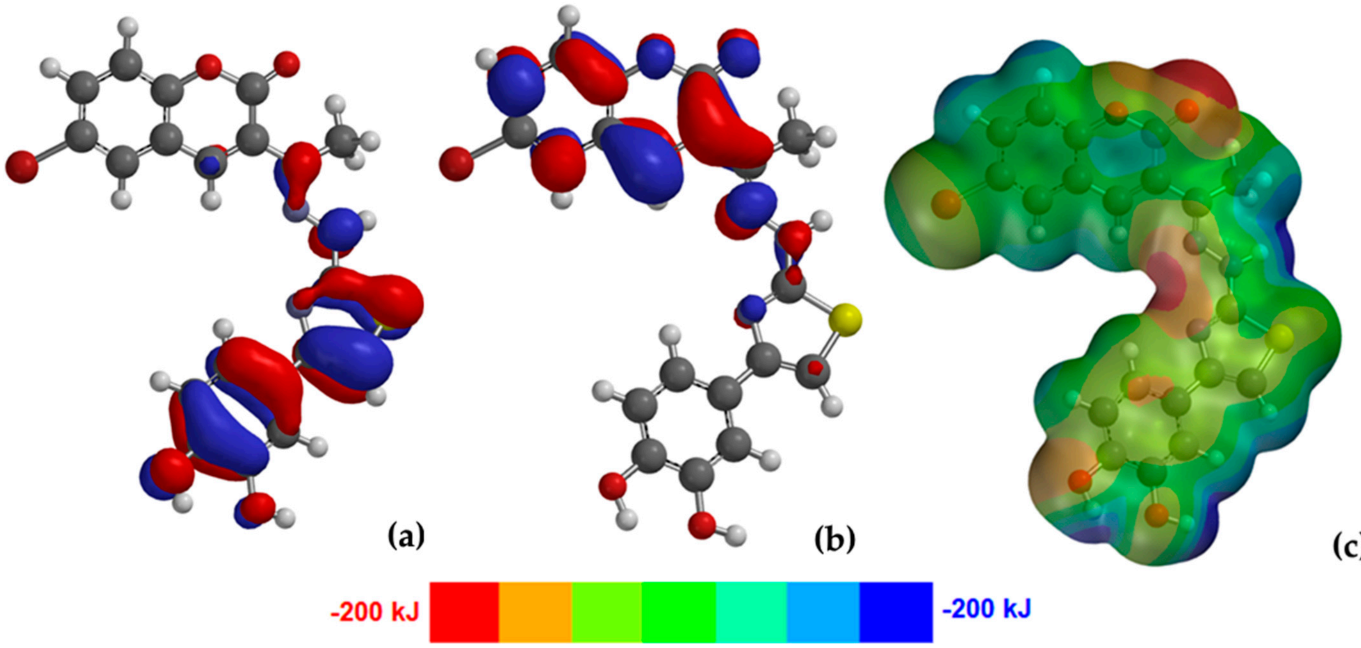

Figure S67. Depictions of HOMO (**a**) and LUMO (**b**) and the electrostatic potential map (**c**) for compound **4d**. HOMO was identified over the thiazolyl-catechol region, while LUMO was identified over the 2*H*-chromen-2-one heterocycle.

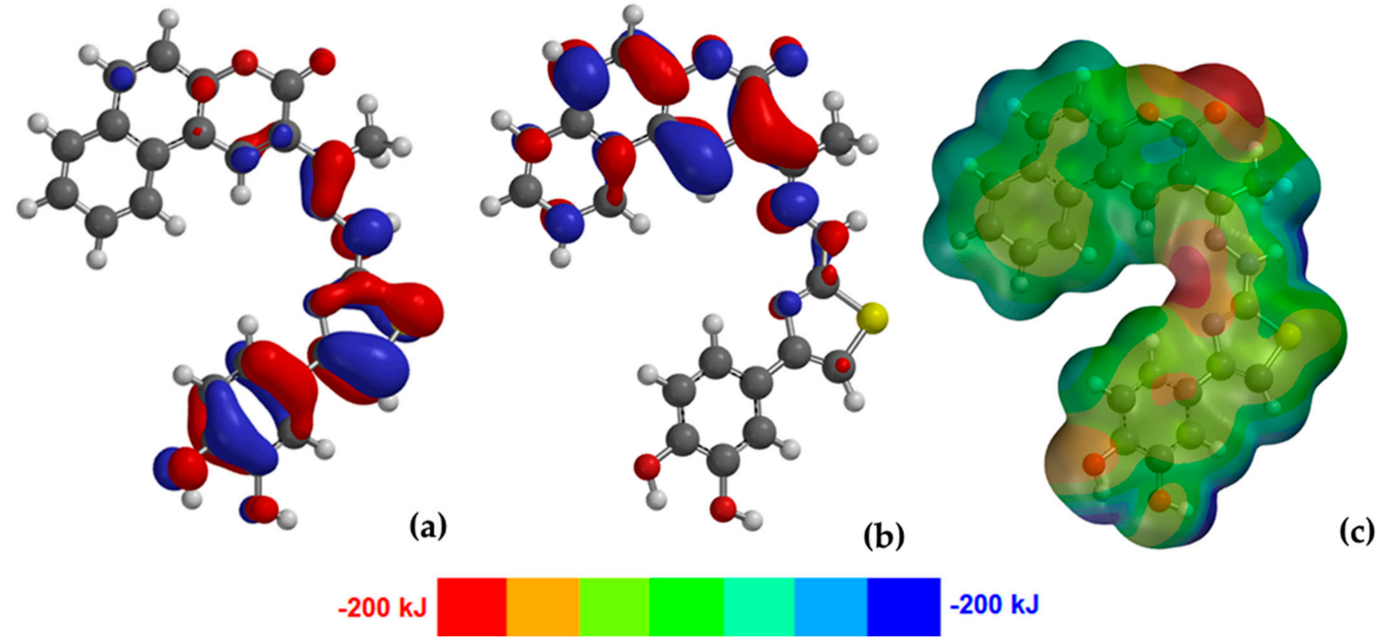

Figure S68. Depictions of HOMO (**a**) and LUMO (**b**) and the electrostatic potential map (**c**) for compound **4e**. HOMO was identified over the thiazolyl-catechol region, while LUMO was identified over the 2*H*-chromen-2-one heterocycle.

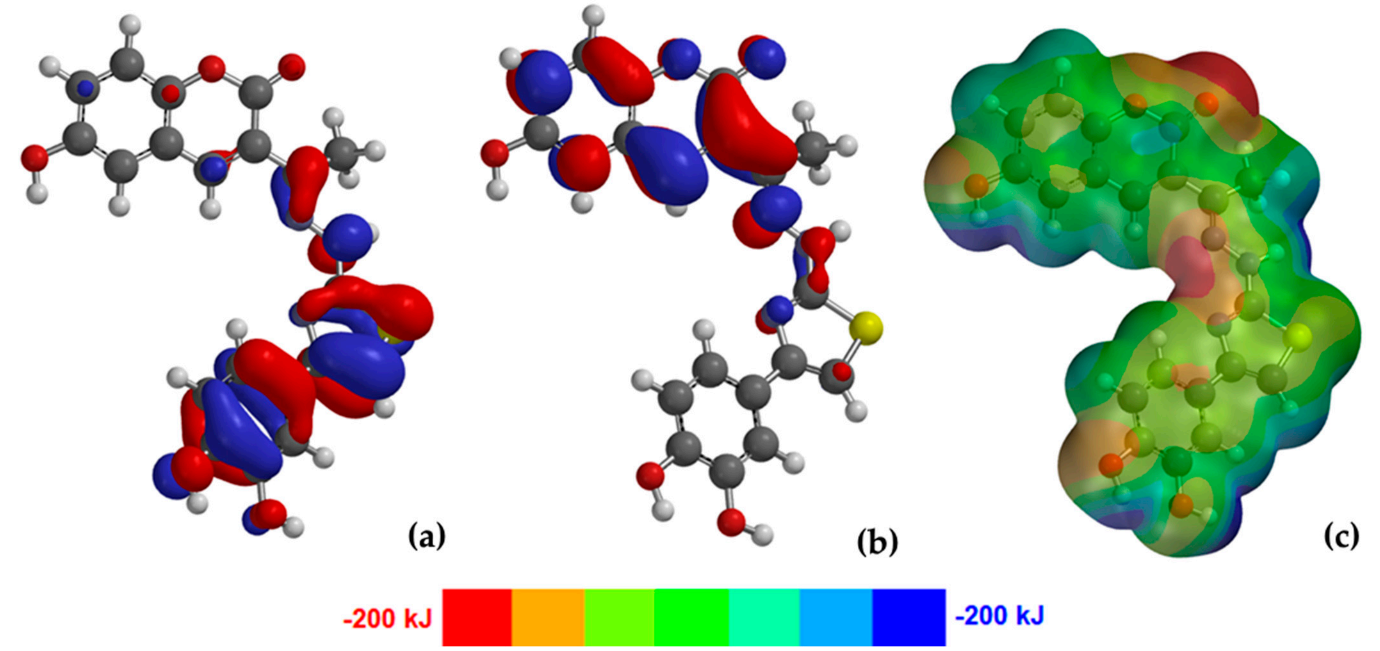

Figure S69. Depictions of HOMO (**a**) and LUMO (**b**) and the electrostatic potential map (**c**) for compound **4f**. HOMO was identified over the thiazolyl-catechol region, while LUMO was identified over the 2*H*-chromen-2-one heterocycle.

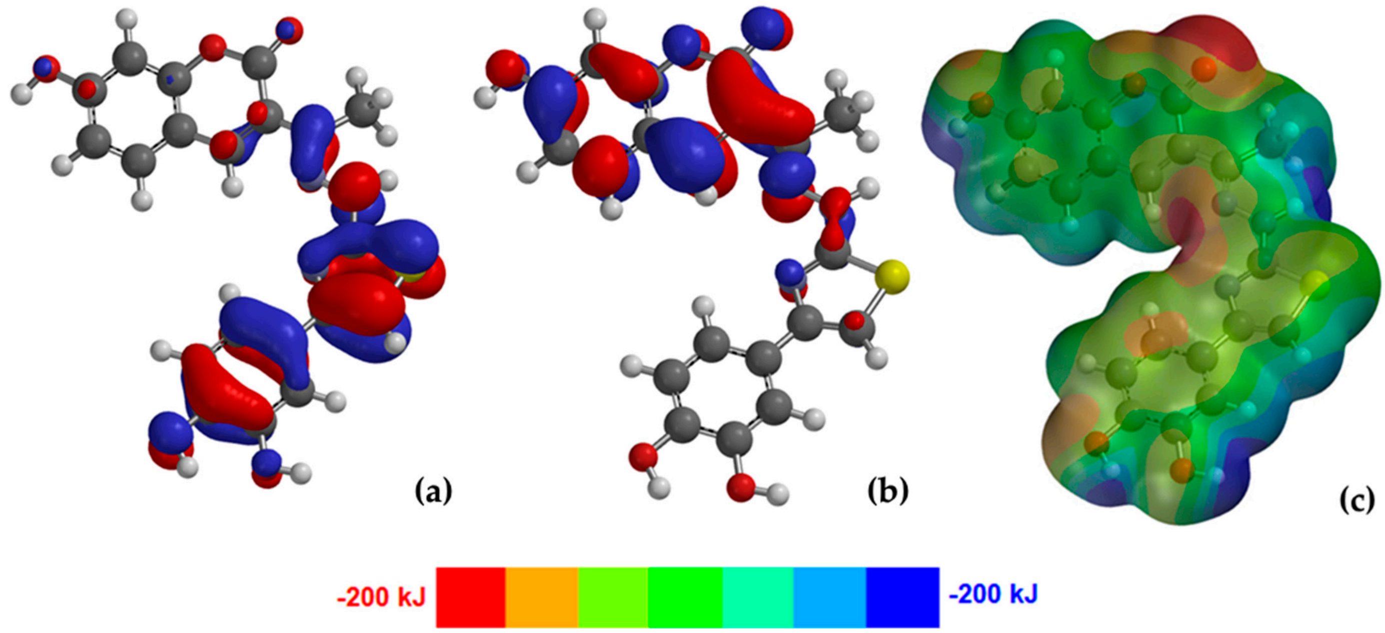

Figure S70. Depictions of HOMO (**a**) and LUMO (**b**) and the electrostatic potential map (**c**) for compound **4g**. HOMO was identified over the thiazolyl-catechol region, while LUMO was identified over the 2*H*-chromen-2-one heterocycle.
